# Characteristic bisimulation for higher-order session processes

**DOI:** 10.1007/s00236-016-0289-7

**Published:** 2016-12-24

**Authors:** Dimitrios Kouzapas, Jorge A. Pérez, Nobuko Yoshida

**Affiliations:** 1 University of Glasgow, Glasgow, UK; 2 University of Groningen, Groningen and CWI, Amsterdam, The Netherlands; 3 Imperial College London, London, UK

## Abstract

For higher-order (process) languages, characterising contextual equivalence is a long-standing issue. In the setting of a higher-order $\pi$-calculus with *session types*, we develop *characteristic bisimilarity*, a typed bisimilarity which fully characterises contextual equivalence. To our knowledge, ours is the first characterisation of its kind. Using simple values inhabiting (session) types, our approach distinguishes from untyped methods for characterising contextual equivalence in higher-order processes: we show that observing as inputs only a precise finite set of higher-order values suffices to reason about higher-order session processes. We demonstrate how characteristic bisimilarity can be used to justify optimisations in session protocols with mobile code communication.

## Introduction

*Context* In *higher-order process calculi* communicated values may contain processes. Higher-order concurrency has received significant attention from untyped and typed perspectives; see, e.g., [13, 15, 20, 26, 30, 33]. In this work, we consider $\textsf {HO}\pi$, a higher-order process calculus with *session communication*: it combines functional constructs (abstractions/applications, as in the call-by-value $\lambda$-calculus) and concurrent primitives (synchronisation on shared names, communication on linear names, recursion). By amalgamating functional and concurrent constructs, $\textsf {HO}\pi$ may specify complex session protocols that include both first-order communication (name passing) and higher-order processes (process passing) and that can be type-checked using *session types* [9]. By enforcing *shared* and *linear* usage policies, session types ensure that each communication channel in a process specification conforms to its prescribed protocol. In session-based concurrency, distinguishing between shared and linear names is important, for computation conceptually involves two distinct phases: the first one is non-deterministic and uses shared names, as it represents the interaction of processes seeking compatible protocol partners; the second phase proceeds deterministically along linear names, as it specifies the concurrent execution of the session protocols established in the first phase.

Although models of higher-order concurrency with session communication have been already developed (cf. works by Mostrous and Yoshida [25] and by Gay and Vasconcelos [5]), their *behavioural equivalences* remain little understood. Clarifying the status of these equivalences is essential to, e.g., justify non-trivial optimisations in protocols involving both name and process passing. An important aspect in the development of these typed equivalences is that typed semantics are usually *coarser* than untyped semantics. Indeed, since (session) types limit the contexts (environments) in which processes can interact, typed equivalences admit stronger properties than their untyped counterpart.

The form of contextual equivalence typically used in concurrency is *barbed congruence* [10, 24]. A well-known behavioural equivalence for higher-order processes is *context bisimilarity* [31]. This is a characterisation of barbed congruence that offers an adequate distinguishing power at the price of heavy universal quantifications in output clauses. Obtaining alternative characterisations of context bisimilarity is thus a recurring, important problem for higher-order calculi—see, e.g., [13, 15, 21, 30, 31, 34]. In particular, Sangiorgi [30, 31] has given characterisations of context bisimilarity for higher-order processes; such characterisations, however, do not scale to calculi with *recursive types*, which are essential to express practical protocols in session-based concurrency. A characterisation that solves this limitation was developed by Jeffrey and Rathke [13]; their solution, however, does not consider *linearity* which, as explained above, is an important aspect in session-based concurrency.

*This work* Building upon [13, 30, 31], our discovery is that linearity as induced by session types plays a vital rôle in solving the open problem of characterising context bisimilarity for higher-order mobile processes with session communication. Our approach is to exploit the coarser semantics induced by session types to limit the behaviour of higher-order session processes. Indeed, the use of session typed contexts (i.e., environments disciplined by session types) leads to process semantics that admit stronger properties than untyped semantics. Formally, we enforce this limitation in behaviour by defining a *refined* labelled transition system (LTS) which effectively narrows down the spectrum of allowed process behaviours, exploiting elementary processes inhabiting session types. We then introduce *characteristic bisimilarity*: this new notion of typed bisimilarity is *more tractable* than context bisimilarity, in that it relies on the refined LTS for input actions and, more importantly, does not appeal to universal quantifications on output actions.

Our main result is that characteristic bisimilarity coincides with context bisimilarity. Besides confirming the value of characteristic bisimilarity as a useful reasoning technique for higher-order processes with sessions, this result is remarkable also from a technical perspective, for associated completeness proofs do not require operators for name matching, in contrast to Jeffrey and Rathke’s technique for higher-order processes with recursive types [13].

*Outline* Next, we informally overview the key ideas of characteristic bisimilarity, our characterisation of contextual equivalence. Then, Sect. 3 presents the session calculus $\textsf {HO}\pi$. Section 4 gives the session type system for $\textsf {HO}\pi$ and states type soundness. Section 5 develops *characteristic* bisimilarity and states our main result: characteristic bisimilarity and contextual equivalence coincide for well-typed $\textsf {HO}\pi$ processes (Theorem 2). Section 6 discusses related works, while Sect. 7 collects some concluding remarks.

This paper is a revised, extended version of the conference paper [16]. This presentation includes full technical details—definitions and proofs, collected in Appendices 1 and 2. In particular, we introduce *higher-order bisimilarity* (an auxiliary labelled bisimilarity) and highlight its rôle in the proof of Theorem 2. We also elaborate further on the use case scenario for characteristic bisimilarity given in [16] (the Hotel Booking scenario). Using an additional example, given in Sect. 6, we compare our approach with Jeffrey and Rathke’s [13]. Moreover, we offer extended discussions of related works.

## Overview: characteristic bisimulations

We explain how we exploit session types to define characteristic bisimilarity. Key notions are *triggered* and *characteristic processes/values*. We first informally introduce some basic notation and terminology; formal definitions will be given in Sect. 3.

*Preliminaries* The syntax of $\textsf {HO}\pi$ considered in this paper is given below. We write *n* to range over shared names $a,b,\ldots$ and $s, {s}', \ldots$ to range over session (linear) names. Also, *u*, *w* denotes a name or a name variable. Session names are sometimes called *endpoints*. We consider a notion of *duality* on names, particularly relevant for session names: we shall write $\overline{s}$ to denote the dual endpoint of *s*.

$$\begin{aligned} \begin{array}{rcll} \text {Values}~~ V,W &{} \;\;{:}{:}{=}\;\;&{} u &{} \text {names (shared and linear)} \\ &{} \;\;\;|\;\;\;&{} \lambda x.\,P &{} \text {abstractions} \\ \text {Processes}~~ P,Q &{} \;\;{:}{:}{=}\;\;&{} u !\langle V \rangle .{P} \;\;\;|\;\;\;u ?(x) .{P} &{} \text {output and input} \\ &{} \;\;\;|\;\;\;&{} u \triangleleft l . P \;\;\;|\;\;\;u \triangleright \{l_i:P_i\}_{i \in I}~~ &{} \text {labelled choice} \\ &{} \;\;\;|\;\;\;&{} X \;\;\;|\;\;\;\mu X. P &{} \text {recursion} \\ &{} \;\;\;|\;\;\;&{} V\, {W} &{} \text {value application} \\ &{} \;\;\;|\;\;\;&{} P\;|\;Q \;\;\;|\;\;\;(\nu \, n) P \;\;\;|\;\;\;\mathbf {0}&{} \text {composition, restriction, inaction} \end{array} \end{aligned}$$

Hence, the higher-order character of $\textsf {HO}\pi$ comes from the fact that values exchanged in synchronisations include abstractions.

The semantics of $\textsf {HO}\pi$ can be given in terms of a labelled transition system (LTS), denoted $P \xrightarrow {\ell } P'$, where $\ell$ denotes a transition label or the internal action $\tau$. This way, for instance, $P \xrightarrow {n ?\langle V \rangle } P'$ denotes an input transition (a value *V* received along *n*) and $P \xrightarrow {(\nu \, \widetilde{m}) n !\langle V \rangle } P'$ denotes an output transition (a value *V* emitted along *n*, extruding names $\widetilde{m}$). Weak transitions, written $P \mathop {\Longrightarrow }\limits ^{\ell } P'$, abstract from internal actions in the usual way. Throughout the paper, we write $\mathfrak {R}, \mathfrak {R}',\ldots$ to denote binary relations on (typed) processes.

$\textsf {HO}\pi$ processes specify structured communications (protocols) as disciplined by *session types*, denoted $S, S', \ldots$, which we informally describe next:

$$ \begin{aligned} \begin{array}{lcll} S &{} \;\;{:}{:}{=}\;\;&{} !\langle U \rangle ; S \;\;\;|\;\;\; ?(U) ; S &{} \hbox {output/input value of type }\, U \hbox {, continue as } S \\ &{}\;\;\;|\;\;\;&{} \oplus \{l_i:S_i\}_{i \in I} \;\;\;|\;\;\; { \& } \{l_i:S_i\}_{i \in I}~ &{} \hbox {internal/external labelled choice of an}\ S_i \\ &{} \;\;\;|\;\;\;&{} \mu \textsf {t}.S \;\;\;|\;\;\;\textsf {t} &{} \text {recursive protocol} \\ &{}\;\;\;|\;\;\;&{} \texttt {end}&{} \text {completed protocol} \end{array} \end{aligned}$$

As we will see, type *U* denotes first-order values (i.e., shared and session names) but also shared and linear functional types, denoted $U\!\! \rightarrow \! \diamond$ and $U\!\! \multimap \! \diamond$, respectively, where $\diamond$ is the type for processes.

*Issues of context bisimilarity* Context bisimilarity (denoted $\approx$, cf. Definition 12) is an overly demanding relation on higher-order processes. It is far from satisfactory due to two issues, associated to demanding clauses for output and input actions. A *first issue* is the universal quantification in the output clause of context bisimilarity. Suppose $P \,\mathfrak {R}\, Q$, for some context bisimulation $\mathfrak {R}$. We have the following clause:

- $(\star )$ Whenever $P \xrightarrow {(\nu \, \widetilde{m_1}) n !\langle V \rangle } P'$ there exist $Q'$, *W* such that $Q \mathop {\Longrightarrow }\limits ^{(\nu \, \widetilde{m_2}) n !\langle W \rangle } Q'$ and,
***for all*** *R* with $\texttt {fv}(R)=\{x\}$, $(\nu \, \widetilde{m_1})(P' \;|\;RV/x) \,\mathfrak {R}\, (\nu \, \widetilde{m_2})(Q' \;|\;RW/x)$.

Intuitively, process *R* above stands for any possible *context* to which the emitted value (*V* and *W*) is supposed to go. (As usual, $RV/x$ denotes the capture-avoiding substitution of *V* for *x* in process *R*.) As explained in [31], considering all possible contexts *R* is key to achieve an adequate distinguishing power.

The *second issue* is due to inputs, and follows from the fact that we work with an *early* labelled transition system (LTS). Thus, an input prefix may observe infinitely many different values.

To alleviate these issues, in *characteristic bisimilarity* (denoted $\approx ^\mathtt{C}$, cf. Definition 18) we take two (related) steps:

- We replace $(\star )$ with a clause involving a context *more tractable* than $RV/x$ (and $RW/x$); and
- We refine inputs to avoid observing infinitely many actions on the same input prefix.

*Trigger processes* To address (a), we exploit session types. We first observe that, for any *V*, process $RV/x$ in $(\star )$ is context bisimilar to the process

$$\begin{aligned} P = (\nu \, s)((\lambda z.\,z ?(x) .{R})\, {s} \;|\;\overline{s} !\langle V \rangle . \mathbf {0}) \end{aligned}$$

In fact, through a name application and a synchronisation on session endpoint *s* we do have $P \approx RV/x$:where it is worth noticing that application and endpoint synchronisations are deterministic.

Now let us consider process $T_{V}$ below, where *t* is a fresh name:

1 $$\begin{aligned} T_{V} = t ?(x) . (\nu \, s)(x\, {s} \;|\;\overline{s} !\langle V \rangle . \mathbf {0}) \end{aligned}$$

If $T_{V}$ inputs value $\lambda z.\,z ?(x) . R$ then we have:

$$\begin{aligned} T_{V} \xrightarrow {t ?\langle \lambda z.\,z ?(x) . R \rangle } RV/x \approx P \end{aligned}$$

Processes such as $T_{V}$ offer a value at a fresh name; this class of ***trigger processes*** already suggests a tractable formulation of bisimilarity without the demanding output clause $(\star )$. Process $T_{V}$ in (1) requires a higher-order communication along *t*. As we explain below, we can give an alternative trigger process; the key is using *elementary inhabitants* of session types.

*Characteristic processes and values* To address (b), we limit the possible input values (such as $\lambda z.\,z ?(x) . R$ above) by exploiting session types. The key concept is that of ***characteristic process/value*** of a type, i.e., a simple process term that inhabits that type (Definition 13). To illustrate the key idea underlying characteristic processes, consider the session type

$$\begin{aligned} S = ?(S_1\!\! \rightarrow \! \diamond ) ; !\langle S_2 \rangle ; \texttt {end}~, \end{aligned}$$

which abstracts a protocol that first inputs an abstraction (i.e., a function from values $S_1$ to processes), and then outputs a value of type $S_2$. Let *P* be the process $u ?(x) . (u !\langle s_2 \rangle . \mathbf {0}\;|\;x\, {s_1})$, where $s_1, s_2$ are fresh names. It can be shown that *P* inhabits session type *S*; for the purposes of the behavioural theory developed in this paper, process *P* will serve as a kind of characteristic (representative) process for *S* along name *u*.

Given a session type *S* and a name *u*, we write $[\!\!(S)\!\!]^{u}$ for the characteristic process of *S* along *u*. Also, given a value type *U* (i.e., a type for channels or abstractions), we write $[\!\!(U)\!\!]_{\textsf {c}}$ to denote its *characteristic value* (cf. Definition 13). As we explain next, we use $[\!\!(U)\!\!]_{\textsf {c}}$ to refine input transitions.

*Refined input transitions* To refine input transitions, we need to observe an additional value, $\lambda {x}.\,t ?(y) . (y\, {{x}})$, called the ***trigger value*** (cf. Definition 14). This is necessary: it turns out that a characteristic value alone as the observable input is not enough to define a sound bisimulation (cf. Example 5). Intuitively, the trigger value is used to observe/simulate application processes.

Based on the above discussion, we define an alternative LTS on typed processes, denoted . We use this refined LTS to define characteristic bisimulation (Definition 18), in which the demanding clause $(\star )$ is replaced with a more tractable output clause based on characteristic trigger processes (cf. (2) below). Key to this alternative LTS is the following (refined) transition rule for input actions (cf. Definition 15) which, roughly speaking, given some fresh *t*, only admits names *m*, trigger values $\lambda {x}.\,t ?(y) . (y\, {{x}})$, and characteristic values $[\!\!(U)\!\!]_{\textsf {c}}$:Note the different notation for standard and refined transitions: $\xrightarrow {n ?\langle V \rangle }$ vs. .

*Characteristic triggers* Following the same reasoning as (1), we can use an alternative trigger process, called ***characteristic trigger process***, to replace clause ($\star$). Given a fresh name *t* and a value *V* of with type *U*, we have:

2 $$\begin{aligned} {~~t \Leftarrow _{\texttt {C}} V{\,:\,}U \mathop {=}\limits ^{\texttt {def}\ }t ?(x) . (\nu \, s)(s ?(y) . [\!\!(U)\!\!]^{y} \;|\;\overline{s} !\langle V \rangle . \mathbf {0})~~} \end{aligned}$$

This formulation is justified, because given $T_V$ as in (1), we may show thatThus, unlike process (1), the characteristic trigger process in (2) does not involve a higher-order communication on *t*. In contrast to previous approaches [13, 30] our characteristic trigger processes do *not* use recursion or replication. This is key to preserve linearity of session endpoints.

It is also noteworthy that $\textsf {HO}\pi$ lacks name matching, which is crucial in [13] to prove completeness of bisimilarity. The lack of matching operators is compensated here with the use of (session) types. Matching gives the observer the ability to test the equality of received names. In contrast, in our theory a process trigger embeds a name into a characteristic process so as to observe its (typed) behaviour. Thus, equivalent processes deal with (possibly different) names that have the same (typed) behaviour.

## A higher-order session $\pi$-calculus

We introduce the *higher-order session* $\pi$-*calculus* ($\textsf {HO}\pi$) which, as hinted at above, includes both name and abstraction passing, shared and session communication, as well as recursion; it is essentially the language proposed in [25], where a behavioural theory is not developed.

**Fig. 1:**
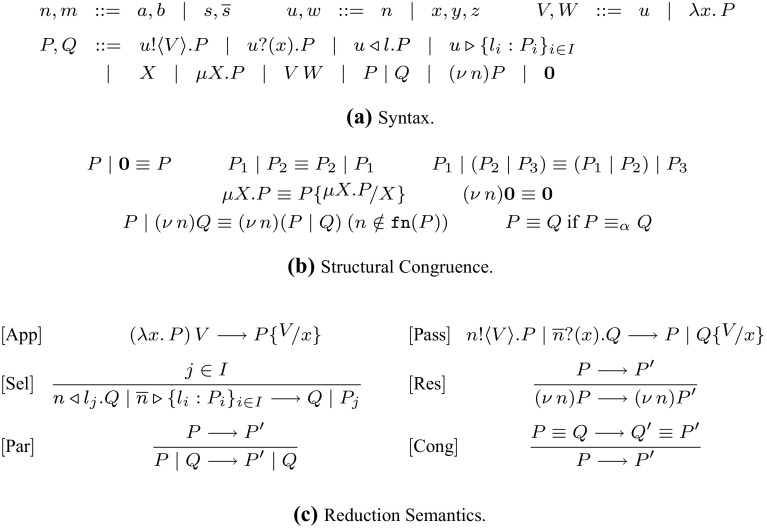
$\textsf {HO}\pi$: syntax and semantics (structural congruence and reduction)

### Syntax

The syntax of $\textsf {HO}\pi$ is defined in Fig. 1a. We use $a,b,c, \dots$ to range over shared names and $s, \overline{s}, \dots$ to range over session names. We use $n, m, t, \dots$ for session or shared names. Intuitively, session names represent deterministic communication *endpoints*, while shared names represent non-deterministic points. We define the dual operation over names *n* as $\overline{n}$ with $\overline{\overline{s}} = s$ and $\overline{a} = a$. This way, e.g., session names *s* and $\overline{s}$ are two dual endpoints. Name variables are denoted with $x, y, z, \dots$, and recursive variables are denoted with $X, Y, \dots$. Values *V*, *W* include name identifiers $u, v, \ldots$ (first-order values) and abstractions $\lambda x.\,P$ (higher-order values), where *P* is a process *P* and *x* is a name parameter.

Process terms include usual $\pi$-calculus constructs for sending and receiving values *V*: process $u !\langle V \rangle . P$ denotes the output of *V* over name *u*, with continuation *P*, while process $u ?(x) . P$ denotes the input prefix on name *u* of a value that will substitute variable *x* in the continuation *P*. Recursion is expressed by $\mu X. P$, which binds the recursive variable $X$ in process *P*. Process $V\, {W}$ represents the application of abstraction *V* to value *W*. Typing ensures that *V* is not a name. In the spirit of session-based $\pi$-calculi [9], we consider processes $u \triangleright \{l_i: P_i\}_{i \in I}$ and $u \triangleleft l . P$ to define labelled choice: given a finite index set *I*, process $u \triangleright \{l_i: P_i\}_{i \in I}$ offers a choice among processes with pairwise distinct labels; process $u \triangleleft l . P$ selects label *l* on name *u* and then behaves as *P*. Constructs for inaction $\mathbf {0}$ and parallel composition $P_1 \;|\;P_2$ are standard. Name restriction $(\nu \, n) P$ is also as customary; we notice that restriction for session names $(\nu \, s) P$ simultaneously binds endpoints *s* and $\overline{s}$ in *P*.

We use $\texttt {fv}(P)$ and $\texttt {fn}(P)$ to denote the sets of free variables and names in *P*, respectively. In a statement, we will say that a name is *fresh* if it is not among the names of the objects (processes, actions, etc.) of the statement. We assume that *V* in $u !\langle V \rangle .{P}$ does not include free recursive variables $X$. If $\texttt {fv}(P) = \emptyset$, we call *P* *closed*.

### Semantics

Figure 1c defines the operational semantics of $\textsf {HO}\pi$, given as a reduction relation that relies on a *structural congruence* relation, denoted $\equiv$ (Fig. 1b): it includes a congruence that ensures the consistent renaming of bound names, denoted $\equiv _\alpha$. We assume the expected extension of $\equiv$ to values *V*. Reduction is denoted $\longrightarrow$; some intuitions on the rules in Fig. 1 follow. Rule ${{[\text {App}]}}$ defines value application. Rule ${{[\text {Pass}]}}$ defines an interaction/synchronization at *n*; it can be on a shared name (with $\overline{n}=n$) or a session endpoint. Rule ${{[\text {Sel}]}}$ is the standard rule for labelled choice/selection [9]: given a finite index set *I*, a process selects label $l_j$ on name *n* over a pairwise distinct set of labels $\{l_i\}_{i \in I}$ offered by a branching on the dual endpoint $\overline{n}$; as a result, process $P_j$ is selected, and the remaining alternatives are discarded. Other rules are standard. We write $\longrightarrow ^*$ for a multi-step reduction.

### An example: the hotel booking scenario

To illustrate $\textsf {HO}\pi$ and its expressive power, let us consider a usecase scenario that adapts the example given by Mostrous and Yoshida [25, 26]. The scenario involves a $\textsf {Client}$ process that wants to book a hotel room. $\textsf {Client}$ narrows the choice down to two hotels, and requires a quote from the two in order to decide. The round-trip time (RTT) required for taking quotes from the two hotels in not optimal, so the client sends mobile processes to both hotels to automatically negotiate and book a room.

We now present two $\textsf {HO}\pi$ implementations of this scenario. For convenience, we write $\texttt {if}\ e\ \texttt {then}\ (P_1\ \varvec{;} \ P_2)$ to denote a conditional process that executes $P_1$ or $P_2$ depending on boolean expression *e* (encodable using labelled choice). The *first implementation* is as follows:

$$\begin{aligned}&\textsf {Client}_1 \mathop {=}\limits ^{\texttt {def}\ }(\nu \, h_1, h_2)(s_1 !\langle \lambda x.\,P_{xy} h_1/y \rangle . s_2 !\langle \lambda x.\,P_{xy} h_2/y \rangle . \mathbf {0}\;|\;\\&\quad \overline{h_1} ?(x) . \overline{h_2} ?(y) . \texttt {if}\ x \le y\ \texttt {then}\ \\&\quad \qquad (\overline{h_1} \triangleleft \textsf {accept} . \overline{h_2} \triangleleft \textsf {reject} . \mathbf {0}\ \varvec{;} \ \overline{h_1} \triangleleft \textsf {reject} . \overline{h_2} \triangleleft \textsf {accept} . \mathbf {0}) )\\&\quad P_{xy} \mathop {=}\limits ^{\texttt {def}\ }x !\langle \textsf {room} \rangle . x ?(\textsf {quote}) . y !\langle \textsf {quote} \rangle . y \triangleright \left\{ \begin{array}{l} \textsf {accept}: x \triangleleft \textsf {accept} . x !\langle \textsf {credit} \rangle . \mathbf {0}~,\\ \textsf {reject}: x \triangleleft \textsf {reject} . \mathbf {0}\end{array} \right\} \end{aligned}$$

Process $\textsf {Client}_1$ sends two abstractions with body $P_{xy}$, one to each hotel, using sessions $s_1$ and $s_2$. That is, $P_{xy}$ is the mobile code with free names *x*, *y*: while name *x* is meant to be instantiated by the hotel as the negotiating endpoint, name *y* is used to interact with $\textsf {Client}_1$. Intuitively, process $P_{xy}$:

(i) sends the room requirements to the hotel;
(ii) receives a quote from the hotel;
(iii) sends the quote to $\textsf {Client}_1$;
(iv) expects a choice from $\textsf {Client}_1$ whether to accept or reject the offer;
(v) if the choice is $\textsf {accept}$ then it informs the hotel and performs the booking; otherwise, if the choice is $\textsf {reject}$ then it informs the hotel and ends the session.

$\textsf {Client}_1$ instantiates two copies of $P_{xy}$ as abstractions on session *x*. It uses two fresh endpoints $h_1, h_2$ to substitute channel *y* in $P_{xy}$. This enables communication with the mobile code(s). In fact, $\textsf {Client}_1$ uses the dual endpoints $\overline{h_1}$ and $\overline{h_2}$ to receive the negotiation result from the two remote instances of *P* and then inform the two processes for the final booking decision.

We present now a *second implementation* in which the two mobile processes reach an agreement by interacting with each other (rather than with the client):

$$\begin{aligned} \begin{array}{rcl} \textsf {Client}_2 &{}\mathop {=}\limits ^{\texttt {def}\ }&{} (\nu \, h)(s_1 !\langle \lambda x.\,Q_1 h/y \rangle . s_2 !\langle \lambda x.\,Q_2 \overline{h}/y \rangle . \mathbf {0}) \\ Q_1 &{}\mathop {=}\limits ^{\texttt {def}\ }&{} x !\langle \textsf {room} \rangle . x ?(\textsf {quote}_1) . y !\langle \textsf {quote}_1 \rangle . y ?(\textsf {quote}_2) . R_x \\ Q_2 &{}\mathop {=}\limits ^{\texttt {def}\ }&{} x !\langle \textsf {room} \rangle . x ?(\textsf {quote}_1) . y ?(\textsf {quote}_2) . y !\langle \textsf {quote}_1 \rangle . R_x \\ R_x &{} \mathop {=}\limits ^{\texttt {def}\ }&{} \texttt {if}\ \ \textsf {quote}_1 \le \textsf {quote}_2 \, \texttt {then}\ (x \triangleleft \textsf {accept} . x !\langle \textsf {credit} \rangle . \mathbf {0}\ \varvec{;} \ x \triangleleft \textsf {reject} . \mathbf {0}) \end{array} \end{aligned}$$

Processes $Q_1$ and $Q_2$ negotiate a quote from the hotel in the same fashion as process $P_{xy}$ in $\textsf {Client}_1$. The key difference with respect to $P_{xy}$ is that *y* is used for interaction between process $Q_1$ and $Q_2$. Both processes send their quotes to each other and then internally follow the same logic to reach to a decision. Process $\textsf {Client}_2$ then uses sessions $s_1$ and $s_2$ to send the two instances of $Q_1$ and $Q_2$ to the two hotels, using them as abstractions on name *x*. It further substitutes the two endpoints of a fresh channel *h* to channels *y* respectively, in order for the two instances to communicate with each other.

**Fig. 2:**
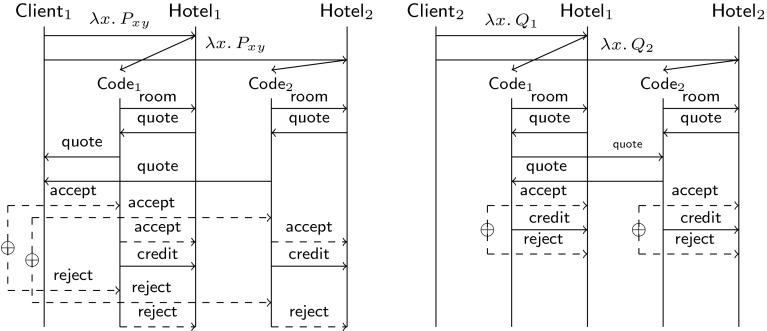
Sequence diagrams for $\textsf {Client}_1$ and $\textsf {Client}_2$, as in Sect. 3.3

The different protocols implemented by $\textsf {Client}_1$ and $\textsf {Client}_2$ can be represented by the sequence diagrams of Fig. 2. We will assign session types to these processes in Example 1. Later on, in Sect. 5.9 we will show that $\textsf {Client}_1$ and $\textsf {Client}_2$ are behaviourally equivalent using characteristic bisimilarity; see Proposition 3.

## Types and typing

We define a session typing system for $\textsf {HO}\pi$ and state its main properties. As we explain below, our system distils the key features of [25, 26].

### Types

The syntax of types of $\textsf {HO}\pi$ is given below:

$$ \begin{aligned} \begin{array}{cc} \begin{array}{lcl} \text {(value)} &{} U \;\;{:}{:}{=}\;\;&{} C \;\;\;|\;\;\;L \\ \text {(name)} &{} C \;\;{:}{:}{=}\;\;&{} S \;\;\;|\;\;\;\langle S \rangle \;\;\;|\;\;\;\langle L \rangle \\ \text {(abstractions)}~~ &{} L \;\;{:}{:}{=}\;\;&{} U\!\! \rightarrow \! \diamond \;\;\;|\;\;\;U\!\! \multimap \! \diamond \\ \text {(session)} &{} S \;\;{:}{:}{=}\;\;&{} !\langle U \rangle ; S \;\;\;|\;\;\;?(U) ; S \;\;\;|\;\;\;\oplus \{l_i:S_i\}_{i \in I} \;\;\;|\;\;\;{ \& } \{l_i:S_i\}_{i \in I} \\ &{} \;\;\;|\;\;\;&{} \mu \textsf {t}.S \;\;\;|\;\;\;\textsf {t} \;\;\;|\;\;\;\texttt {end}\end{array} \end{array} \end{aligned}$$

Value types *U* include the first-order types *C* and the higher-order types *L*. Session types are denoted with *S* and shared types with $\langle S \rangle$ and $\langle L \rangle$. We write $\diamond$ to denote the *process type*. The functional types $U\!\! \rightarrow \! \diamond$ and $U\!\! \multimap \! \diamond$ denote *shared* and *linear* higher-order types, respectively. Session types have the meaning already motivated in Sect. 2. The *output type* $!\langle U \rangle ; S$ first sends a value of type *U* and then follows the type described by *S*. Dually, $?(U) ; S$ denotes an *input type*. The *selection type* $\oplus \{l_i:S_i\}_{i \in I}$ and the *branching type* ${ \& } \{l_i:S_i\}_{i \in I}$ define labelled choice, implemented at the level of processes by internal and external choice mechanisms, respectively. Type $\texttt {end}$ is the termination type. We assume the *recursive type* $\mu \textsf {t}.S$ is guarded, i.e., the type variable $\textsf {t}$ only appears under prefixes. This way, e.g., the type $\mu \textsf {t}.\textsf {t}$ is not allowed. The sets of free/bound variables of a session type *S* are defined as usual; the sole binder is $\mu \textsf {t}.S$. Closed session types do not have free type variables.

Our type system is strictly included in that considered in [25, 26], which admits asynchronous communication and arbitrary nesting in functional types, i.e., their types are of the form $U \multimap T$ (resp. $U \rightarrow T$), where *T* ranges over *U* and the process type $\diamond$. In contrast, our functional types are of the form $U\!\! \multimap \! \diamond$ (resp. $U\!\! \rightarrow \! \diamond$).

We rely on notions of *duality* and *equivalence* for types. Let us write $S_1 \sim S_2$ to denote that $S_1$ and $S_2$ are *type-equivalent* (see Definition 21 in the Appendix). This notion extends to value types as expected; in the following, we write $U_1 \sim U_2$ to denote that $U_1$ and $U_2$ are type-equivalent. We write $S_1 \ \textsf {dual}\ S_2$ if $S_1$ is the *dual* of $S_2$. Intuitively, duality converts ! into ? and $\oplus$ into & (and vice-versa). More formally, following [4], we have a co-inductive definition for type duality:

#### Definition 1

(*Duality*) Let ${\mathsf {S}}{\mathsf {T}}$ be a set of closed session types. Two types *S* and $S'$ are said to be *dual* if the pair $(S,S')$ is in the largest fixed point of the monotone function $F:{\mathcal {P}}({\mathsf {S}}{\mathsf {T}}\times {\mathsf {S}}{\mathsf {T}}) \rightarrow {\mathcal {P}}({\mathsf {S}}{\mathsf {T}}\times {\mathsf {S}}{\mathsf {T}})$ defined by:

$$ \begin{aligned} F(\mathfrak {R})= & {} \{(\texttt {end}, \texttt {end})\}\\&\cup \{(!\langle U_1 \rangle ; S_1, ?(U_2) ; S_2) \;\;\;|\;\;\;(S_1, S_2)\in \mathfrak {R}, \ U_1 \sim U_2 \}\\&\cup \{(?(U_1) ; S_1, !\langle U_2 \rangle ; S_2) \;\;\;|\;\;\;(S_1, S_2)\in \mathfrak {R}, \ U_1 \sim U_2\}\\&\cup \{(\oplus \{l_i: S_i\}_{i \in I},\, { \& } \{l_i: S_i'\}_{i \in I}) \;\;\;|\;\;\;\forall i\in I. (S_i, S_i')\in \mathfrak {R}\}\\&\cup \{({ \& } \{l_i: S_i\}_{i \in I},\, \oplus \{l_i: S_i'\}_{i \in I}) \;\;\;|\;\;\;\forall i\in I. (S_i, S_i')\in \mathfrak {R}\}\\&\cup \{(\mu \textsf {t}.S, S') \;\;\;|\;\;\;(S\mu \textsf {t}.S/\textsf {t},S')\in \mathfrak {R}\}\\&\cup \{(S,\mu \textsf {t}.S') \;\;\;|\;\;\;(S,S'\mu \textsf {t}.S'/\textsf {t})\in \mathfrak {R}\}\\ \end{aligned}$$

Standard arguments ensure that *F* is monotone, thus the greatest fixed point of *F* exists. We write $S_1 \ \textsf {dual}\ S_2$ if $(S_1,S_2)\in \mathfrak {R}$.

### Typing environments and judgements

Typing *environments* are defined below:

$$\begin{aligned}&\varGamma \;\;{:}{:}{=}\;\;\emptyset \;\;\;|\;\;\;\varGamma \cdot x: U\!\! \rightarrow \! \diamond \;\;\;|\;\;\;\varGamma \cdot u: \langle S \rangle \;\;\;|\;\;\;\varGamma \cdot u: \langle L \rangle \;\;\;|\;\;\;\varGamma \cdot X: \varDelta \\&\varLambda \;\;{:}{:}{=}\;\;\emptyset \;\;\;|\;\;\;\varLambda \cdot x : U\!\! \multimap \! \diamond \\&\varDelta \;\;{:}{:}{=}\;\;\emptyset \;\;\;|\;\;\;\varDelta \cdot u : S \end{aligned}$$

Typing environments $\varGamma$, $\varLambda$, and $\varDelta$ satisfy different structural principles. Intuitively, the *exchange* principle indicates that the ordering of type assignments does not matter. *Weakening* says that type assignments need not be used. Finally, *contraction* says that type assignments may be duplicated.

The environment $\varGamma$ maps variables and shared names to value types, and recursive variables to session environments; it admits weakening, contraction, and exchange principles. While $\varLambda$ maps variables to linear higher-order types, $\varDelta$ maps session names to session types. Both $\varLambda$ and $\varDelta$ are only subject to exchange. The domains of $\varGamma , \varLambda$ and $\varDelta$ are assumed pairwise distinct.

Given $\varGamma$, we write $\varGamma \backslash x$ to denote the environment obtained from $\varGamma$ by removing the assignment $x:U\!\! \rightarrow \! \diamond$, for some *U*. This notation applies similarly to $\varDelta$ and $\varLambda$; we write $\varDelta \backslash \varDelta '$ (and $\varLambda \backslash \varLambda '$) with the expected meaning. Notation $\varDelta _1\cdot \varDelta _2$ means the disjoint union of $\varDelta _1$ and $\varDelta _2$. We define *typing judgements* for values *V* and processes *P*:

$$\begin{aligned} \varGamma ; \varLambda ; \varDelta \vdash V \triangleright U \qquad \qquad \qquad \qquad \qquad \varGamma ; \varLambda ; \varDelta \vdash P \triangleright \diamond \end{aligned}$$

While the judgement on the left says that under environments $\varGamma$, $\varLambda$, and $\varDelta$ value *V* has type *U*; the judgement on the right says that under environments $\varGamma$, $\varLambda$, and $\varDelta$ process *P* has the process type $\diamond$. The type soundness result for $\textsf {HO}\pi$ (Theorem 1) relies on two auxiliary notions on session environments:

#### Definition 2

(*Session environments: balanced/reduction*) Let $\varDelta$ be a session environment.

- $\varDelta$ is *balanced* if whenever $s: S_1, \overline{s}: S_2 \in \varDelta$ then $S_1 \ \textsf {dual}\ S_2$.
- We define the reduction relation $\longrightarrow$ on session environments as:
  $$ \begin{aligned} \varDelta \cdot s: !\langle U \rangle ; S_1 \cdot \overline{s}: ?(U) ; S_2\longrightarrow & {} \varDelta \cdot s: S_1 \cdot \overline{s}: S_2 \\ \varDelta \cdot s: \oplus \{l_i: S_i\}_{i \in I} \cdot \overline{s}: { \& } \{l_i: S_i'\}_{i \in I}\longrightarrow & {} \varDelta \cdot s: S_k \cdot \overline{s}: S_k' \ (k \in I) \end{aligned}$$

**Fig. 3:**
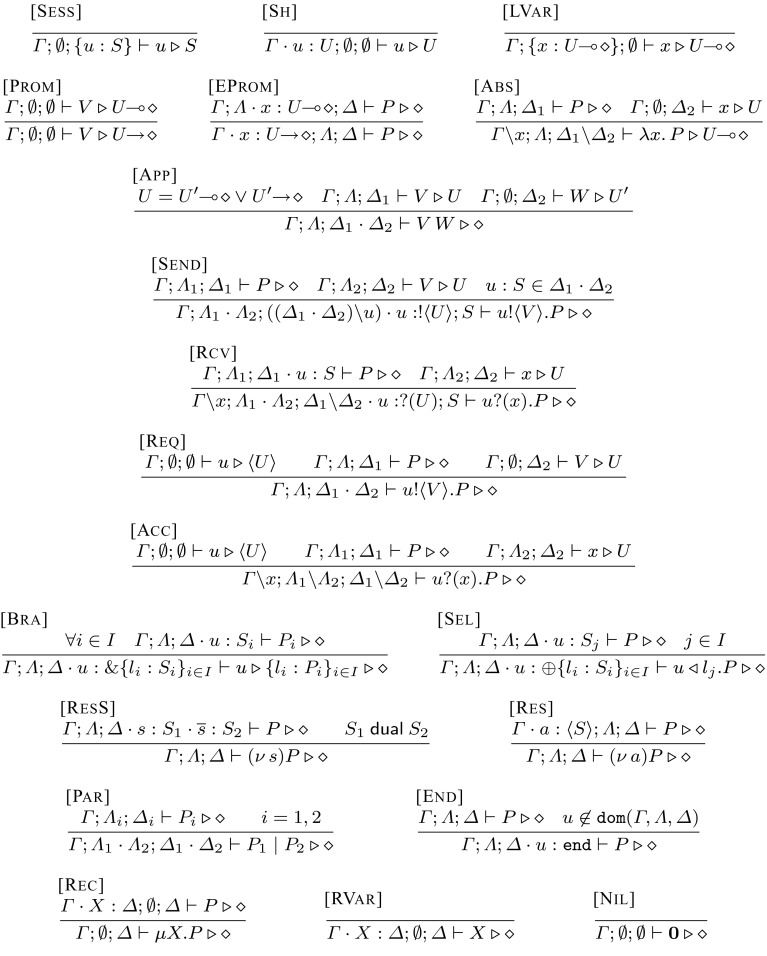
Typing rules for $\textsf {HO}\pi$

We rely on a typing system that is similar to the one developed in [25, 26]. The typing system is defined in Fig. 3. Rules $[{{\textsc {Sess}}]}$, $[{{\textsc {Sh}}]}$, $[{{\textsc {LVar}}]}$ are name and variable introduction rules. Rule ${[{{\textsc {Prom}}]}}$ allows a value with a linear type $U\!\! \multimap \! \diamond$ to be used as $U\!\! \rightarrow \! \diamond$ if its linear environment is empty. Rule ${[{{\textsc {EProm}}]}}$ allows to freely use a shared type variable in a linear way.

Abstraction values are typed with Rule ${[{{\textsc {Abs}}]}}$. The key type for an abstraction is the type for the bound variable of the abstraction, i.e., for a bound variable with type *C* the corresponding abstraction has type $C\!\! \multimap \! \diamond$. The dual of abstraction typing is application typing, governed by Rule ${[{{\textsc {App}}]}}$: we expect the type *U* of an application value *W* to match the type $U\!\! \multimap \! \diamond$ or $U\!\! \rightarrow \! \diamond$ of the application variable *x*.

In Rule ${[{{\textsc {Send}}]}}$, the type *U* of the sent value *V* should appear as a prefix on the session type $!\langle U \rangle ; S$ of *u*. Rule ${[{{\textsc {Rcv}}]}}$ is its dual. We use a similar approach with session prefixes to type interaction between shared names as defined in Rules ${[{{\textsc {Req}}]}}$ and ${[{{\textsc {Acc}}]}}$, where the type of the sent/received object (*S* and *L*, respectively) should match the type of the sent/received subject ($\langle S \rangle$ and $\langle L \rangle$, respectively). Rules ${[{{\textsc {Sel}}]}}$ and ${[{{\textsc {Bra}}]}}$ for selection and branching are standard: both rules prefix the session type with the selection type $\oplus \{l_i: S_i\}_{i \in I}$ and ${ \& } \{l_i:S_i\}_{i \in I}$, respectively.

A shared name creation *a* creates and restricts *a* in environment $\varGamma$ as defined in Rule $[{{\textsc {Res}}]}$. Creation of a session name *s* creates and restricts two endpoints with dual types in Rule $[{{\textsc {ResS}}]}$. Rule $[{{\textsc {Par}}]}$, combines the environments $\varLambda$ and $\varDelta$ of the parallel components of a parallel process. The disjointness of environments $\varLambda$ and $\varDelta$ is implied. Rule $[{{\textsc {End}}]}$ adds a name with type $\texttt {end}$ in $\varDelta$. The recursion requires that the body process matches the type of the recursive variable as in Rule $[{{\textsc {Rec}}]}$. The recursive variable is typed directly from the shared environment $\varGamma$ as in Rule  $[{{\textsc {RVar}}]}$. Rule $[{{\textsc {Nil}}]}$ says that the inactive process $\mathbf {0}$ is typed with empty linear environments $\varLambda$ and $\varDelta$.

We state the type soundness result for $\textsf {HO}\pi$ processes.

#### Theorem 1

(Type soundness) Suppose $\varGamma ; \emptyset ; \varDelta \vdash P \triangleright \diamond$ with $\varDelta$ balanced. Then $P \longrightarrow P'$ implies $\varGamma ; \emptyset ; \varDelta ' \vdash P' \triangleright \diamond$ and $\varDelta = \varDelta '$ or $\varDelta \longrightarrow \varDelta '$ with $\varDelta '$ balanced.

#### Proof

Following standard lines. See Appendix 1 for details. $\square$

#### Example 1

(The hotel booking example, revisited) We give types to the client processes of Sect. 3.3. Assume

$$ \begin{aligned} S= & {} !\langle \textsf {quote} \rangle ; { \& } \{\textsf {accept}: \texttt {end}, \textsf {reject}: \texttt {end}\} \\ U= & {} !\langle \textsf {room} \rangle ; ?(\textsf {quote}) ; \oplus \{\textsf {accept}: !\langle \textsf {credit} \rangle ; \texttt {end}, \textsf {reject}: \texttt {end}\} \end{aligned}$$

While the typing for $\lambda x.\,P_{xy}$ is $\emptyset ; \emptyset ; y: S \vdash \lambda x.\,P_{xy} \triangleright U\!\! \multimap \! \diamond$, the typing for $\textsf {Client}_1$ is $~~ \emptyset ; \emptyset ; s_1: !\langle U\!\! \multimap \! \diamond \rangle ; \texttt {end}\cdot s_2: !\langle U\!\! \multimap \! \diamond \rangle ; \texttt {end}\vdash \textsf {Client}_1 \triangleright \diamond$.

The typings for $Q_1$ and $Q_2$ are $\emptyset ; \emptyset ; y: !\langle \textsf {quote} \rangle ; ?(\textsf {quote}) ; \texttt {end}\vdash \lambda x.\,Q_i \triangleright U\!\! \multimap \! \diamond$ ($i=1,2$) and the type for $\textsf {Client}_2$ is $~~ \emptyset ; \emptyset ; s_1: !\langle U\!\! \multimap \! \diamond \rangle ; \texttt {end}\cdot s_2: !\langle U\!\! \multimap \! \diamond \rangle ; \texttt {end}\vdash \textsf {Client}_2 \triangleright \diamond$.

## Characteristic bisimulation

We develop a theory for observational equivalence over session typed $\textsf {HO}\pi$ processes that follows the principles laid in our previous works [18, 19]. We introduce *higher-order bisimulation* (Definition 17) and *characteristic bisimulation* (Definition 18), denoted $\approx ^\mathtt{H}$ and $\approx ^\mathtt{C}$, respectively. We prove that they coincide with (reduction-closed) barbed congruence (denoted $\cong$, cf. Definition 11), the form of contextual equivalence used in concurrency. This characterisation result is given in Theorem 2.

We briefly summarise our strategy for obtaining Theorem 2. We begin by defining an (early) labelled transition system (LTS) on untyped processes (Sect. 5.1). Then, using the *environmental* transition semantics (Sect. 5.2), we define a typed LTS that formalises how a typed process interacts with a typed observer. Later, we define reduction-closed, barbed congruence and context bisimilarity, respectively (Sects. 5.3 and 5.4). Subsequently, we define the refined LTS based on characteristic values (Sect. 5.5). Building upon this LTS, we define higher-order and characteristic bisimilarities (Sect. 5.6). Then, we develop an auxiliary proof technique based on deterministic transitions (Sect. 5.7). Our main result, the characterisation of barbed congruence in terms of $\approx ^\mathtt{H}$ and $\approx ^\mathtt{C}$, is stated in Sect. 5.8. Finally, we revisit our two implementations for the Hotel Booking Scenario (Sect. 3.3), using Theorem 2 to show that they are behaviourally equivalent (Sect. 5.9).

### Labelled transition system for processes

We define the interaction of processes with their environment using action labels $\ell$:

$$ \begin{aligned} \ell \;\;{:}{:}{=}\;\;\tau \;\;\;|\;\;\;(\nu \, \widetilde{m}) n !\langle V \rangle \;\;\;|\;\;\;n ?\langle V \rangle \;\;\;|\;\;\;n \oplus l \;\;\;|\;\;\;n \, \& \, l \end{aligned}$$

Label $\tau$ defines internal actions. Action $(\nu \, \widetilde{m}) n !\langle V \rangle$ denotes the sending of value *V* over channel *n* with a possible empty set of restricted names $\widetilde{m}$ (we may write $n !\langle V \rangle$ when $\widetilde{m}$ is empty). Dually, the action for value reception is $n ?\langle V \rangle$. Actions for select and branch on a label *l* are denoted $n \oplus l$ and $n \, \& \, l$, respectively. We write $\texttt {fn}(\ell )$ and $\texttt {bn}(\ell )$ to denote the sets of free/bound names in $\ell$, respectively. Given $\ell \ne \tau$, we say $\ell$ is a *visible action*; we write $\texttt {subj}(\ell )$ to denote its *subject*. This way, we have: $\texttt {subj}((\nu \, \widetilde{m}) n !\langle V \rangle ) = \texttt {subj}(n ?\langle V \rangle ) = \texttt {subj}(n \oplus l) = \texttt {subj}(n \, \& \, l) = n$.

**Fig. 4:**
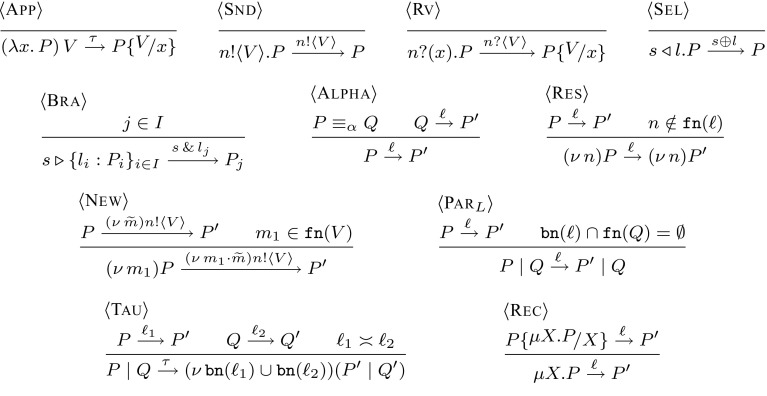
The untyped LTS for $\textsf {HO}\pi$ processes. We omit Rule $\langle {\textsc {Par}_{R}}\rangle$

*Dual actions* occur on subjects that are dual between them and carry the same object; thus, output is dual to input and selection is dual to branching.

#### Definition 3

(*Dual actions*) We define duality on actions as the least symmetric relation $\asymp$ on action labels that satisfies:

$$ \begin{aligned} n \oplus l \asymp \overline{n} \, \& \, l \qquad \qquad (\nu \, \widetilde{m}) n !\langle V \rangle \asymp \overline{n} ?\langle V \rangle \end{aligned}$$

The (early) labelled transition system (LTS) fpr *untyped* processes is given in Fig. 4. We write $P_1 \xrightarrow {\ell } P_2$ with the usual meaning. The rules are standard [18, 19]; we comment on some of them. A process with an output prefix can interact with the environment with an output action that carries a value *V* (Rule ${\langle \textsc {Snd} \rangle }$). Dually, in Rule ${\langle \textsc {Rv} \rangle }$ a receiver process can observe an input of an arbitrary value *V*. Select and branch processes observe the select and branch actions in Rules ${\langle \textsc {Sel} \rangle }$ and ${\langle \textsc {Bra} \rangle }$, respectively. Rule ${\langle \textsc {Res} \rangle }$ enables an observable action from a process with an outermost restriction, provided that the restricted name does not occur free in the action. If a restricted name occurs free in the carried value of an output action, the process performs scope opening (Rule ${\langle \textsc {New} \rangle }$). Rule ${\langle \textsc {Rec} \rangle }$ handles recursion unfolding. Rule ${\langle \textsc {Tau} \rangle }$ states that two parallel processes which perform dual actions can synchronise by an internal transition. Rules 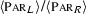 and ${\langle \textsc {Alpha} \rangle }$ define standard treatments for actions under parallel composition and $\alpha$-renaming.

### Environmental labelled transition system

**Fig. 5:**
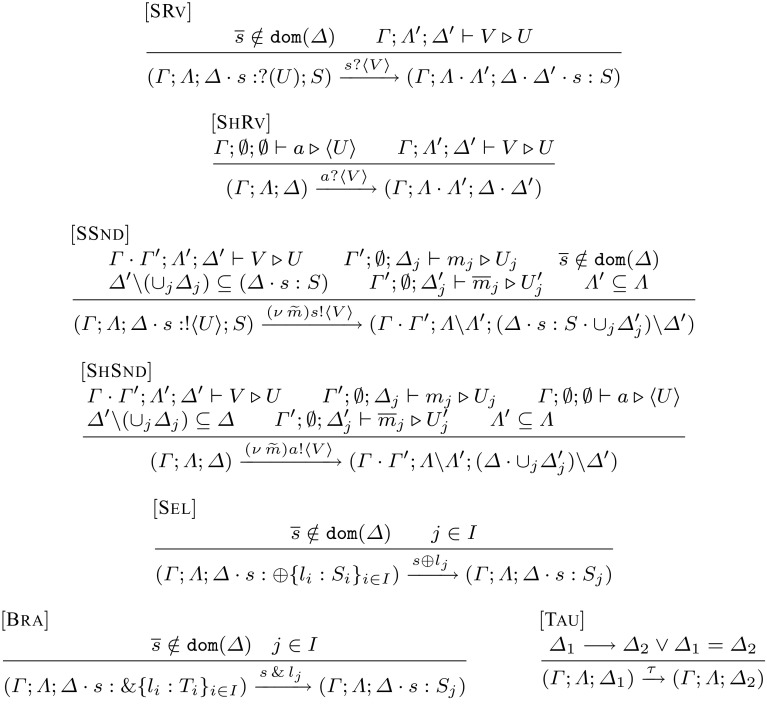
Labelled transition system for typed environments

Our typed LTS is obtained by coupling the untyped LTS given before with a labelled transition relation on typing environments, given in Fig. 5. Building upon the reduction relation for session environments in Definition 2, such a relation is defined on triples of environments by extending the LTSs in [18, 19]; it is denoted

$$\begin{aligned} (\varGamma _1, \varLambda _1, \varDelta _1) \xrightarrow {\ell } (\varGamma _2, \varLambda _2, \varDelta _2) \end{aligned}$$

Recall that $\varGamma$ admits weakening. Using this principle (not valid for $\varLambda$ and $\varDelta$), we have  whenever .

*Input actions* are defined by Rules ${[{\textsc {SRv}]}}$ and ${[{\textsc {ShRv}]}}$. In Rule ${[{\textsc {SRv}]}}$ the type of value *V* and the type of the object associated to the session type on *s* should coincide. The resulting type tuple must contain the environments associated to *V*. The dual endpoint $\overline{s}$ cannot be present in the session environment: if it were present the only possible communication would be the interaction between the two endpoints (cf. Rule ${[{\textsc {Tau}]}}$). Following similar principles, Rule ${[{\textsc {ShRv}]}}$ defines input actions for shared names.

*Output actions* are defined by Rules ${[{\textsc {SSnd}]}}$ and ${[{\textsc {ShSnd}]}}$. Rule ${[{\textsc {SSnd}]}}$ states the conditions for observing action $(\nu \, \widetilde{m}) s !\langle V \rangle$ on a type tuple $(\varGamma , \varLambda , \varDelta \cdot s : S)$. The session environment $\varDelta \,\cdot \, s : S$ should include the session environment of the sent value *V* (denoted $\varDelta '$ in the rule), *excluding* the session environments of names $m_j$ in $\widetilde{m}$ which restrict the scope of value *V* (denoted $\varDelta _j$ in the rule). Analogously, the linear variable environment $\varLambda '$ of *V* should be included in $\varLambda$. The rule defines the scope extrusion of session names in $\widetilde{m}$; consequently, environments associated to their dual endpoints (denoted $\varDelta '_j$ in the rule) appear in the resulting session environment. Similarly for shared names in $\widetilde{m}$ that are extruded. All free values used for typing *V* (denoted $\varLambda '$ and $\varDelta '$ in the rule) are subtracted from the resulting type tuple. The prefix of session *s* is consumed by the action. Rule ${[{\textsc {ShSnd}]}}$ follows similar ideas for output actions on shared names: the name must be typed with $\langle U \rangle$; conditions on value *V* are identical to those on Rule ${[{\textsc {SSnd}]}}$.

*Other actions* Rules ${[{\textsc {Sel}]}}$ and ${[{\textsc {Bra}]}}$ describe actions for select and branch. Rule ${[{\textsc {Tau}]}}$ defines internal transitions: it reduces the session environment (cf. Definition 2) or keeps it unchanged.

We illustrate Rule ${[{\textsc {SSnd}]}}$ by means of an example:

#### Example 2

Consider environment tuple $(\varGamma ;\, \emptyset ;\, s: !\langle (!\langle S \rangle ; \texttt {end})\!\! \multimap \! \diamond \rangle ; \texttt {end}\cdot s': S)$ and typed value $V= \lambda x.\,x !\langle s' \rangle . m ?(z) . \mathbf {0}$ with

$$\begin{aligned} \varGamma ; \emptyset ; s': S \cdot m: ?(\texttt {end}) ; \texttt {end}\vdash V \, \triangleright \, (!\langle S \rangle ; \texttt {end})\!\! \multimap \! \diamond \end{aligned}$$

Then, by Rule ${[{\textsc {SSnd}]}}$, we can derive:

$$\begin{aligned} (\varGamma ; \emptyset ; s: !\langle (!\langle S \rangle ; \texttt {end})\!\! \multimap \! \diamond \rangle ; \texttt {end}\cdot s': S) \xrightarrow {(\nu \, m) s !\langle V \rangle } (\varGamma ; \emptyset ; s: \texttt {end}\cdot \overline{m}: !\langle \texttt {end} \rangle ; \texttt {end}) \end{aligned}$$

Observe how the protocol along *s* is partially consumed; also, the resulting session environment is extended with $\overline{m}$, the dual endpoint of the extruded name *m*.

#### Notation 4

Given a value *V* of type *U*, we sometimes annotate the output action $(\nu \, \widetilde{m}) n !\langle V \rangle$ with the type of *V* as $(\nu \, \widetilde{m}) n !\langle V : U \rangle$.

The typed LTS combines the LTSs in Figs. 4 and 5.

#### Definition 5

(*Typed transition system*) A *typed transition relation* is a typed relation $\varGamma ; \varDelta _1 \vdash P_1 \xrightarrow {\ell } \varDelta _2 \vdash P_2$ where:

1. $$P_1 \xrightarrow {\ell } P_2$$ and
2. $$(\varGamma , \emptyset , \varDelta _1) \xrightarrow {\ell } (\varGamma , \emptyset , \varDelta _2)$$ with $$\varGamma ; \emptyset ; \varDelta _i \vdash P_i \triangleright \diamond $$ ($$i=1,2$$).

We write $\mathop {\Longrightarrow }\limits ^{}$ for the reflexive and transitive closure of $\xrightarrow {}$, $\mathop {\Longrightarrow }\limits ^{\ell }$ for the transitions $\mathop {\Longrightarrow }\limits ^{}\xrightarrow {\ell }\mathop {\Longrightarrow }\limits ^{}$, and $\mathop {\Longrightarrow }\limits ^{\hat{\ell }}$ for $\mathop {\Longrightarrow }\limits ^{\ell }$ if $\ell \not = \tau$ otherwise $\mathop {\Longrightarrow }\limits ^{}$.

A typed transition relation requires type judgements with an empty $$\varLambda $$, i.e., an empty environment for linear higher-order types. Notice that for open process terms (i.e., with free variables), we can always apply Rule $$ {[{{EProm}]}}$$ (cf. Fig. 3) and obtain an empty $$\varLambda $$. As it will be clear below (cf. Definition 7), we will be working with closed process terms, i.e., processes without free variables.

### Reduction-closed, barbed congruence ($\cong$)

We now define *typed relations* and *contextual equivalence* (i.e., barbed congruence). To define typed relations, we first define *confluence* over session environments $\varDelta$. Recall that $\varDelta$ captures session communication, which is deterministic. The notion of confluence allows us to abstract away from alternative computation paths that may arise due to non-interfering reductions of session names.

#### Definition 6

(*Session environment confluence*) Two session environments $\varDelta _1$ and $\varDelta _2$ are *confluent*, denoted $\varDelta _1 \rightleftharpoons \varDelta _2$, if there exists a $\varDelta$ such that: i) $\varDelta _1 \longrightarrow ^*\varDelta$ and ii) $\varDelta _2 \longrightarrow ^*\varDelta$ (here we write $\longrightarrow ^*$ for the multi-step reduction in Definition 2).

We illustrate confluence by means of an example:

#### Example 3

(Session environment confluence) Consider the (balanced) session environments:

$$\begin{aligned} \varDelta _1= & {} \{s_1: T_1 \cdot s_2: ?(U_2) ; \texttt {end}\cdot \overline{s_2}: !\langle U_2 \rangle ; \texttt {end}\} \\ \varDelta _2= & {} \{s_1: T_1 \cdot s_2: !\langle U_1 \rangle ; ?(U_2) ; \texttt {end}\cdot \overline{s_2}: ?(U_1) ; !\langle U_2 \rangle ; \texttt {end}\} \end{aligned}$$

Following Definition 2, we have that $\varDelta _1 \longrightarrow \{s_1: T_1 \cdot s_2: \texttt {end}\cdot \overline{s_2}: \texttt {end}\}$ and $\varDelta _2 \longrightarrow \longrightarrow \{s_1: T_1 \cdot s_2: \texttt {end}\cdot \overline{s_2}: \texttt {end}\}$. Therefore, $\varDelta _1$ and $\varDelta _2$ are confluent. $\square$

Typed relations relate only closed processes whose session environments are balanced and confluent:

#### Definition 7

(*Typed relation*) We say that a binary relation over typing judgementsis a *typed relation* whenever:

1. $P_1$ and $P_2$ are closed;
2. $\varDelta _1$ and $\varDelta _2$ are balanced (cf. Definition 2); and
3. $$\varDelta _1 \rightleftharpoons \varDelta _2$$ (cf. Definition 6).

#### Notation 8

(Typed relations) We write

$$\begin{aligned} \varGamma ; \varDelta _1 \vdash P_1 \ \mathfrak {R}\ \varDelta _2 \vdash P_2 \end{aligned}$$

to denote the typed relation $\varGamma ; \emptyset ; \varDelta _1 \vdash P_1 \triangleright \diamond \ \mathfrak {R}\ \varGamma ; \emptyset ; \varDelta _2 \vdash P_2 \triangleright \diamond$.

Next we define *barbs* [24] with respect to types.

#### Definition 9

(*Barbs*) Let *P* be a closed process. We write

1.
  - $P \downarrow _{n}$ if $P \equiv (\nu \, \tilde{m})(n !\langle V \rangle . P_2 \;|\;P_3)$ or $P \equiv (\nu \, \tilde{m})(n \triangleleft l . P_2 \;|\;P_3)$, with $n \notin \tilde{m}$.
  - We write $P \Downarrow _{n}$ if $P \longrightarrow ^* \downarrow _{n}$.
2. Similarly, we write
  - $\varGamma ; \emptyset ; \varDelta \vdash P \downarrow _{n}$ if $\varGamma ; \emptyset ; \varDelta \vdash P \triangleright \diamond$ with $P \downarrow _{n}$ and $\overline{n} \notin \varDelta$.
  - We write $\varGamma ; \emptyset ; \varDelta \vdash P \Downarrow _{n}$ if $P \longrightarrow ^* P'$ and $\varGamma ; \emptyset ; \varDelta ' \vdash P' \downarrow _{n}$.

A barb $\downarrow _{n}$ is an observable on an output (resp. select) prefix with subject *n*; a weak barb $\Downarrow _{n}$ is a barb after zero or more reduction steps. Typed barbs $\downarrow _{n}$ (resp. $\Downarrow _{n}$) are observed on typed processes $\varGamma ; \emptyset ; \varDelta \vdash P \triangleright \diamond$. When *n* is a session name we require that its dual endpoint $\overline{n}$ is not present in the session environment $\varDelta$.

Notice that observing output barbs is enough to (indirectly) observe input actions. For instance, the process $P = n ?(x) . P'$ has an input barb on *n*; by composing *P* with $n !\langle m \rangle . succ !\langle \rangle . \mathbf {0}$ (with a fresh name $succ$) then one obtains a (weak) observation uniquely associated to the input along *n* in *P*.

To define a congruence relation, we introduce the family $\mathbb {C}$ of contexts:

#### Definition 10

(*Context*) Context $\mathbb {C}$ is defined over the syntax:

$$\begin{aligned}&\mathbb {C}{:}{:}{=} -\;\;\;|\;\;\;u !\langle V \rangle . \mathbb {C}\;\;\;|\;\;\;u ?(x) . \mathbb {C}\;\;\;|\;\;\;u !\langle \lambda x.\mathbb {C} \rangle . P \;\;\;|\;\;\;(\nu \, n) \mathbb {C}\;\;\;|\;\;\;(\lambda x.\mathbb {C})u \;\;\;|\;\;\;\mu X. \mathbb {C}\\&\quad \;\;\;|\;\;\;\mathbb {C}\;|\;P \;\;\;|\;\;\;P \;|\;\mathbb {C}\;\;\;|\;\;\;u \triangleleft l . \mathbb {C}\;\;\;|\;\;\;u \triangleright \{l_1:P_1,\cdots ,l_i:\mathbb {C},\cdots ,l_n:P_n\} \end{aligned}$$

Notation $\mathbb {C}[P]$ denotes the result of substituting the hole $-$ in $\mathbb {C}$ with process *P*.

The first behavioural relation that we define is reduction-closed, barbed congruence [10].

#### Definition 11

(*Reduction-closed, barbed congruence*) Typed relation

$$\begin{aligned} \varGamma ; \varDelta _1 \vdash P \ \mathfrak {R}\ \varDelta _2 \vdash Q \end{aligned}$$

is a *reduction-closed, barbed congruence* whenever:

1.
  - If $P \longrightarrow P'$ then there exist $\varDelta _1', Q', \varDelta _2'$ such that $Q \longrightarrow ^* Q'$ and $\varGamma ; \varDelta _1' \vdash P' \ \mathfrak {R}\ \varDelta _2' \vdash Q'$;
  - and the symmetric case;
2.
  - If $$\varGamma ;\varDelta _1 \vdash P \downarrow _{n}$$ then $$\varGamma ;\varDelta _2 \vdash Q \Downarrow _{n}$$;
  - and the symmetric case;
3. For all $\mathbb {C}$, there exist $\varDelta _1'',\varDelta _2''$ such that $\varGamma ; \varDelta _1'' \vdash \mathbb {C}[P] \ \mathfrak {R}\ \varDelta _2'' \vdash \mathbb {C}[Q]$.

The largest such relation is denoted with $\cong$.

### Context bisimilarity ($\approx$)

Following Sangiorgi [31], we now define the standard (weak) context bisimilarity.

#### Definition 12

(*Context bisimilarity*) A typed relation $\mathfrak {R}$ is *a context bisimulation* if for all $\varGamma ; \varDelta _1 \vdash P_1 \ \mathfrak {R}\ \varDelta _2 \vdash Q_1$,

1. Whenever $\varGamma ; \varDelta _1 \vdash P_1 \xrightarrow {(\nu \, \widetilde{m_1}) n !\langle V_1 \rangle } \varDelta _1' \vdash P_2$, there exist $Q_2$, $V_2$, $\varDelta '_2$ such that $\varGamma ; \varDelta _2 \vdash Q_1 \mathop {\Longrightarrow }\limits ^{(\nu \, \widetilde{m_2}) n !\langle V_2 \rangle } \varDelta _2' \vdash Q_2$ and for all *R* with $\texttt {fv}(R)=\{x\}$:
  $$\begin{aligned} \varGamma ; \varDelta _1'' \vdash (\nu \, \widetilde{m_1})(P_2 \;|\;RV_1/x) \ \mathfrak {R}\ \varDelta _2'' \vdash (\nu \, \widetilde{m_2})(Q_2 \;|\;RV_2/x); \end{aligned}$$
2. For all $\varGamma ; \varDelta _1 \vdash P_1 \xrightarrow {\ell } \varDelta _1' \vdash P_2$ such that $\ell$ is not an output, there exist $Q_2$, $\varDelta '_2$ such that $\varGamma ; \varDelta _2 \vdash Q_1 \mathop {\Longrightarrow }\limits ^{\hat{\ell }} \varDelta _2' \vdash Q_2$ and $\varGamma ; \varDelta _1' \vdash P_2 \ \mathfrak {R}\ \varDelta _2' \vdash Q_2$; and
3. The symmetric cases of 1 and 2.

The largest such bisimulation is called *context bisimilarity* and is denoted by $\approx$.

As suggested in Sect. 2, in the general case, context bisimilarity is an overly demanding relation on processes. Below we introduce *higher-order bisimulation* and *characteristic bisimulation*, which are meant to offer a *tractable* proof technique over session typed processes with first- and higher-order communication.

### Characteristic values and the refined LTS

We formalise the ideas given in Sect. 2, concerning characteristic processes/values and the refined LTS. We first define characteristic processes/values:

#### Definition 13

**Fig. 6:**
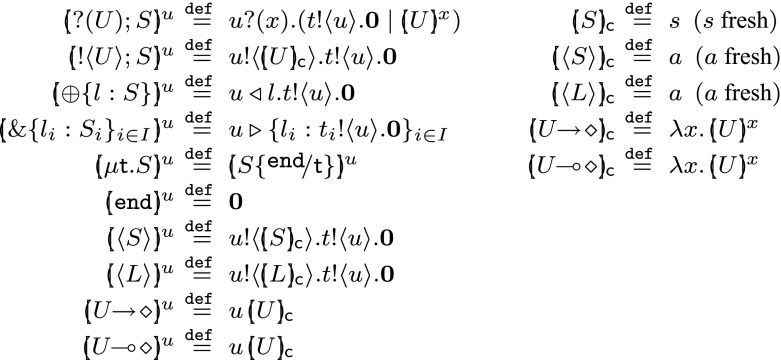
Characteristic processes (*left*) and characteristic values (*right*)

(*Characteristic process and values*) Let *u* and *U* be a name and a type, respectively. The *characteristic process* of *U* (along *u*), denoted $[\!\!(U)\!\!]^{u}$, and the *characteristic value* of *U*, denoted $[\!\!(U)\!\!]_{\textsf {c}}$, are defined in Fig. 6.

We can verify that characteristic processes/values do inhabit their associated type.

#### Proposition 1

(Characteristic processes/values inhabit their types)

1. Let *U* be a channel type. Then, for some $\varGamma , \varDelta$, we have $\varGamma ; \emptyset ; \varDelta \vdash [\!\!(U)\!\!]_{\textsf {c}} \triangleright U$.
2. Let *S* be a session type. Then, for some $\varGamma , \varDelta$, we have $\varGamma ; \emptyset ; \varDelta \cdot s: S \vdash [\!\!(S)\!\!]^{s} \triangleright \diamond$.
3. Let *U* be a channel type. Then, for some $\varGamma , \varDelta$, we have $\varGamma \cdot a: U; \emptyset ; \varDelta \vdash [\!\!(U)\!\!]^{a} \triangleright \diamond$.

#### Proof

(Sketch) The proof is done by induction on the syntax of types. See Proposition 4 in the Appendix for details. $\square$

We give an example of a characteristic process inhabiting a recursive type.

#### Example 4

(Characteristic process for a recursive session type) Consider the type $S = \mu \textsf {t}.!\langle U_1 \rangle ; ?(U_2) ; \textsf {t}$. By Definition 13, we have that $[\!\!(S)\!\!]^{s} = [\!\!(!\langle U_1 \rangle ; ?(U_2) ; \texttt {end})\!\!]^{s} = s !\langle [\!\!(U_1)\!\!]_{\textsf {c}} \rangle . t !\langle s \rangle . \mathbf {0}$. For this process, we can infer the following type derivations:

$$\begin{aligned} \frac{\varGamma ; \emptyset ; \varDelta \triangleright [\!\!(U_1)\!\!]_{\textsf {c}} \triangleright U_2 \varGamma ; \emptyset ; t: !\langle ?(U_2) ; \texttt {end} \rangle ; \texttt {end}\cdot s: ?(U_2) ; \texttt {end}\vdash t !\langle s \rangle . \mathbf {0}\triangleright \diamond }{ \varGamma ; \emptyset ; \varDelta \cdot t: !\langle ?(U_2) ; \texttt {end} \rangle ; \texttt {end}\cdot s: !\langle U_1 \rangle ; ?(U_2) ; \texttt {end}\vdash s !\langle [\!\!(U_1)\!\!]_{\textsf {c}} \rangle . t !\langle s \rangle . \mathbf {0}\triangleright \diamond } \end{aligned}$$

and

$$\begin{aligned} \frac{ \varGamma ; \emptyset ; \varDelta \cdot t: !\langle ?(U_2) ; \mu \textsf {t}.!\langle U_1 \rangle ; ?(U_2) ; \textsf {t} \rangle ; \texttt {end}\cdot s: ?(U_2) ; \mu \textsf {t}.!\langle U \rangle ; \textsf {t} \vdash t !\langle s \rangle . \mathbf {0}\triangleright \diamond }{ \varGamma ; \emptyset ; \varDelta \cdot t: !\langle ?(U_2) ; \mu \textsf {t}.!\langle U \rangle ; \textsf {t} \rangle ; \texttt {end}\cdot s: \mu \textsf {t}.!\langle U \rangle ; \textsf {t} \vdash s !\langle [\!\!(U_1)\!\!]_{\textsf {c}} \rangle . t !\langle s \rangle . \mathbf {0}\triangleright \diamond } \end{aligned}$$

The following example motivates the refined LTS explained in Sect. 2. We rely on the following definition.

#### Definition 14

(*Trigger value*) Given a fresh name *t*, the *trigger value* on *t* is defined as the abstraction $\lambda {x}.\,t ?(y) . (y\, {{x}})$.

#### Example 5

(The need for the refined typed LTS) We illustrate the complementary rôle that characteristic values (cf. Fig. 6) and the trigger value (Definition 14) play in defining sound bisimilarities.

We first notice that observing characteristic values as inputs is not enough to define a sound bisimulation. Consider processes

3 $$\begin{aligned} P_1 = s ?(x) . (x\, {s_1} \;|\;x\, {s_2})&\qquad \qquad&P_2 = s ?(x) . (x\, {s_1} \;|\;(\lambda z.\,\mathbf {0})\, {s_2}) \end{aligned}$$

such that

$$\begin{aligned} \varGamma ; \emptyset ; \varDelta \cdot s: ?((\texttt {end})\!\! \rightarrow \! \diamond ) ; \texttt {end}\vdash P_i \triangleright \diamond \qquad (i \in \{1,2\}) \end{aligned}$$

with $\varDelta = s_1{:}\texttt {end}\cdot s_2{:}\texttt {end}$. If $P_1$ and $P_2$ input along *s* a characteristic value of the form $[\!\!((\texttt {end})\!\! \rightarrow \! \diamond )\!\!]_{\textsf {c}} = \lambda z.\,\mathbf {0}$ (cf. Fig. 6), then both of them would evolve into:

$$\begin{aligned} \varGamma ; \emptyset ; \varDelta \vdash (\lambda z.\,\mathbf {0})\, {s_1} \;|\;(\lambda z.\,\mathbf {0})\, {s_2} \triangleright \diamond \end{aligned}$$

therefore becoming context bisimilar. However, processes $P_1$ and $P_2$ in (3) are clearly *not* context bisimilar: many input actions may be used to distinguish them. For example, if $P_1$ and $P_2$ input $\lambda x.\,(\nu \, s')(a !\langle s' \rangle . \mathbf {0})$ with $\varGamma ; \emptyset ; \emptyset \vdash a \triangleright \langle \texttt {end} \rangle$, then their derivatives are not bisimilar:

$$\begin{aligned} \begin{array}{rcll} \varGamma ; \emptyset ; \varDelta &{}\vdash &{} P_1 \xrightarrow {s ?\langle \lambda x.\,(\nu \, s')(a !\langle s' \rangle . \mathbf {0}) \rangle } \xrightarrow {~\tau ~} \xrightarrow {~\tau ~}\\ \varDelta &{}\vdash &{} (\nu \, s')(a !\langle s' \rangle . \mathbf {0}) \;|\;(\nu \, s')(a !\langle s' \rangle . \mathbf {0}) \\ \varGamma ; \emptyset ; \varDelta &{}\vdash &{} P_2 \xrightarrow {s ?\langle \lambda x.\,(\nu \, s')(a !\langle s' \rangle . \mathbf {0}) \rangle } \xrightarrow {~\tau ~}\\ \varDelta &{}\vdash &{} (\nu \, s')(a !\langle s' \rangle . \mathbf {0}) \;|\;(\lambda z.\,\mathbf {0})\, {s_2} \end{array} \end{aligned}$$

Observing only the characteristic value results in an under-discriminating bisimulation. However, if a trigger value $\lambda {x}.\,t ?(y) . (y\, {{x}})$ (Definition 14) is received along *s*, we can distinguish $P_1$ and $P_2$ in (3):In the light of this example, one natural question is whether the trigger value suffices to distinguish two processes (hence no need of characteristic values). This is not the case: the trigger value alone also results in an under-discriminating bisimulation relation. In fact, the trigger value can be observed on any input prefix of *any type*. For example, consider processes:

4 $$\begin{aligned}&(\nu \, s)(n ?(x) . (x\, {s}) \;|\;\overline{s} !\langle \lambda x.\,R_1 \rangle . \mathbf {0}) \end{aligned}$$

5 $$\begin{aligned}&(\nu \, s)(n ?(x) . (x\, {s}) \;|\;\overline{s} !\langle \lambda x.\,R_2 \rangle . \mathbf {0}) \end{aligned}$$

If processes in (4) and (5) input the trigger value, we obtain:

$$\begin{aligned} (\nu \, s)(t ?(x) . (x\, {s}) \;|\;\overline{s} !\langle \lambda x.\,R_1 \rangle . \mathbf {0}) \\ (\nu \, s)(t ?(x) . (x\, {s}) \;|\;\overline{s} !\langle \lambda x.\,R_2 \rangle . \mathbf {0}) \end{aligned}$$

thus we can easily derive a bisimulation relation if we assume a definition of bisimulation that allows only trigger value input. But if processes in (4)/(5) input the characteristic value $\lambda z.\,z ?(x) . ( t !\langle z \rangle . \mathbf {0}\;|\;x\, {m})$ (where *m* is a fresh name) then, under appropriate $\varGamma$ and $\varDelta$, they would become:

$$\begin{aligned} \varGamma ; \emptyset ; \varDelta \vdash (\nu \, s)(s ?(x) . (t !\langle s \rangle . \mathbf {0}\;|\;x\, {m}) \;|\;\overline{s} !\langle \lambda x.\,R_i \rangle . \mathbf {0})\ \approx \ \varDelta \vdash R_i m/x \qquad (i=1,2) \end{aligned}$$

which are not bisimilar if $R_1 m/x \not \approx R_2 m/x$.

These examples illustrate the need for both trigger and characteristic values as an input observation in the refined transition relation. This will be the content of Definition 15 below. $\square$

As explained in Sect. 2, we define the *refined* typed LTS by considering a transition rule for input in which admitted values are trigger or characteristic values or names:

#### Definition 15

(*Refined typed labelled transition system*) The refined typed labelled transition relation on typing environmentsis defined on top of the rules in Fig. 5 using the following rules: 
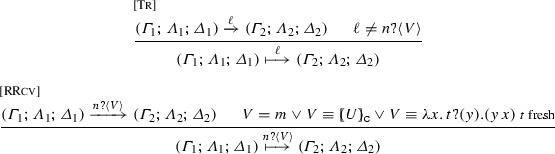
 Then, the refined typed labelled transition systemis given as in Definition 5, replacing the requirement $(\varGamma , \emptyset , \varDelta _1) \xrightarrow {\ell } (\varGamma , \emptyset , \varDelta _2)$ with , as just defined. Following Definition 5, we write  for the reflexive and transitive closure of ,  for the transitions , and  for  if $\ell \not = \tau$ otherwise .

Notice that the (refined) transition  implies the (ordinary) transition $\varGamma ; \varDelta _1 \vdash P_1 \xrightarrow {\,\ell \,} \varDelta _2 \vdash P_2$.

#### Notation 16

We sometimes write  when the type of *V* is *U*.

### Higher-order bisimilarity ($\approx ^\mathtt{H}$) and characteristic bisimilarity ($\approx ^\mathtt{C}$)

Having introduced a refined LTS on $\textsf {HO}\pi$ processes, we now define *higher-order bisimilarity* and *characteristic bisimilarity*, two tractable bisimilarity relations. As explained in Sect. 2, the two bisimulations use two different trigger processes [cf. (2)]:

6 $$\begin{aligned} t \hookleftarrow _{\texttt {H}} V&\mathop {=}\limits ^{\texttt {def}\ }&{\left\{ \begin{array}{ll} t ?(x) . (\nu \, s)(s ?(y) . (x\, {y}) \;|\;\overline{s} !\langle V \rangle . \mathbf {0}) &{} \text {if }V \text { is a first-order value}\\ t ?(x) . (\nu \, s)(s ?(y) . (y\, {x}) \;|\;\overline{s} !\langle V \rangle . \mathbf {0}) &{} \text {if }V \text {is a higher-order value} \end{array}\right. } \end{aligned}$$

7 $$\begin{aligned} t \Leftarrow _{\texttt {C}} V{\,:\,}U&\mathop {=}\limits ^{\texttt {def}\ }&t ?(x) . (\nu \, s)(s ?(y) . [\!\!(U)\!\!]^{y} \;|\;\overline{s} !\langle V \rangle . \mathbf {0}) \end{aligned}$$

The process in (6) is called *higher-order trigger process*, while process in (7) is called *characteristic trigger process*. Notice that while in (6) there is a higher-order input on *t*, in (7) the variable *x* does not play any rôle.

We use higher-order trigger processes to define *higher-order bisimilarity*:

#### Definition 17

(*Higher-order bisimilarity*) A typed relation $\mathfrak {R}$ is a *higher-order bisimulation* if for all $\varGamma ; \varDelta _1 \vdash P_1 \ \mathfrak {R}\ \varDelta _2 \vdash Q_1$

1. Whenever , there exist $Q_2$, $V_2$, $\varDelta '_2$ such that  and, for a fresh *t*,
  $$\begin{aligned} \begin{array}{lrlll} \varGamma ; \varDelta ''_1 \vdash {(\nu \, \widetilde{m_1})(P_2 \;|\;t \hookleftarrow _{\texttt {H}} V_1)} \ \mathfrak {R}\ \varDelta ''_2 \vdash {(\nu \, \widetilde{m_2})(Q_2 \;|\;t \hookleftarrow _{\texttt {H}} V_2)} \end{array} \end{aligned}$$
2. For all  such that $\ell$ is not an output, there exist $Q_2$, $\varDelta '_2$ such that  and $\varGamma ; \varDelta _1' \vdash P_2 \ \mathfrak {R}\ \varDelta _2' \vdash Q_2$; and
3. The symmetric cases of 1 and 2.

The largest such bisimulation is called *higher-order bisimilarity*, denoted by $\approx ^\mathtt{H}$.

We exploit characteristic trigger processes to define *characteristic bisimilarity*:

#### Definition 18

(*Characteristic bisimilarity*) A typed relation $\mathfrak {R}$ is a *characteristic bisimulation* if for all $\varGamma ; \varDelta _1 \vdash P_1 \ \mathfrak {R}\ \varDelta _2 \vdash Q_1$,

1. Whenever  then there exist $Q_2$, $V_2$, $\varDelta '_2$ such that  and, for a fresh *t*,
  $$\begin{aligned} \varGamma ; \varDelta ''_1 \vdash {(\nu \, \widetilde{m_1})(P_2 \;|\;t \Leftarrow _{\texttt {C}} V_1{\,:\,}U_1)} \ \mathfrak {R}\ \varDelta ''_2 \vdash {(\nu \, \widetilde{m_2})(Q_2 \;|\;t \Leftarrow _{\texttt {C}} V_2{\,:\,}U_2)} \end{aligned}$$
2. For all  such that $\ell$ is not an output, there exist $Q_2$, $\varDelta '_2$ such that  and $\varGamma ; \varDelta _1' \vdash P_2 \ \mathfrak {R}\ \varDelta _2' \vdash Q_2$; and
3. The symmetric cases of 1 and 2.

The largest such bisimulation is called *characteristic bisimilarity*, denoted by $\approx ^\mathtt{C}$.

Observe how we have used Notation 16 to explicitly refer to the type of the emitted value in output actions.

#### Remark 1

(Differences between $\approx ^\mathtt{H}$ and $\approx ^\mathtt{C}$) Although $\approx ^\mathtt{H}$ and $\approx ^\mathtt{C}$ are conceptually similar, they differ in the kind of trigger process considered. Because of the application in $t \hookleftarrow _{\texttt {H}} V$ (cf. (6)), $\approx ^\mathtt{H}$ cannot be used to reason about first-order session processes (i.e., processes without higher-order features). In contrast, $\approx ^\mathtt{C}$ is more general: it can uniformly input characteristic, first- or higher-order values.

### Deterministic transitions and up-to techniques

As hinted at earlier, internal transitions associated to session interactions or $\beta$-reductions are deterministic. To define an auxiliary proof technique that exploits determinacy we require some auxiliary definitions.

#### Definition 19

(*Deterministic transitions*) Suppose $\varGamma ; \emptyset ; \varDelta \vdash P \triangleright \diamond$ with balanced $\varDelta$. Transition  is called:

- session-transition whenever transition $P \xrightarrow {\tau } P'$ is derived using Rule ${\langle \textsc {Tau} \rangle }$ (where $\texttt {subj}(\ell _1)$ and $\texttt {subj}(\ell _2)$ in the premise are dual endpoints), possibly followed by uses of Rules ${\langle \textsc {Alpha} \rangle }$, ${\langle \textsc {Res} \rangle }$, ${\langle \textsc {Rec} \rangle }$, or 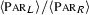 (cf. Fig. 4).
- a $\beta$-*transition* whenever transition  is derived using Rule ${\langle \textsc {App} \rangle }$, possibly followed by uses of Rules ${\langle \textsc {Alpha} \rangle }$, ${\langle \textsc {Res} \rangle }$, ${\langle \textsc {Rec} \rangle }$, or 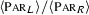 (cf. Fig. 4).

#### Notation 20

We use the following notations:

denotes a session-transition.
denotes a $$\beta $$-transition.
denotes either a session-transition or a $\beta$-transition.
- We write  to denote a (possibly empty) sequence of deterministic steps .

Deterministic transitions imply the $\tau$-inertness property [7], which ensures behavioural invariance on deterministic transitions:

#### Proposition 2

($\tau$-inertness) Suppose $\varGamma ; \emptyset ; \varDelta \vdash P \triangleright \diamond$ with balanced $\varDelta$. Then

implies $$\varGamma ; \varDelta \vdash P \approx ^\mathtt{H} \varDelta ' \vdash P'$$.
implies $$\varGamma ; \varDelta \vdash P \approx ^\mathtt{H} \varDelta ' \vdash P'$$.

#### Proof

(Sketch) The proof of Part 1 requires to show that relation (we omit type information)is a higher-order bisimulation. The proof for Part 2 is direct from Part 1. See “Deterministic transitions” section of Appendix 2 for the details. $\square$

Using the above properties, we can state the following up-to technique.

#### Lemma 1

(Up-to deterministic transition) Let $\varGamma ; \varDelta _1 \vdash P_1 \ \mathfrak {R}\ \varDelta _2 \vdash Q_1$ such that if whenever:

1. $\forall (\nu \, \widetilde{m_1}) n !\langle V_1 \rangle$ such that  implies that $\exists Q_2, V_2$ such that  and  and for a fresh name *t* and $\varDelta _1'', \varDelta _2''$:
  $$\begin{aligned} \varGamma ; \varDelta _1'' \vdash (\nu \, \widetilde{m_1})(P_2 \;|\;t \hookleftarrow _{\texttt {H}} V_1) \ \mathfrak {R}\ \varDelta _2'' \vdash {(\nu \, \widetilde{m_2})(Q_2 \;|\;t \hookleftarrow _{\texttt {H}} V_2)} \end{aligned}$$
2. $\forall \ell \not = (\nu \, \widetilde{m}) n !\langle V \rangle$ such that  implies that $\exists Q_2$ such that  and  and $\varGamma ; \varDelta _1' \vdash P_2 \ \mathfrak {R}\ \varDelta _2' \vdash Q_2$.
3. The symmetric cases of 1 and 2.

Then $$\mathfrak {R}\ \subseteq \ \approx ^\mathtt{H}$$.

#### Proof

(Sketch) The proof proceeds by considering the relationWe may verify that  is a higher-order bisimulation by using Proposition 2. $\square$

### Characterisation of higher-order and characteristic bisimilarities

This section proves the main result; it allows us to use $\approx ^\mathtt{C}$ and $\approx ^\mathtt{H}$ as tractable reasoning techniques for $\textsf {HO}\pi$ processes.

#### Lemma 2

$$\approx ^\mathtt{C}\ =\ \approx ^\mathtt{H}$$.

#### Proof

(Sketch) The main difference between $\approx ^\mathtt{H}$ and $\approx ^\mathtt{C}$ is the trigger process (higher-order triggers $t \hookleftarrow _{\texttt {H}} V$ in $\approx ^\mathtt{H}$ and characteristic triggers $t \Leftarrow _{\texttt {C}} V{\,:\,}U$ in $\approx ^\mathtt{C}$). Thus, the most interesting case in the proof is when we observe an output from a process. When showing that $\approx ^\mathtt{C}\ \subseteq \ \approx ^\mathtt{H}$, the key after the output is to show that

$$\begin{aligned} (\nu \, \tilde{m_1})(P_1 \;|\;t \Leftarrow _{\texttt {C}} V{\,:\,}U) \approx ^\mathtt{H}(\nu \, \tilde{m_2})(P_2 \;|\;t \Leftarrow _{\texttt {C}} V_2{\,:\,}U) \end{aligned}$$

given that

$$\begin{aligned} (\nu \, \tilde{m_1})(P_1 \;|\;t \hookleftarrow _{\texttt {H}} V) \approx ^\mathtt{H}(\nu \, \tilde{m_2})(P_2 \;|\;t \hookleftarrow _{\texttt {H}} V_2). \end{aligned}$$

Similarly, in the proof of $\approx ^\mathtt{H}\ \subseteq \ \approx ^\mathtt{C}$, the key step is showing that

$$\begin{aligned} (\nu \, \tilde{m_1})(P_1 \;|\;t \hookleftarrow _{\texttt {H}} V) \approx ^\mathtt{C}(\nu \, \tilde{m_2})(P_2 \;|\;t \hookleftarrow _{\texttt {H}} V_2) \end{aligned}$$

given that

$$\begin{aligned} (\nu \, \tilde{m_1})(P_1 \;|\;t \Leftarrow _{\texttt {C}} V{\,:\,}U) \approx ^\mathtt{C}(\nu \, \tilde{m_2})(P_2 \;|\;t \Leftarrow _{\texttt {C}} V_2{\,:\,}U). \end{aligned}$$

The proof for the above equalities is coinductive, exploiting the freshness of the trigger name in each case; see Lemma 13 in the Appendix. While the proof of the first equality (i.e., higher-order triggers imply characteristic triggers) follows expected lines, the proof of the second equality (i.e., characteristic triggers imply higher-order triggers) is a bit more involved. Indeed, while higher-order trigger processes can input trigger values, characteristic triggers cannot. However, we prove that this does not represent a difference in behaviour; see case 2(c) in Lemma 13. To this end, we exploit an alternative trigger process, denoted $t \leftharpoonup _\texttt {A} V$, simpler than the higher-order trigger $t \hookleftarrow _{\texttt {H}} V$ in (6):

$$\begin{aligned} t \leftharpoonup _\texttt {A} V = t ?(x) . (\nu \, s)(x\, {s} \;|\;\overline{s} !\langle V \rangle . \mathbf {0}) \end{aligned}$$

In the proofs for these coincidence results, we exploit some auxiliary results for trigger processes, including a two-way connection between $t \hookleftarrow _{\texttt {H}} V$ and $t \leftharpoonup _\texttt {A} V$ (cf. Lemma 12 (3) in the Appendix). We thus infer that characteristic trigger processes $t \Leftarrow _{\texttt {C}} V{\,:\,}U$ and higher-order trigger processes $t \hookleftarrow _{\texttt {H}} V$ exhibit a similar behaviour.

In turn, using the above results we can show that typed relations induced by $\approx ^\mathtt{H}$ and $\approx ^\mathtt{C}$ coincide. The full proof is in “Proof of Theorem 2” section in Appendix 2, Lemma 14. $\square$

The next lemma is crucial for the characterisation of higher-order and characteristic bisimilarities. It states that if two processes are equivalent under the trigger value then they are equivalent under any higher-order substitution.

#### Lemma 3

(Process substitution) Let *P* and *Q* be two processes and some fresh *t*. If

$$\begin{aligned} \varGamma ; \varDelta _1' \vdash P \lambda x.\,t ?(y) . (y\, {x})/z \approx ^\mathtt{H} \varDelta '_2 \vdash Q \lambda x.\,t ?(y) . (y\, {x})/z \end{aligned}$$

then for all *R* such that $\texttt {fv}(R) = \{x\}$, we have

$$\begin{aligned} \varGamma ; \varDelta _1 \vdash P \lambda x.\,R/z \approx ^\mathtt{H} \varDelta _2 \vdash Q \lambda x.\,R/z. \end{aligned}$$

The full proof of Lemma 3 can be found in “Proof of Theorem 2” section in Appendix 2, Lemma 17; it is obtained by (i) constructing a typed relation on the substitution properties stated by the lemma and (ii) proving that it is a higher-order bisimulation, using the auxiliary result given next. In the following, given a finite index set $I = \{1, \ldots , n\}$, we shall write $\prod _{i \in I} P_i$ to stand for $P_1 \;|\;P_2 \;|\;\cdots \;|\;P_n$.

#### Lemma 4

(*Trigger substitution*) Let *P* and *Q* be processes. Also, let *t* be a fresh name. Ifthen for all $\lambda \widetilde{x}.\,R$, there exist $\varDelta _1', \varDelta _2'$ such that

$$\begin{aligned} \varGamma ; \varDelta _1' \vdash (\nu \, \widetilde{m_1})(P \;|\;(\lambda \widetilde{x}.\,R)\, {\widetilde{n}} ) \approx ^\mathtt{H} \varDelta _2' \vdash (\nu \, \widetilde{m_2})(Q \;|\;(\lambda \widetilde{x}.\,R)\, {\widetilde{m}} ). \end{aligned}$$

#### Proof

(Sketch) The proof follows the definition of the characteristic process; see Lemma 16, in the Appendix for details. Let us consider the particular case in which *I* is a singleton; we then construct a typed relation $\mathfrak {R}$:

$$\begin{aligned} \mathfrak {R}= & {} \{ \varGamma ; \varDelta _1' \vdash (\nu \, \widetilde{m_1})(P \;|\;(\lambda x.\,R)\, {n_1} ) \ ,\ \varDelta _2' \vdash (\nu \, \widetilde{m_2})(Q \;|\;(\lambda x.\,R)\, {n_2} ) \ \ |\ \ \\&\varGamma ; \varDelta _1 \vdash (\nu \, \widetilde{m_1})(P \;|\;(\lambda x.\,t ?(y) . (y\, {x}))\, {n_1} ) \approx ^\mathtt{H} \varDelta _2 \vdash (\nu \, \widetilde{m_2})(Q \;|\;(\lambda x.\,t ?(y) . (y\, {x}))\, {n_2} ) \} \end{aligned}$$

The typed relation $\mathfrak {R}$ can be shown to be a higher-order bisimulation by taking advantage of the shape of the characteristic process; each time that a characteristic process does a transition, an output $t !\langle n \rangle . \mathbf {0}$ (on a fresh name *t*) is observed, where *n* is either a shared or a session name. To better illustrate this, let us sketch the demanding case of the proof that $\mathfrak {R}$ is a higher-order bisimulation. Assume thatfor some $\varDelta ''_1$. Then, from the definition of $\mathfrak {R}$, we have:for some $\varDelta _3$. Characteristic processes have the following property, for any $U \ne \texttt {end}$:

$$\begin{aligned}{}[\!\!(U)\!\!]^{n} \xrightarrow {\ell } t !\langle n \rangle . \mathbf {0}\end{aligned}$$

By the last property we can always observe, for some $\varDelta ''_3$ (note that below $\ell _1$ may be an action $\tau$, thus denoting the interaction of *P* and $[\!\!(U)\!\!]^{n_1}$):which implies, from the requirements of higher-order bisimulation, that there exist $(\nu \, \widetilde{m_2}'')(Q' \;|\;[\!\!(U)\!\!]^{x} n_2/x )$ and $\varDelta _4$ such thatBy the shape of the characteristic process we can always observe for $\ell _2, \texttt {subj}(\ell _2) = \texttt {subj}(\ell _1)$ if $\ell _1$ is output, and $\ell _2 = \ell _1$ otherwise, that:8for some $\varDelta '_4$ and

9 $$\begin{aligned} \varGamma ; \varDelta _3'' \vdash (\nu \, \widetilde{m_1}''')(P' \;|\;t'' \hookleftarrow _{\texttt {H}} n_1 ) \approx ^\mathtt{H} \varDelta _4'' \vdash (\nu \, \widetilde{m_2}''')(Q'' \;|\;t'' \hookleftarrow _{\texttt {H}} n_2 ) \end{aligned}$$

for some $\varDelta ''_4$. From (8) we getfor some $\varDelta ''_2$ and from (9) we get

$$\begin{aligned} \varGamma ; \varDelta _3'' \vdash (\nu \, \widetilde{m_1}''')(P' \;|\;(\lambda x.\,t'' ?(y) . (y\, {x}))\, {n_1} ) \approx ^\mathtt{H} \varDelta _4'' \vdash (\nu \, \widetilde{m_2}''')(Q'' \;|\;(\lambda x.\,t'' ?(y) . (y\, {x}))\, {n_2} ) \end{aligned}$$

which implies from the definition of $\mathfrak {R}$ that for $R'$ we get

$$\begin{aligned} \varGamma ; \varDelta _1'' \vdash (\nu \, \widetilde{m_1}')(P' \;|\;R' n_1/x ) \ \mathfrak {R}\ \varDelta _2'' \vdash (\nu \, \widetilde{m_2}')(Q'' \;|\;R' n_2/x ) \end{aligned}$$

as required. $$\square $$

We now show that higher-order bisimilarity is sound with respect to context bisimilarity. To show soundness we use the crucial result of Lemma 3:

#### Lemma 5

$$\approx ^\mathtt{H}\ \subseteq \ \approx $$.

#### Proof

(Sketch) The proof relies on Lemma 3 to establish that:

1. Whenever two processes are higher-order bisimilar under the input of a characteristic value and a trigger value then they are higher-order bisimilar under the input of any value $\lambda x.\,R$, which is the requirement for $\approx$ (cf. Definition 12).
2. The input requirement is then further used to prove that the output clause requirement for $\approx ^\mathtt{H}$ (cf. Definition 17):
  $$\begin{aligned} \begin{array}{lrlll} \varGamma ; \varDelta _1 \vdash {(\nu \, \widetilde{m_1})(P_2 \;|\;t \hookleftarrow _{\texttt {H}} V_1)} \ \mathfrak {R}\ \varDelta _2 \vdash {(\nu \, \widetilde{m_2})(Q_2 \;|\;t \hookleftarrow _{\texttt {H}} V_2)} \end{array} \end{aligned}$$
  implies the output clause requirement for $\approx$, that is, for all *R* with $\texttt {fv}(R)=\{x\}$:
  $$\begin{aligned} \varGamma ; \varDelta _1 \vdash (\nu \, \widetilde{m_1})(P_2 \;|\;RV_1/x) \ \mathfrak {R}\ \varDelta _2 \vdash (\nu \, \widetilde{m_2})(Q_2 \;|\;RV_2/x). \end{aligned}$$

The full proof is found in “Proof of Theorem 2” section in Appendix 2, Lemma 18. $\square$

Context bisimilarity is included in barbed congruence:

#### Lemma 6

$$\approx \ \subseteq \ \cong $$.

#### Proof

(Sketch) We show that $\approx$ satisfies the defining properties of $\cong$. It is easy to show that $\approx$ is reduction-closed and barb preserving (cf. Definition 6 and Definition 9). The most challenging part is to show that $\approx$ is a congruence, in particular a congruence with respect to parallel composition. To this end, we construct the following relation:

$$\begin{aligned} {\mathcal {S}}= & {} \{ (\varGamma ; \emptyset ; \varDelta _1 \cdot \varDelta _3 \vdash (\nu \, \widetilde{n_1})(P_1 \;|\;R) \ ,\ \varGamma ; \emptyset ; \varDelta _2 \cdot \varDelta _3 \vdash (\nu \, \widetilde{n_2})(P_2 \;|\;R)) \ \ |\ \ \\&\qquad \qquad \qquad \varGamma ; \varDelta _1 \vdash P_1 \approx \varDelta _2 \vdash P_2 \quad \text{ and } \quad \forall R \text{ such } \text{ that } \varGamma ; \emptyset ; \varDelta _3 \vdash R \triangleright \diamond \} \end{aligned}$$

We show that ${\mathcal {S}}$ is a context bisimulation by a case analysis on the transitions of the pairs in ${\mathcal {S}}$. The full proof is found in “Proof of Theorem 2” section in Appendix 2, Lemma 19. $\square$

The last ingredient required for our main result is the following inclusion.

#### Lemma 7

$$\cong \ \subseteq \ \approx ^\mathtt{H}$$.

#### Proof

(Sketch) The proof exploits the *definability* technique developed in [8, § 6.7] and refined for session types in [18, 19]. Intuitively, this technique exploits small test processes that reveal the presence of a visible action by reducing with a given pair of processes and exhibiting a barb on a fresh name.

Intuitively, for each visible action $\ell$, we use a fresh name $succ$ to we define a (typed) test process $\varGamma ; \emptyset ; \varDelta _2 \vdash T\langle \ell , succ \rangle \triangleright \diamond$ with the following property:See Definition 25 for the formal definition. The test processes can therefore be used to check the typed labelled transition interactions of two processes that are related by reduction-closed, barbed congruence. Indeed, we have that

$$\begin{aligned} \varGamma ; \varDelta _1 \vdash P\ \cong \ \varDelta _2 \vdash Q \end{aligned}$$

implies from congruence of $\cong$, that if there exist $\varDelta _3, \varDelta _4$ such that:

$$\begin{aligned} \varGamma ; \varDelta _3 \vdash P \;|\;T\langle \ell , succ \rangle \ \cong \ \varDelta _4 \vdash Q \;|\;T\langle \ell , succ \rangle \end{aligned}$$

then it implies from reduction-closeness of $\cong$ and the definition of $T\langle \ell , succ \rangle$:

10 $$\begin{aligned} \varGamma ; \varDelta _3' \vdash P' \;|\;succ !\langle \overline{m} \rangle . \mathbf {0}\ \cong \ \varDelta _4' \vdash Q' \;|\;succ !\langle \overline{m} \rangle . \mathbf {0} \end{aligned}$$

which in turn means that whenever $\varGamma ; \varDelta _1 \vdash P \triangleright \diamond$ can perform an action  then we can derive that $\varGamma ; \varDelta _2 \vdash Q \triangleright \diamond$ can also perform action  because of the result in (10). By applying Lemma 21 on (10) we can deduce that $\varGamma ; \varDelta _1' \vdash P'\ \cong \ \varDelta _2' \vdash Q'$. This concludes the requirements of $\approx$:

$$\begin{aligned} \varGamma ; \varDelta \vdash P\ \approx ^\mathtt{H}\ \varDelta ' \vdash Q \end{aligned}$$

The full details can be found in “Proof of Theorem 2” section in Appendix 2, Lemma 22. $\square$

We can finally state our main result:

#### Theorem 2

(Coincidence) $\cong$, $\approx$, $\approx ^\mathtt{H}$ and $\approx ^\mathtt{C}$ coincide in $\textsf {HO}\pi$.

#### Proof

The proof is a direct consequence from our previous results: Lemma 2 (which proves $\approx ^\mathtt{H}\ =\ \approx ^\mathtt{C}$), Lemma 5 (which proves $\approx ^\mathtt{H}\ \subseteq \ \approx$), Lemma 6 (which proves $\approx \ \subseteq \ \cong$), and Lemma 7 (which proves $\cong \ \subseteq \ \approx ^\mathtt{H}$). Indeed, we may conclude

$$\begin{aligned} \cong \ \subseteq \ \approx ^\mathtt{H}\ =\ \approx ^\mathtt{C}\ \subseteq \ \approx \ \subseteq \ \cong \end{aligned}$$

$$\square $$

**Fig. 7:**
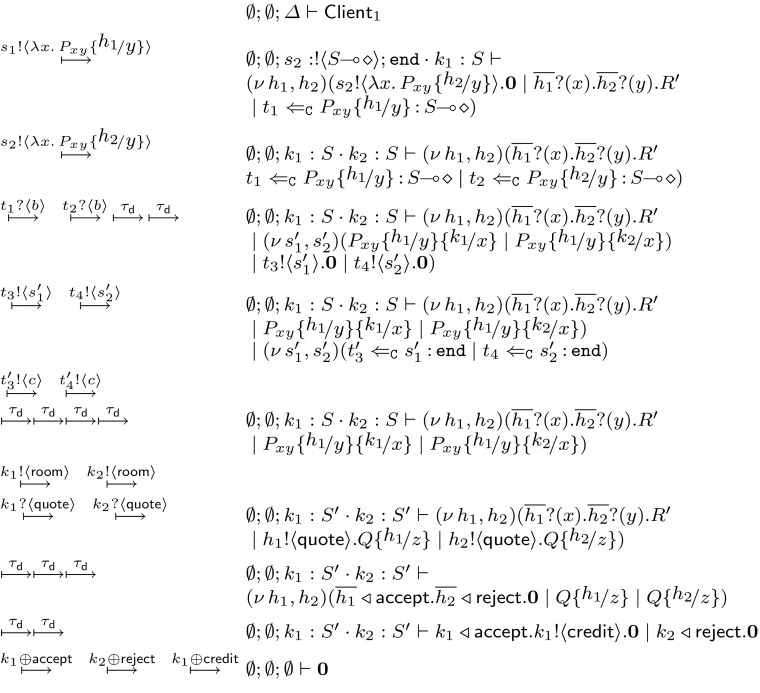
Observable actions from $\textsf {Client}_1$ (cf. Sect. 5.9)

### Revisiting the hotel booking scenario (Sect. 3.3)

Now we revisit our running example to prove that $\textsf {Client}_1$ and $\textsf {Client}_2$ in Sect. 3.3 are behaviourally equivalent.

#### Proposition 3

Let $S = !\langle \textsf {room} \rangle ; ?(\textsf {quote}) ; \oplus \{\textsf {accept}: !\langle \textsf {credit} \rangle ; \texttt {end}, \textsf {reject}: \texttt {end}\}$ and $\varDelta = s_1: !\langle S\!\! \multimap \! \diamond \rangle ; \texttt {end}\cdot s_2: !\langle S\!\! \multimap \! \diamond \rangle ; \texttt {end}$. Then $\emptyset ; \varDelta \vdash \textsf {Client}_1 \approx ^\mathtt{C} \varDelta \vdash \textsf {Client}_2$, where $\textsf {Client}_1$ and $\textsf {Client}_2$ are as in Sect. 3.3.

#### Proof

We show a case where each typed process simulates the other, according to the definition of $\approx ^\mathtt{C}$ (cf. Definition 18). In order to show the bisimulation game consider the definition of the characteristic process for type $?(S\!\! \multimap \! \diamond ) ; \texttt {end}$. For fresh sessions *s*, *k*, we have

$$\begin{aligned} {[\!\!(?(S\!\! \multimap \! \diamond ) ; \texttt {end})\!\!]^{s} = s ?(x) . ( t !\langle s \rangle . \mathbf {0}\;|\;[\!\!(S\!\! \multimap \! \diamond )\!\!]^{x})} \end{aligned}$$

For convenience, we recall the definition of $\textsf {Client}_1$:

$$\begin{aligned} \textsf {Client}_1&\mathop {=}\limits ^{\texttt {def}\ }&(\nu \, h_1, h_2)(s_1 !\langle \lambda x.\,P_{xy} h_1/y \rangle . s_2 !\langle \lambda x.\,P_{xy} h_2/y \rangle . \mathbf {0}\;|\;\overline{h_1} ?(x) . \overline{h_2} ?(y) . R' ) \end{aligned}$$

where

$$\begin{aligned}&P_{xy} \mathop {=}\limits ^{\texttt {def}\ }x !\langle \textsf {room} \rangle . x ?(\textsf {quote}) . y !\langle \textsf {quote} \rangle . y \triangleright \left\{ \begin{array}{l} \textsf {accept}: x \triangleleft \textsf {accept} . x !\langle \textsf {credit} \rangle . \mathbf {0}~,\\ \textsf {reject}: x \triangleleft \textsf {reject} . \mathbf {0}\end{array} \right\} \\&\quad R' \equiv \texttt {if}\ \ x \le y\ \texttt {then}\ (\overline{h_1} \triangleleft \textsf {accept} . \overline{h_2} \triangleleft \textsf {reject} . \mathbf {0}~~ \varvec{;} ~~\overline{h_1} \triangleleft \textsf {reject} . \overline{h_2} \triangleleft \textsf {accept} . \mathbf {0}) \end{aligned}$$

Also, the definition of $\textsf {Client}_2$ is as follows:

$$\begin{aligned}&\textsf {Client}_2 \mathop {=}\limits ^{\texttt {def}\ }(\nu \, h)(s_1 !\langle \lambda x.\,Q_1 h/y \rangle . s_2 !\langle \lambda x.\,Q_2 \overline{h}/y \rangle . \mathbf {0}) \\&\quad Q_1 \mathop {=}\limits ^{\texttt {def}\ }x !\langle \textsf {room} \rangle . x ?(\textsf {quote}_1) . y !\langle \textsf {quote}_1 \rangle . y ?(\textsf {quote}_2) . R_x \\&\quad Q_2 \mathop {=}\limits ^{\texttt {def}\ }x !\langle \textsf {room} \rangle . x ?(\textsf {quote}_1) . y ?(\textsf {quote}_2) . y !\langle \textsf {quote}_1 \rangle . R_x \\&\quad R_x \mathop {=}\limits ^{\texttt {def}\ }\texttt {if}\ \ \textsf {quote}_1 \le \textsf {quote}_2 \, \texttt {then}\ (x \triangleleft \textsf {accept} . x !\langle \textsf {credit} \rangle . \mathbf {0}\ \varvec{;} \ x \triangleleft \textsf {reject} . \mathbf {0}) \end{aligned}$$

A detailed account of the observable behaviour of $\textsf {Client}_1$ is given in Fig. 7, where we use the following shorthand notation:

$$\begin{aligned} Q \equiv z \triangleright \{\textsf {accept}: k_2 \triangleleft \textsf {accept} . k_2 !\langle \textsf {credit} \rangle . \mathbf {0}, \textsf {reject}: k_2 \triangleleft \textsf {reject} . \mathbf {0}\} \end{aligned}$$

**Fig. 8:**
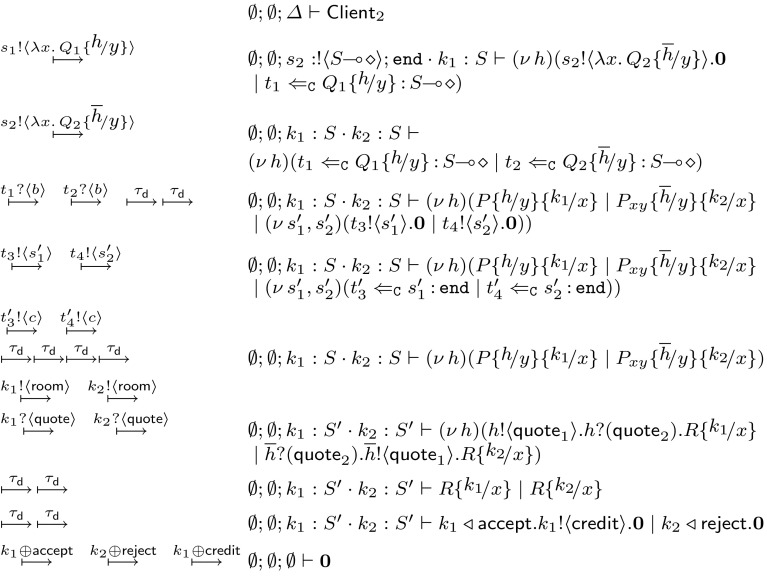
Observable actions from $\textsf {Client}_2$ (cf. Sect. 5.9)

Similarly, Fig. 8 illustrates the actions possible from $\textsf {Client}_2$, which are the same as for $\textsf {Client}_1$. $\square$

## Related work

Since types can limit contexts (environments) where processes can interact, typed equivalences usually offer *coarser* semantics than untyped equivalences. Pierce and Sangiorgi [28] demonstrated that IO-subtyping can justify the optimal encoding of the $\lambda$-calculus by Milner—this was not possible in the untyped polyadic $\pi$-calculus [23]. After [28], several works on typed $\pi$-calculi have investigated correctness of encodings of known concurrent and sequential calculi in order to examine semantic effects of proposed typing systems.

A type discipline closely related to session types is a family of linear typing systems. Kobayashi, Pierce, and Turner [14] first proposed a linearly typed reduction-closed, barbed congruence and used it to reason about a tail-call optimisation of higher-order functions encoded as processes. Yoshida [35] used a bisimulation of graph-based types to prove the full abstraction of encodings of the polyadic synchronous $\pi$-calculus into the monadic synchronous $\pi$-calculus. Later, typed equivalences of a family of linear and affine calculi [2, 3, 36] were used to encode PCF [22, 29], the simply typed $\lambda$-calculus with sums and products, and System F [6] fully abstractly (a fully abstract encoding of the $\lambda$-calculi was an open problem in [23]). Yoshida et al. [37] proposed a new bisimilarity method associated with a linear type structure and strong normalisation; it presented applications to reason about secrecy in programming languages. A subsequent work [11] adapted these results to a practical direction, proposing new typing systems for secure higher-order and multi-threaded programming languages. In these works, typed properties, linearity and liveness, play a fundamental rôle in the analysis. In general, linear types are suitable to encode “sequentiality” in the sense of [1, 12].

Our work follows the behavioural semantics in [18, 19, 27] where a bisimulation is defined on an LTS that assumes a session typed observer. Our theory for higher-order sessions differentiates from the work in [19] and [18], which considers (first-order) binary and multiparty session types, respectively. Pérez et al [27] studied typed equivalences for a theory of binary sessions based on linear logic, without shared names.

Our approach to typed equivalences builds upon techniques developed by Sangiorgi [30, 31] and Jeffrey and Rathke [13]. As we have discussed, although context bisimilarity has a satisfactory discriminative power, its use is hindered by the universal quantification on output. To deal with this, Sangiorgi proposes *normal bisimilarity*, a tractable equivalence without universal quantification. To prove that context and normal bisimilarities coincide, the approach in [30] uses triggered processes. Triggered bisimulation is also defined on first-order labels where context bisimulation is restricted to arbitrary trigger substitution. This characterisation of context bisimilarity was refined in [13] for calculi with recursive types, not addressed in [30, 31] and quite relevant in session-based concurrency. The bisimulation in [13] is based on an LTS extended with trigger meta-notation. As in [30, 31], the LTS in [13] observes first-order triggered values instead of higher-order values, offering a more direct characterisation of contextual equivalence and lifting the restriction to finite types. *Environmental bisimulations* [32] use a higher-order LTS to define a bisimulation that stores the observer’s knowledge; hence, observed actions are based on this knowledge at any given time. This approach is enhanced in [15] with a mapping from constants to higher-order values. This allows to observe first-order values instead of higher-order values. It differs from [13, 31] in that the mapping between higher- and first-order values is no longer implicit.

*Comparison with respect to*[13] We briefly contrast the approach by Jeffrey and Rathke [13] and our approach based on characteristic bisimilarity ($\approx ^\mathtt{C}$):

- The LTS in [13] is enriched with extra labels for triggers; an output action transition emits a trigger and introduces a parallel replicated trigger. Our approach retains usual labels/transitions; in case of output, $\approx ^\mathtt{C}$ introduces a parallel *non-replicated* trigger.
- Higher-order input in [13] involves the input of a trigger which reduces after substitution. Rather than a trigger name, $\approx ^\mathtt{C}$ decrees the input of a trigger value $\lambda z.\,t ?(x) . (x\, {z})$.
- Unlike [13], $\approx ^\mathtt{C}$ treats first- and higher-order values uniformly. As the typed LTS distinguishes linear and shared values, replicated closures are used only for shared values.
- In [13] name matching is crucial to prove completeness of bisimilarity. In our case, $\textsf {HO}\pi$ lacks name matching and we use session types: a characteristic value inhabiting a type enables the simplest form of interactions with the environment.

To further compare our approach to that in [13], we use a representative example.

### Example 6

**Fig. 9:**
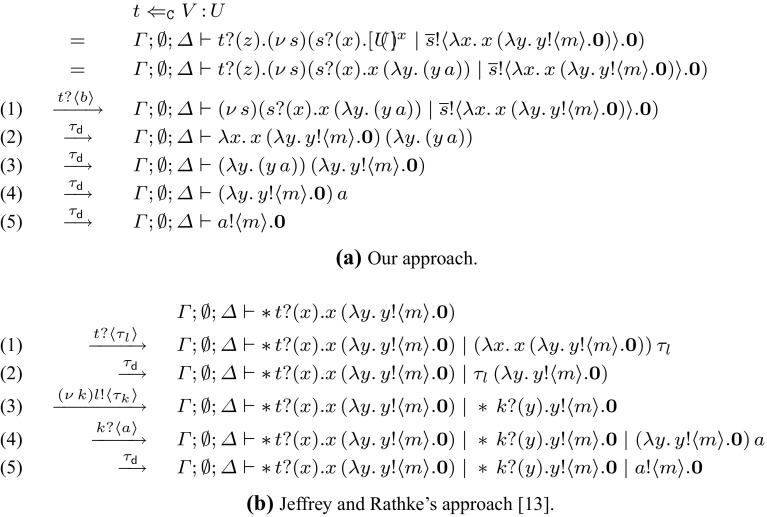
Comparing labelled transitions associated to the process in Example 6

Let $V = \lambda x.\,x\, {(}\lambda y.\,y !\langle m \rangle . \mathbf {0})$ be a value. Consider a process such that

$$\begin{aligned} \varGamma ; \emptyset ; \varDelta \cdot n: !\langle U \rangle ; \texttt {end}\vdash n !\langle V \rangle . \mathbf {0}\triangleright \diamond \end{aligned}$$

with $U = (((!\langle S \rangle ; \texttt {end})\!\! \rightarrow \! \diamond )\!\! \rightarrow \! \diamond )\!\! \rightarrow \! \diamond$. We contrast the transitions from *P*. In our framework, we have a typed transition $\varGamma ; \emptyset ; \varDelta \cdot n: !\langle U \rangle ; \texttt {end}\vdash P \xrightarrow {n !\langle V \rangle }\varGamma ; \emptyset ; \varDelta \vdash \mathbf {0}$. In the framework of [13] a similar (but untyped) output transition takes place. Figure 9 presents a complete comparison of the labelled transitions in our approach (Fig. 9a) and in [13] (Fig. 9b). In our approach, we let

$$\begin{aligned}{}[\!\!(U)\!\!]^{x} = x\, {(\lambda y.\,(y\, {a}))}\qquad \hbox { for some fresh}\ a \end{aligned}$$

Then we have one input transition (Line (1)), followed by four deterministic internal transitions; no replicated processes are needed. The approach of [13] also uses five transitions, but more visible transitions are required (three, see Lines (1), (2), and (3) in Fig. 9b) and at the end, two replicated processes remain (on *t* and *k*). This is how linearity information in session types enables simpler bisimulations. Note that $\tau _l$ and $\tau _k$ in Lines (1) and (3) denote triggered processes on names *l* and *k*.

The previous comparison shows how our approach requires less visible transitions and replicated processes. Therefore, linearity information does simplify analyses, as it enables simpler witnesses in coinductive proofs.

## Concluding remarks

Obtaining tractable characterisations of contextual equivalence is a long-standing issue for higher-order languages. In this paper, we have addressed this challenge for a higher-order language which integrates functional constructs and features from concurrent processes (name and process passing), and whose interactions are governed by *session types*, a behavioural type discipline for structured communications. The main result of our study is the development of *characteristic bisimilarity*, a relation on session typed processes which fully characterises contextual equivalence.

Compared to the well-known context bisimilarity, our notion of characteristic bisimilarity enables more tractable analyses without sacrificing distinguishing power. Our approach to simplified analysis rests upon two simple mechanisms. First, using *trigger processes* we lighten the requirements involved in output clauses. In particular, we can lift the need for heavy universal quantifications. Second, using *characteristic processes and values* we refine the requirements for input clauses. Formally supported by a refined LTS, the use of characteristic processes and values effectively narrows down input actions. Session type information (which includes linearity requirements on reciprocal communications), naturally available in scenarios of interacting processes, is crucial to define these two new mechanisms, and therefore to enable technical simplifications in our developments. As already discussed, our coincidence result is insightful also in the light of previous works on labelled equivalences for higher-order processes, in particular with respect to characterisations by Sangiorgi [30, 31] and by Jeffrey and Rathke [13]. Our main result combines several technical innovations, including, e.g., up-to techniques for deterministic behaviours (cf. Lemma 1) and an alternative behavioural equivalence, called *higher-order bisimilarity* (denoted $\approx ^\mathtt{H}$, cf. Definition 17), which uses simpler trigger processes and is applicable to processes without first-order passing.

In addition to their intrinsic significance, our study has important consequences and applications in other aspects of the theory of higher-order processes. In particular, we have recently explored the *relative expressivity* of higher-order sessions [17]. Both characteristic and higher-order bisimilarities play an important rôle in establishing tight correctness properties (e.g., operational correspondence and full abstraction) for encodability results connecting different variants of $\textsf {HO}\pi$. Such variants cover features such as pure process passing (with first- and higher-order abstractions), pure name passing, polyadicity, linear/shared communication.

## Appendix 1: The typing system of $\textsf {HO}\pi$

We first formally define *type equivalence*. Then we give details of the proof of Theorem 1.

### Type equivalence

#### Definition 21

(*Type equivalence*) Let ${\mathsf {S}}{\mathsf {T}}$ be a set of closed session types. Two types *S* and $S'$ are said to be *isomorphic* if a pair $(S,S')$ is in the largest fixed point of the monotone function $F:{\mathcal {P}}({\mathsf {S}}{\mathsf {T}}\times {\mathsf {S}}{\mathsf {T}}) \rightarrow {\mathcal {P}}({\mathsf {S}}{\mathsf {T}}\times {\mathsf {S}}{\mathsf {T}})$ defined by:

$$ \begin{aligned} F(\mathfrak {R})= & {} \{(\texttt {end}, \texttt {end})\}\\&\cup \{(!\langle U_1 \rangle ; S_1, !\langle U_2 \rangle ; S_2) \;\;\;|\;\;\;(S_1, S_2),(U_1, U_2)\in \mathfrak {R}\}\\&\cup \{(?(U_1) ; S_1, ?(U_2) ; S_2) \;\;\;|\;\;\;(S_1, S_2),(U_1, U_2)\in \mathfrak {R}\}\\&\cup \{({ \& } \{l_i: S_i\}_{i \in I},\, { \& } \{l_i: S_i'\}_{i \in I}) \;\;\;|\;\;\;\forall i\in I. (S_i, S_i')\in \mathfrak {R}\}\\&\cup \{(\oplus \{l_i: S_i\}_{i \in I},\, \oplus \{l_i: S_i'\}_{i \in I}) \;\;\;|\;\;\;\forall i\in I. (S_i, S_i')\in \mathfrak {R}\}\\&\cup \{(\mu \textsf {t}.S, S') \;\;\;|\;\;\;(S\mu \textsf {t}.S/\textsf {t},S')\in \mathfrak {R}\}\\&\cup \{(S,\mu \textsf {t}.S') \;\;\;|\;\;\;(S,S'\mu \textsf {t}.S'/\textsf {t})\in \mathfrak {R}\} \end{aligned}$$

Standard arguments ensure that *F* is monotone, thus the greatest fixed point of *F* exists. We write $S_1 \sim S_2$ if $(S_1,S_2)\in \mathfrak {R}$.

### Proof of Theorem 1 (type soundness)

As our type system is closely related to that considered by Mostrous and Yoshida [26], the proof of type soundness requires notions and properties which are instances of those already shown in [26]. We first state weakening and strengthening lemmas, which have standard proofs.

#### Lemma 8

(Weakening—Lemma C.2 in [26])

- If $\varGamma ; \varLambda ; \varDelta \vdash P \triangleright \diamond$ and $x \not \in \texttt {dom}(\varGamma ,\varLambda ,\varDelta )$ then $\varGamma \cdot x: U\!\! \rightarrow \! \diamond ; \varLambda ; \varDelta \vdash P \triangleright \diamond$

#### Lemma 9

(Strengthening—Lemmas C.3 and C.4 in [26]) We have:

- If $\varGamma \cdot x: U\!\! \rightarrow \! \diamond ; \varLambda ; \varDelta \vdash P \triangleright \diamond$ and $x \not \in \texttt {fv}(P)$ then $\varGamma ; \varLambda ; \varDelta \vdash P \triangleright \diamond$
- If $\varGamma ; \varLambda ; \varDelta \cdot s: \texttt {end}\vdash P \triangleright \diamond$ and $s \not \in \texttt {fn}(P)$ then $\varGamma ; \varLambda ; \varDelta \vdash P \triangleright \diamond$

Below, shared value means that there are no free linear names, thus $$\varLambda , \varDelta $$ are empty (cf. Rule $$ {[{{Prom}]}}$$ in Fig. 3).

#### Lemma 10

(Substitution Lemma—Lemma C.10 in [26]) We have:

1. Suppose $$\varGamma ; \varLambda ; \varDelta \cdot x:S \vdash P \triangleright \diamond $$ and $$s \not \in \texttt {dom}(\varGamma , \varLambda , \varDelta )$$.
  Then $$\varGamma ; \varLambda ; \varDelta \cdot s:S \vdash Ps/x \triangleright \diamond $$.
2. Suppose $$\varGamma \cdot x:\langle U \rangle ; \varLambda ; \varDelta \vdash P \triangleright \diamond $$ and $$a \notin \texttt {dom}(\varGamma , \varLambda , \varDelta )$$.
  Then $$\varGamma \cdot a:\langle U \rangle ; \varLambda ; \varDelta \vdash Pa/x \triangleright \diamond $$.
3. Suppose $\varGamma ; \varLambda _1 \cdot x:U\!\! \multimap \! \diamond ; \varDelta _1 \vdash P \triangleright \diamond$ and $\varGamma ; \varLambda _2; \varDelta _2 \vdash V \triangleright U\!\! \multimap \! \diamond$ with $\varLambda _1, \varLambda _2$ and $\varDelta _1, \varDelta _2$ defined. Then $\varGamma ; \varLambda _1 \cdot \varLambda _2; \varDelta _1 \cdot \varDelta _2 \vdash PV/x \triangleright \diamond$.
4. Suppose $\varGamma \cdot x:U\!\! \rightarrow \! \diamond ; \varLambda ; \varDelta \vdash P \triangleright \diamond$ and shared value *V* such that
  $$\varGamma ; \emptyset ; \emptyset \vdash V \triangleright U\!\! \rightarrow \! \diamond $$ Then $$\varGamma ; \varLambda ; \varDelta \vdash PV/x \triangleright \diamond $$.

#### Proof

In all four parts, we proceed by induction on the typing for *P*, with a case analysis on the last applied rule. $\square$

We now state the instance of type soundness that we can derive from [26]. It is worth noticing the definition of structural congruence in [26] is richer than ours. Also, their statement for subject reduction relies on an ordering on typings, associated to queues and other runtime elements. Since we are working with synchronous communication this ordering can be omitted. The second part of the following statement corresponds to Theorem 1:

#### Theorem 3

(Type soundness) We have:

1. (Subject congruence) Suppose $\varGamma ; \varLambda ; \varDelta \vdash P \triangleright \diamond$. Then $P \equiv P'$ implies $\varGamma ; \varLambda ; \varDelta \vdash P' \triangleright \diamond$.
2. (Subject reduction) Suppose $\varGamma ; \emptyset ; \varDelta \vdash P \triangleright \diamond$ with balanced $\varDelta$. Then $P \longrightarrow P'$ implies $\varGamma ; \emptyset ; \varDelta ' \vdash P' \triangleright \diamond$ and $\varDelta = \varDelta '$ or $\varDelta \longrightarrow \varDelta '$.

#### Proof

Part (1) is standard, using weakening and strengthening lemmas. Part (2) proceeds by induction on the last reduction rule used. Below, we give some details:

1. Case [App]: Then we have
  $$\begin{aligned} P = (\lambda x.\,Q) \, u \longrightarrow Q u/x = P' \end{aligned}$$
  Suppose $\varGamma ;\, \emptyset ;\, \varDelta \vdash (\lambda x.\,Q) \, u \triangleright \diamond$. We examine one possible way in which this assumption can be derived; other cases are similar or simpler:
  $$\begin{aligned} \dfrac{ \dfrac{\varGamma ;\, \emptyset ;\, \varDelta \cdot \{x:S\} \vdash Q \triangleright \diamond \quad \varGamma ';\, \emptyset ;\, \{x:S\} \vdash x \triangleright S}{ \varGamma ;\, \emptyset ;\, \varDelta \vdash \lambda x.\,Q \triangleright S\!\! \multimap \! \diamond } \qquad \dfrac{}{ \varGamma ;\, \emptyset ;\, \{u:S\} \vdash u \triangleright S} }{ \varGamma ;\, \emptyset ;\, \varDelta \cdot u:S \vdash (\lambda x.\,Q) \, u \triangleright \diamond } \end{aligned}$$
  Then, by combining premise $\varGamma ;\, \emptyset ;\, \varDelta \cdot \{x:S\} \vdash Q \triangleright \diamond$ with the substitution lemma (Lemma 10(1)), we obtain $\varGamma ;\, \emptyset ;\, \varDelta \cdot u:S \vdash Qu/x \triangleright \diamond$, as desired.
2. Case [Pass]: There are several sub-cases, depending on the type of the communication subject *n* (which could be a shared or a linear name) and the type of the object *V* (which could be an abstraction or a shared/linear name). We analyse two representative sub-cases:
  - *n* is a shared name and *V* is a name *v*. Then we have the following reduction:
    $$\begin{aligned} P = n !\langle v \rangle . Q_1 \;|\;n ?(x) . Q_2 \longrightarrow Q_1 \;|\;Q_2 v/x = P' \end{aligned}$$
    By assumption, we have the following typing derivation:
    $$\begin{aligned} \frac{(11)\quad (12)}{ \varGamma ;\, \emptyset ;\, \varDelta _1 \cdot \{v:S\} \cdot \varDelta _3 \vdash n !\langle v \rangle . Q_1 \;|\;n ?(x) . Q_2 \triangleright \diamond } \end{aligned}$$
    where (11) and (12) are as follows:
    11 $$\begin{aligned}&\dfrac{ \varGamma ' \cdot n:\langle S \rangle ;\, \emptyset ;\, \emptyset \vdash n \triangleright \langle S \rangle \quad \varGamma ;\, \emptyset ;\, \varDelta _1 \vdash Q_1 \triangleright \diamond \quad \varGamma ;\, \emptyset ;\, \{v:S\} \vdash v \triangleright S }{ \varGamma ;\, \emptyset ;\, \varDelta _1 \cdot \{v:S\} \vdash n !\langle v \rangle . Q_1 \triangleright \diamond }&\qquad \end{aligned}$$
    12 $$\begin{aligned}&\dfrac{ \varGamma ' \cdot n:\langle S \rangle ;\, \emptyset ;\, \emptyset \vdash n \triangleright \langle S \rangle \quad \varGamma ;\, \emptyset ;\, \varDelta _3 \cdot x:S \vdash Q_2 \triangleright \diamond }{ \varGamma ;\, \emptyset ;\, \varDelta _3 \vdash n ?(x) . Q_2 \triangleright \diamond }&\end{aligned}$$
    Now, by applying Lemma 10(1) on $\varGamma ;\, \emptyset ;\, \varDelta _3 \cdot x:S \vdash Q_2 \triangleright \diamond$ we obtain
    $$\begin{aligned} \varGamma ;\, \emptyset ;\, \varDelta _3 \cdot v:S \vdash Q_2v/x \triangleright \diamond \end{aligned}$$
    and the case is completed by using Rule  $[{{Par}]}$ with this judgement:
    $$\begin{aligned} \frac{ \varGamma ; \emptyset ; \varDelta _1 \vdash Q_1 \triangleright \diamond \quad \varGamma ;\, \emptyset ;\, \varDelta _3 \cdot v:S \vdash Q_2v/x \triangleright \diamond }{ \varGamma ; \emptyset ; \varDelta _1 \cdot \varDelta _3 \cdot v:S \vdash Q_1 \;|\;Q_2v/x \triangleright \diamond } \end{aligned}$$
    Observe how in this case the session environment does not reduce.
  - *n* is a shared name and *V* is a higher-order value. Then we have the following reduction:
    $$\begin{aligned} P = n !\langle V \rangle . Q_1 \;|\;n ?(x) . Q_2 \longrightarrow Q_1 \;|\;Q_2 V/x = P' \end{aligned}$$
    By assumption, we have the following typing derivation (below, we write *L* to stand for $C\!\! \rightarrow \! \diamond$ and $\varGamma$ to stand for $\varGamma ' \setminus x$).
    $$\begin{aligned} \frac{(13)\quad (14)}{ \varGamma ;\, \emptyset ;\, \varDelta _1 \cdot \varDelta _3 \vdash n !\langle v \rangle . Q_1 \;|\;n ?(x) . Q_2 \triangleright \diamond } \end{aligned}$$
    where (13) and (14) are as follows:
    13 $$\begin{aligned}&\dfrac{ \varGamma ;\, \emptyset ;\, \emptyset \vdash n \triangleright \langle L \rangle \quad \varGamma ;\, \emptyset ;\, \varDelta _1 \vdash Q_1 \triangleright \diamond \quad \varGamma ;\, \emptyset ;\, \emptyset \vdash V \triangleright L }{ \varGamma ;\, \emptyset ;\, \varDelta _1 \vdash n !\langle V \rangle . Q_1 \triangleright \diamond }&\end{aligned}$$
    14 $$\begin{aligned}&\dfrac{ \varGamma ' ;\, \emptyset ;\, \emptyset \vdash n \triangleright \langle L \rangle \quad \varGamma ';\, \emptyset ;\, \varDelta _3 \vdash Q_2 \triangleright \diamond \quad \varGamma ' ;\, \emptyset ;\, \emptyset \vdash x \triangleright L }{ \varGamma ;\, \emptyset ;\, \varDelta _3 \vdash n ?(x) . Q_2 \triangleright \diamond }&\end{aligned}$$
    Now, by applying Lemma 10(4) on $\varGamma ' \backslash x;\, \emptyset ;\, \varDelta _3 \vdash Q_2 \triangleright \diamond$ and $\varGamma ;\, \emptyset ;\, \emptyset \vdash V \triangleright L$ we obtain
    $$\begin{aligned} \varGamma ;\, \emptyset ;\, \varDelta _3 \vdash Q_2V/x \triangleright \diamond \end{aligned}$$
    and the case is completed by using Rule  $[{{Par}]}$ with this judgement:
    $$\begin{aligned} \frac{ \varGamma ; \emptyset ; \varDelta _1 \vdash Q_1 \triangleright \diamond \quad \varGamma ;\, \emptyset ;\, \varDelta _3 \vdash Q_2V/x \triangleright \diamond }{ \varGamma ; \emptyset ; \varDelta _1 \cdot \varDelta _3 \vdash Q_1 \;|\;Q_2V/x \triangleright \diamond } \end{aligned}$$
    Observe how in this case the session environment does not reduce.
3. Case [Sel]: The proof is standard, the session environment reduces.
4. Cases [Par] and [Res]: The proof is standard, exploiting induction hypothesis.
5. Case [Cong]: follows from Theorem 3 (1).

## Appendix 2: Proofs for Sect. 5

### Typability of characteristic processes

We state and prove a more detailed form of Proposition 1. The case of recursive session types requires the following two auxiliary definitions for session type unfolding and prefix deletion.

#### Definition 22

(*Session type unfolding*) Given a session type *S*, the function $\textsf {unfold}(S)$ is defined as:

$$ \begin{aligned} \begin{array}{c} \textsf {unfold}(!\langle U \rangle ; S) = !\langle U \rangle ; S \qquad \textsf {unfold}(?(U) ; S) = ?(U) ; S \\ \textsf {unfold}(\oplus \{l_i: S_i\}_{i \in I}) = \oplus \{l_i: S_i\}_{i \in I} \qquad \textsf {unfold}({ \& } \{l_i: S_i\}_{i \in I}) = { \& } \{l_i: S_i\}_{i \in I} \\ \textsf {unfold}(\mu \textsf {t}.S) = \textsf {unfold}(S \mu \textsf {t}.S/\textsf {t} ) \qquad \textsf {unfold}(\texttt {end}) = \texttt {end}\end{array} \end{aligned}$$

#### Lemma 11

Let *S* be a session type. Then $\textsf {unfold}(S) = S'$ and $S' \not = \mu \textsf {t}.S''$.

#### Proof

A straightforward induction on the syntax of *S*. $\square$

We define a relation for session type prefix deletion:

#### Definition 23

(*Session type prefix deletion*) Given a session type *S*, the set $\textsf {del}(S)$ is defined inductively as follows:

$$ \begin{aligned} \begin{array}{c} \textsf {del}(!\langle U \rangle ; S) = \{S\} \qquad \textsf {del}(?(U) ; S) = \{S\} \\ \textsf {del}(\oplus \{l_i: S_i\}_{i \in I}) = \{S_i\}_{i \in I} \qquad \textsf {del}({ \& } \{l_i: S_i\}_{i \in I}) = \{S_i\}_{i \in I} \\ \textsf {del}(\mu \textsf {t}.S) = \textsf {del}(\textsf {unfold}(\mu \textsf {t}.S)) \qquad \textsf {del}(\texttt {end}) = \{\texttt {end}\} \end{array} \end{aligned}$$

We may now finally state and prove the following proposition:

#### Proposition 4

(Characteristic processes/values inhabit their types)

1. Let *U* and $[\!\!(U)\!\!]_{\textsf {c}}$ be a type and its characteristic value, respectively.
  - If $U = S$ then, for some s, we have $\emptyset ; \emptyset ; s: S \vdash [\!\!(S)\!\!]_{\textsf {c}} \triangleright S$.
  - If $U = \langle S \rangle$ then, for some *a*, we have $a: \langle S \rangle ; \emptyset ; \emptyset \vdash [\!\!(\langle S \rangle )\!\!]_{\textsf {c}} \triangleright \langle S \rangle$.
  - If $U = \langle L \rangle$ then, for some *a*, we have $a: \langle L \rangle ; \emptyset ; \emptyset \vdash [\!\!(\langle L \rangle )\!\!]_{\textsf {c}} \triangleright \langle L \rangle$.
  - If $U = U'\!\! \rightarrow \! \diamond$ and $\varGamma ; \emptyset ; \varDelta \vdash [\!\!(U')\!\!]^{x} \triangleright \diamond$ then we have
    $$\varGamma \backslash x ; \emptyset ; \varDelta \backslash x \vdash [\!\!(U'\!\! \rightarrow \! \diamond )\!\!]_{\textsf {c}} \triangleright U'\!\! \rightarrow \! \diamond $$.
  - If $U = U'\!\! \multimap \! \diamond$ and $\varGamma ; \emptyset ; \varDelta \vdash [\!\!(U')\!\!]^{x} \triangleright \diamond$ then we have
    $$\varGamma \backslash x ; \emptyset ; \varDelta \backslash x \vdash [\!\!(U'\!\! \multimap \! \diamond )\!\!]_{\textsf {c}} \triangleright U'\!\! \multimap \! \diamond $$.
2. Let *S* and $[\!\!(S)\!\!]^{s}$ be a session type and its characteristic process, respectively.
  - If $$S = \texttt {end}$$ then $$\emptyset ; \emptyset ; \emptyset ; \vdash [\!\!(\texttt {end})\!\!]^{s} \triangleright \diamond $$.
  - If $S = !\langle U \rangle ; S'$ and $\varGamma ; \emptyset ; \varDelta \vdash [\!\!(U)\!\!]_{\textsf {c}} \triangleright U$ then
    $$\varGamma ; \emptyset ; \varDelta \cdot t: !\langle S' \rangle ; \texttt {end}\cdot s: !\langle U \rangle ; S' \vdash [\!\!(!\langle U \rangle ; S')\!\!]^{s} \triangleright \diamond $$.
  - If $S = ?(U) ; S'$ and $\varGamma ; \emptyset ; \varDelta \vdash [\!\!(U)\!\!]^{x} \triangleright \diamond$ then
    $$\varGamma \backslash x; \emptyset ; (\varDelta \backslash x) \cdot t: ?(S') ; \texttt {end}\cdot s: !\langle U \rangle ; S' \vdash [\!\!(?(U) ; S')\!\!]^{s} \triangleright \diamond $$.
  - If $$S = \oplus \{l_i: S_i\}_{i \in I}$$ then
    $$\emptyset ; \emptyset ; \{t_i: !\langle S_i \rangle ; \texttt {end}\}_{i \in I} \cdot s: \oplus \{l_i: S_i\}_{i \in I} \vdash [\!\!(\oplus \{l_i: S_i\}_{i \in I})\!\!]^{s} \triangleright \diamond $$.
  - If $$ S = { \& } \{l_i: S_i\}_{i \in I}$$ then
    $$ \emptyset ; \emptyset ; \{t_i: !\langle S_i \rangle ; \texttt {end}\}_{i \in I} \cdot s: { \& } \{l_i: S_i\}_{i \in I} \vdash [\!\!({ \& } \{l_i: S_i\}_{i \in I})\!\!]^{s} \triangleright \diamond $$.
  - If $S = \mu \textsf {t}.S'$ then either
  - $$\emptyset ; \emptyset ; \emptyset \vdash [\!\!(\mu \textsf {t}.S')\!\!]^{s} \triangleright \diamond $$
  - for all $S_i \in \textsf {del}(S)$ there exist $\varGamma , \varDelta$, and $S_i'$ such that
    $$\begin{aligned}\varGamma ; \emptyset ; \varDelta \cdot \{t_i: S_i'\}_{i \in I} \cdot s: S' \texttt {end}/\textsf {t} \vdash [\!\!(S' \texttt {end}/\textsf {t})\!\!]^{s} \triangleright \diamond \end{aligned}$$
    and $$\varGamma ; \emptyset ; \varDelta \cdot \{t_i: !\langle S_i \rangle ; \texttt {end}\}_{i \in I} \cdot s: \mu \textsf {t}.S' \vdash [\!\!(\mu \textsf {t}.S')\!\!]^{s} \triangleright \diamond $$.

3. Let *U* and $[\!\!(U)\!\!]^{a}$ be a channel type and its characteristic process, respectively.
  - If $U = \langle S \rangle$ and $\emptyset ; \emptyset ; \varDelta \vdash [\!\!(S)\!\!]_{\textsf {c}} \triangleright S$ then $a: \langle S \rangle ; \emptyset ; \varDelta \cdot t: !\langle \langle S \rangle \rangle ; \texttt {end}\vdash [\!\!(\langle S \rangle )\!\!]^{a} \triangleright \diamond$.
  - If $U = \langle L \rangle$ and $\varGamma ; \emptyset ; \varDelta \vdash [\!\!(L)\!\!]_{\textsf {c}} \triangleright L$ then $\varGamma \cdot a: \langle L \rangle ; \emptyset ; \varDelta \cdot t: !\langle \langle L \rangle \rangle ; \texttt {end}\vdash [\!\!(\langle L \rangle )\!\!]^{a} \triangleright \diamond$.
  - If $U = U'\!\! \rightarrow \! \diamond$ and $\varGamma ; \emptyset ; \varDelta \vdash [\!\!(U')\!\!]_{\textsf {c}} \triangleright U'$ then $\varGamma \cdot x: U'\!\! \rightarrow \! \diamond ; \emptyset ;\varDelta \vdash [\!\!(U'\!\! \rightarrow \! \diamond )\!\!]^{x} \triangleright \diamond$.
  - If $U = U'\!\! \multimap \! \diamond$ and $\varGamma ; \emptyset ; \varDelta \vdash [\!\!(U')\!\!]_{\textsf {c}} \triangleright U'$ then $\varGamma \cdot x: U'\!\! \rightarrow \! \diamond ; \emptyset ;\varDelta \vdash [\!\!(U'\!\! \multimap \! \diamond )\!\!]^{x} \triangleright \diamond$.

#### Proof

The proof proceeds by mutual induction on the syntax of types. We analyze the three parts separately:

1. We use the results from Parts 2 and 3 in a case analysis on the syntax of *U*.
  - Cases (a) $$U = S$$, (b) $$U = \langle S \rangle $$, and (c) $$U = \langle L \rangle $$: The proof is straightforward from Rules $$ {[{{Sess}]}}$$ and $$ {[{{Sh}]}}$$ (cf. Fig. 3).
  - Case (d) $$U = U'\!\! \rightarrow \! \diamond $$: By Parts 2 and 3 of this lemma we obtain $$\varGamma ; \emptyset ; \varDelta \vdash [\!\!(U')\!\!]^{x} \triangleright \diamond $$, which implies $$\varGamma \backslash x ; \emptyset ; \varDelta \backslash x \vdash [\!\!(U'\!\! \rightarrow \! \diamond )\!\!]_{\textsf {c}} \triangleright U'\!\! \rightarrow \! \diamond $$ by Rules $$ {[{{Abs}]}}$$ and $$ {[{{EProm}]}}$$ (cf. Fig. 3).
  - Case (e) $$U = U'\!\! \multimap \! \diamond $$: Similar, using Rule $$ {[{{Abs}]}}$$ (cf. Fig. 3).
2. The proof is by induction on the syntax of *S*. We detail some notable cases:
  - Case $S = !\langle U \rangle ; S'$: Then, by Definition 13, we have $[\!\!(S)\!\!]^{s} = s !\langle [\!\!(U)\!\!]_{\textsf {c}} \rangle . t !\langle s \rangle . \mathbf {0}$ and we may obtain the following derivation:
    $$\begin{aligned} \frac{ \begin{array}{l} \varGamma ; \emptyset ; s: S' \cdot t: !\langle S' \rangle ; \texttt {end}\triangleright t !\langle s \rangle . \mathbf {0}\triangleright \diamond \qquad \text {(Induction)} \\ \varGamma ; \emptyset ; \varDelta \vdash [\!\!(U)\!\!]_{\textsf {c}} \triangleright U \end{array} }{ \varGamma ; \emptyset ; \varDelta \cdot s: !\langle U \rangle ; S' \cdot t: !\langle S \rangle ; \texttt {end}\triangleright s !\langle [\!\!(U)\!\!]_{\textsf {c}} \rangle .t !\langle s \rangle . \mathbf {0}\triangleright \diamond } \end{aligned}$$
  - Case $S = ?(S_1) ; S_2$: Then, by Definition 13, we have $[\!\!(S)\!\!]^{s} = s ?(x) . (t !\langle s \rangle . \mathbf {0}\;|\;[\!\!(S_1)\!\!]^{x})$. and we may obtain the following derivation:
    $$\begin{aligned} \dfrac{ \dfrac{ \begin{array}{l} \varGamma ; \emptyset ; \varDelta \cdot x: S_1 \vdash [\!\!(S_1)\!\!]^{x} \triangleright \diamond \qquad \text {(Induction)} \\ \varGamma ; \emptyset ; t: !\langle S_2 \rangle ; \texttt {end}\cdot s: S_2 \vdash t !\langle s \rangle . \mathbf {0}\triangleright \diamond \end{array} }{ \varGamma ; \emptyset ; \varDelta \cdot x: S_1 \cdot t: !\langle S_2 \rangle ; \texttt {end}\cdot s: S_2 \vdash t !\langle s \rangle . \mathbf {0}\;|\;[\!\!(S_1)\!\!]^{x} \triangleright \diamond } }{ \varGamma ; \emptyset ; \varDelta \cdot t: !\langle S_2 \rangle ; \texttt {end}\cdot s: ?(U) ; S_2 \vdash s ?(x) . (t !\langle s \rangle . \mathbf {0}\;|\;[\!\!(S_1)\!\!]^{x}) \triangleright \diamond } \end{aligned}$$
  - Case $S = \mu \textsf {t}.S'$: Then, by Definition 13, $[\!\!(S)\!\!]^{=} [\!\!(S' \texttt {end}/\textsf {t} )\!\!]^{u}$. The proof is done by induction on the shape of $S'$. We detail two sub-cases; the rest is similar or simpler.
    (i) Sub-case $S' = { \& } \{l_i: S_i\}_{i \in I}$: Then $[\!\!(S' \texttt {end}/\textsf {t})\!\!]^{s} = s \triangleright \{l_i: t_i !\langle s \rangle . \mathbf {0}\}_{i \in I}$ and $\textsf {del}(S) = \{S_i\}_{i \in I}$:
      $$\begin{aligned} \frac{ \forall i \in I, \emptyset ; \emptyset ; t_i: S_i \texttt {end}/\textsf {t} \vdash t_i !\langle s \rangle . \mathbf {0}\triangleright \diamond }{ \emptyset ; \emptyset ; t_i: S_i \texttt {end}/\textsf {t} \cdot s: S' \texttt {end}/\textsf {t} \vdash s \triangleright \{l_i: t_i !\langle s \rangle . \mathbf {0}\}_{i \in I} \triangleright \diamond } \end{aligned}$$
      We may then type $[\!\!(\mu \textsf {t}. { \& } \{l_i: S_i\}_{i \in I} )\!\!]^{s}$:
      $$ \begin{aligned} \frac{ \forall i \in I, \emptyset ; \emptyset ; t_i: S_i \vdash t_i !\langle s \rangle . \mathbf {0}\triangleright \diamond }{ \emptyset ; \emptyset ; t_i: S_i \cdot s: \mu \textsf {t}. { \& } \{l_i: S_i\}_{i \in I} \vdash s \triangleright \{l_i: t_i !\langle s \rangle . \mathbf {0}\}_{i \in I} \triangleright \diamond } \end{aligned}$$
    (ii) Sub-case $S' = \mu \textsf {t'}.S''$: Then $[\!\!(\mu \textsf {t'}.S'' \texttt {end}/\textsf {t})\!\!]^{s} = [\!\!(S'' \texttt {end}/\textsf {t} \texttt {end}/\textsf {t'} )\!\!]^{s}$. If $[\!\!(S'' \texttt {end}/\textsf {t} \texttt {end}/\textsf {t'} )\!\!]^{s} = \mathbf {0}$ then the proof is straightforward. If $\textsf {del}(S) = \{S_i\}_{i \in I}$ then by induction
      $$\begin{aligned} \frac{ \text {(Induction)} }{ \varGamma ; \emptyset ; \varDelta \cdot t_i: S_i \texttt {end}/\textsf {t} \texttt {end}/\textsf {t'} \cdot s: S'' \texttt {end}/\textsf {t} \texttt {end}/\textsf {t'} \vdash [\!\!(S'' \texttt {end}/\textsf {t} \texttt {end}/\textsf {t'} )\!\!]^{s} \triangleright \diamond } \end{aligned}$$
      We may then type $[\!\!(S)\!\!]^{s}$:
      $$\begin{aligned} \frac{ \text {(Induction)} }{ \varGamma ; \emptyset ; \varDelta \cdot t_i: S_i \cdot s: S \vdash [\!\!(\mu \textsf {t}. \mu \textsf {t'}.S'')\!\!]^{s} \triangleright \diamond } \end{aligned}$$
3. The proof uses the result of Part 1. We do a case analysis on the structure of *U*.
  - Case $$U = \langle S \rangle $$: From Part 1 we have that $$\emptyset ; \emptyset ; \varDelta \vdash [\!\!(S)\!\!]_{\textsf {c}} \triangleright S$$. By applying Rule $$ {[{{Req}]}}$$ (cf. Fig. 3) we obtain:
    $$\begin{aligned} \frac{ a: \langle S \rangle ; \emptyset ; \varDelta \vdash [\!\!(S)\!\!]_{\textsf {c}} \triangleright S \qquad a: \langle S \rangle ; \emptyset ; t: !\langle \langle S \rangle \rangle ; \texttt {end}\vdash t !\langle a \rangle . \mathbf {0}\triangleright \diamond }{ a: \langle S \rangle ; \emptyset ; \varDelta \cdot t: !\langle \langle S \rangle \rangle ; \texttt {end}\vdash [\!\!(\langle S \rangle )\!\!]^{a} \triangleright \diamond } \end{aligned}$$
  - Case $U = \langle S \rangle$: Similar argumentation as in the previous case.
  - Case $$U = U'\!\! \multimap \! \diamond $$: From Part 1 we know that $$\varGamma ; \emptyset ; \varDelta \vdash [\!\!(U')\!\!]_{\textsf {c}} \triangleright U'$$. By applying Rules $$ {[{{App}]}}$$ and $$ {[{{EProm}]}}$$ (cf. Fig. 3) we obtain:
    $$\begin{aligned} \dfrac{ \dfrac{ \varGamma ; \emptyset ; \varDelta \vdash [\!\!(U')\!\!]_{\textsf {c}} \triangleright U' \qquad \varGamma ; x: U'\!\! \multimap \! \diamond ; \emptyset \vdash x \triangleright U'\!\! \multimap \! \diamond }{ \varGamma ; x: U'\!\! \multimap \! \diamond ; \varDelta \vdash x\, {[\!\!(U')\!\!]_{\textsf {c}}} \triangleright \diamond } }{ \varGamma \cdot x: U'\!\! \rightarrow \! \diamond ; \emptyset ;\varDelta \vdash [\!\!(U'\!\! \multimap \! \diamond )\!\!]^{x} \triangleright \diamond } \end{aligned}$$
  - Case $$U = U'\!\! \rightarrow \! \diamond $$: Similar argumentation as in the previous case without applying Rule $$ {[{{EProm}]}}$$ (cf. Fig. 3).$$\square $$

### Deterministic transitions

The proofs for Theorem 2 require an auxiliary result on deterministic transitions (Lemma 1). Some notions needed to prove this auxiliary result are presented next.

In the following we sometimes use polyadic abstractions (denoted $\lambda \widetilde{x}.\,P$) and polyadic name passing (denoted $u !\langle \widetilde{V} \rangle .{P}$ and $u ?(\widetilde{x}) .{P}$, respectively) as shorthand notations.

We now prove Proposition :

#### Proposition 5

($\tau$-inertness) Suppose $\varGamma ; \emptyset ; \varDelta \vdash P \triangleright \diamond$ with balanced $\varDelta$. Then

1. implies $$\varGamma ; \varDelta \vdash P \approx ^\mathtt{H} \varDelta ' \vdash P'$$.
2. implies $$\varGamma ; \varDelta \vdash P \approx ^\mathtt{H} \varDelta ' \vdash P'$$.

#### Proof

We only prove Part 1; the proof for Part 2 follows straightforwardly. The proof proceeds by showing that the relation

is a higher-order bisimulation.

Suppose first that , for some $P''$; we have to show that $P'$ can produce an appropriate matching action. There are two main cases: $\ell \ne \tau$ (a visible action) and $\ell = \tau$ (an unobservable, possibly deterministic action).

1. The first case follows easily by typing conditions and type soundness, which ensure that $P'$ has the same potential as *P* for performing visible actions.
2. The second case can be divided into two sub-cases: first, if $\tau = \tau _{\textsf {d}}$ then $P' = P''$ and the thesis trivially follows; second, if $\tau \ne \tau _{\textsf {d}}$ (i.e., *P* has the possibility of performing both $\tau _{\textsf {d}}$ and some other $\tau$) then either $P'$ has the same $\tau$ or $P'$ does not have it, because $\tau _{\textsf {d}}$ excluded the occurrence of $\tau$. The thesis follows by noticing that, in the first case, $P'$ can match the move from *P*; the second case cannot occur because of typing and the definition of deterministic transitions.

Suppose now that , for some $P''$. This case follows immediately by noticing that *P* can always match action $\ell$ by performing the deterministic action $\tau _{\textsf {d}}$ first, i.e., we can always have . This concludes the proof. $\square$

### Proof of Theorem 2

We split the proof of Theorem 2 into several lemmas:

- Lemma 12 establishes useful properties of characteristic and higher-order processes, including a two-way connection between higher-order trigger processes and an alternative trigger process (denoted $t \leftharpoonup _\texttt {A} V$, defined below).
- Lemma 13 establishes the equivalence between characteristic and higher-order trigger processes.
- Lemma 14 establishes $$\approx ^\mathtt{H}\ =\ \approx ^\mathtt{C}$$.
- Lemma 16 establishes a trigger substitution lemma (Lemma 4 in the main text), using Lemma 15.
- Lemma 18 exploits the process substitution result given by Lemma 17 (Lemma 3 in the main text) to prove that $\approx ^\mathtt{H}\ \subseteq \ \approx$.
- Lemma 19 shows that $\approx$ is a congruence which implies $\approx \ \subseteq \ \cong$.
- Lemma 22 shows that $\cong \ \subseteq \ \approx ^\mathtt{H}$ using Lemma 20 (definability) and Lemma 21 (extrusion).

We introduce a useful notation for action labels, which will be used in the following to represent matching actions.

#### Definition 24

Let $\ell$ be an action label (cf. Sect. 5.1). We define the action $\breve{\ell }$ as

$$\begin{aligned} \breve{\ell } = \left\{ \begin{array}{lcl} (\nu \, \widetilde{m_2})(n !\langle V_2 \rangle ) &{} \quad &{} \hbox { if } \ell = (\nu \, \widetilde{m_1})(n !\langle V_1 \rangle ) \hbox {, for some} V_2, \widetilde{m_2} \\ \ell &{}&{} \text {otherwise} \end{array} \right. \end{aligned}$$

Thus, given $\ell$, its corresponding action $\breve{\ell }$ is either identical to $\ell$, or an output on the same name, possibly with different object and extruded names.

We now introduce an alternative trigger process that is used to simplify the proofs. Let

15 $$\begin{aligned} t \leftharpoonup _\texttt {A} V = t ?(x) . (\nu \, s)(x\, {s} \;|\;\overline{s} !\langle V \rangle . \mathbf {0}) \end{aligned}$$

The simpler formulation of alternative trigger process (with respect to the higher-order trigger process, cf. (6)) is useful in proofs. However, the input of characteristic values on name *t* results in the creation of redundant parallel components:

Indeed, processes of the form $(\nu \, s){t' !\langle s \rangle . \mathbf {0}}$ are redundant because the restricted name *s* has no interactions. The following lemma shows that we can ignore these processes (up to $\approx ^\mathtt{H}$ and $\approx ^\mathtt{C}$). It also states the equivalence (up to $\approx ^\mathtt{H}$) between higher-order trigger processes $t \hookleftarrow _{\texttt {H}} V$ (cf. (6)) and $t \leftharpoonup _\texttt {A} V$.

#### Lemma 12

(Auxiliary results for trigger processes) Let *P* and *Q* be processes.

1. Let *t* be a fresh name, $\varDelta _1 = \varDelta _3 \cdot t: !\langle \texttt {end} \rangle ; \texttt {end}$, and $\varDelta _2 = \varDelta _4 \cdot t: !\langle \texttt {end} \rangle ; \texttt {end}$. Then
  $$\begin{aligned} \varGamma ; \varDelta _1 \vdash (\nu \, \widetilde{m_1})(P \;|\;(\nu \, s)(t !\langle s \rangle . \mathbf {0}) ) \approx ^\mathtt{H} \varDelta _2 \vdash (\nu \, \widetilde{m_2})(Q \;|\;(\nu \, s)(t !\langle s \rangle . \mathbf {0}) ) \end{aligned}$$
  if and only if
  $$\begin{aligned} \varGamma ; \varDelta _3 \vdash (\nu \, \widetilde{m_1})P \approx ^\mathtt{H} \varDelta _4 \vdash (\nu \, \widetilde{m_2})Q \end{aligned}$$
2. Let *t* a fresh name, $\varDelta _1 = \varDelta _3 \cdot t: !\langle \texttt {end} \rangle ; \texttt {end}$ and $\varDelta _2 = \varDelta _4 \cdot t: !\langle \texttt {end} \rangle ; \texttt {end}$. Then
  $$\begin{aligned} \varGamma ; \varDelta _1 \vdash (\nu \, \widetilde{m_1})(P \;|\;(\nu \, s)(t !\langle s \rangle . \mathbf {0}) ) \approx ^\mathtt{C} \varDelta _2 \vdash (\nu \, \widetilde{m_2})(Q \;|\;(\nu \, s)(t !\langle s \rangle . \mathbf {0}) ) \end{aligned}$$
  if and only if
  $$\begin{aligned} \varGamma ; \varDelta _3 \vdash (\nu \, \widetilde{m_1})P \approx ^\mathtt{C} \varDelta _4 \vdash (\nu \, \widetilde{m_2})Q \end{aligned}$$
3. Let *t* be a fresh name. Then
  $$\begin{aligned} \varGamma ; \varDelta _1 \vdash (\nu \, \widetilde{m_1})(P \;|\;t \leftharpoonup _\texttt {A} V_1 ) \approx ^\mathtt{H} \varDelta _2 \vdash (\nu \, \widetilde{m_2})(Q \;|\;t \leftharpoonup _\texttt {A} V_2 ) \end{aligned}$$
  if and only if, for some $\varDelta _3, \varDelta _4$,
  $$\begin{aligned} \varGamma ; \varDelta _3 \vdash (\nu \, \widetilde{m_1})(P \;|\;t \hookleftarrow _{\texttt {H}} V_1 ) \approx ^\mathtt{H} \varDelta _4 \vdash (\nu \, \widetilde{m_2})(Q \;|\;t \hookleftarrow _{\texttt {H}} V_2) \end{aligned}$$

#### Proof

We analyze each of the three parts:

- Part 1. We split the proof into the two directions of the if and only if requirements.

- First direction. Consider the typed relation (we omit the type information):
  We check the requirements of higher-order bisimulation for $\mathfrak {R}$. Suppose that
  then we need to show a matching action from $(\nu \, \widetilde{m_2})Q$. We can derive that
  for some $\varDelta _1'$ which, from the freshness of *t*, implies that there exist $Q'$ and $\varDelta _2'$ such that
  16
  and
  $$\begin{aligned} \varGamma ; \varDelta _1' \vdash (\nu \, \widetilde{m_1}')(P' \;|\;C_1 \;|\;(\nu \, s)(t !\langle s \rangle . \mathbf {0}) ) \approx ^\mathtt{H} \varDelta _2' \vdash (\nu \, \widetilde{m_2}')(Q' \;|\;C_2 \;|\;(\nu \, s)(t !\langle s \rangle . \mathbf {0}) ) \end{aligned}$$
  where the shape of $C_1, C_2$ depends on $\ell$ and $\breve{\ell }$: if they are output actions with objects $V_1$ and $V_2$, respectively, then $C_1 = t' \hookleftarrow _{\texttt {H}} V_1$ and $C_2 = t' \hookleftarrow _{\texttt {H}} V_2$; otherwise, $C_1 = C_2 = \mathbf {0}$. The latter equation implies from the definition of $\mathfrak {R}$
  $$\begin{aligned} \varGamma ; \varDelta _1' \vdash (\nu \, \widetilde{m_1}')(P' \;|\;C_1 \;|\;(\nu \, s)(t !\langle s \rangle . \mathbf {0}) ) \ \mathfrak {R}\ \varDelta _2' \vdash (\nu \, \widetilde{m_2}')(Q' \;|\;C_2 \;|\;(\nu \, s)(t !\langle s \rangle . \mathbf {0}) ) \end{aligned}$$
  and (16) implies
  to complete the proof of the case.
- Second direction. Consider the typed relation (we omit the type information):
  $$\begin{aligned} \mathfrak {R}= & {} \{ ((\nu \, \widetilde{m_1})(P \;|\;(\nu \, s)(t !\langle s \rangle . \mathbf {0}))\ , \ (\nu \, \widetilde{m_2})(Q \;|\;(\nu \, s)(t !\langle s \rangle . \mathbf {0}))) \ \ |\ \ \varGamma ;\\&\quad \varDelta _3 \vdash (\nu \, \widetilde{m_1})(P ) \approx ^\mathtt{H} \varDelta _4 \vdash (\nu \, \widetilde{m_2})(Q ) \} \end{aligned}$$
  We check the requirements of higher-order bisimulation for $\mathfrak {R}$. Suppose that $(\nu \, \widetilde{m_1})(P \;|\;(\nu \, s)(t !\langle s \rangle . \mathbf {0}))$ moves; we need to infer an appropriate matching action from $(\nu \, \widetilde{m_2})(Q \;|\;(\nu \, s)(t !\langle s \rangle . \mathbf {0}))$. We analyse three cases:
  (i) Process *P* moves autonomously, i.e., for some $\varDelta _1'$ we have:
    Then the proof is similar to the previous case.
  (ii) An action on the fresh name *t*, i.e., for some $\varDelta _1'$ we have:
    First notice that the typing derivation reveals that $\varDelta _1(t) = \varDelta _2(t) = !\langle \texttt {end} \rangle ; \texttt {end}$. This is because the dual endpoint of the (restricted) session *s* does not appear in $(\nu \, s)(t !\langle s \rangle . \mathbf {0})$ and thus it has the inactive type $\texttt {end}$. We can then observe that, for some $\varDelta _2'$, we have:
    We need to show that
    $$\begin{aligned} \varGamma ; \varDelta _1' \vdash (\nu \, \widetilde{m_1})(P \;|\;t' \hookleftarrow _{\texttt {H}} s ) \approx ^\mathtt{H} \varDelta _2' \vdash (\nu \, \widetilde{m_2})(Q' \;|\;t' \hookleftarrow _{\texttt {H}} s ) \end{aligned}$$
    The proof is easy if we consider that both processes can perform the up-to deterministic transitions :
    and
    The result is then immediate from the definition of $\mathfrak {R}$ that requires
    $$\begin{aligned} \varGamma ; \varDelta _1' \vdash (\nu \, \widetilde{m_1}){P } \approx ^\mathtt{H} \varDelta _2' \vdash (\nu \, \widetilde{m_2}){Q' } \end{aligned}$$
  (iii) A synchronization along name *t*: this is not possible due to the freshness of *t*.

This concludes the proof of Part 1.

- Part 2 follows same arguments and structure as the proof for Part 1.
- Part 3 relies on Part 1. We analyse the two directions of the if and only if requirement.

- First direction. Let $\mathfrak {R}$ be the typed relation (we omit the type information):
  $$\begin{aligned} \mathfrak {R}= & {} \{ ((\nu \, \widetilde{m_1})(P \;|\;t \hookleftarrow _{\texttt {H}} V_1)\ ,\ (\nu \, \widetilde{m_2})(Q \;|\;t \hookleftarrow _{\texttt {H}} V_2)) \ \ |\ \ \\&\qquad \varGamma ; \varDelta _1 \vdash (\nu \, \widetilde{m_1})(P \;|\;t \leftharpoonup _\texttt {A} V_1 ) \approx ^\mathtt{H} \varDelta _2 \vdash (\nu \, \widetilde{m_2})(Q \;|\;t \leftharpoonup _\texttt {A} V_2 ) \} \end{aligned}$$
  We show that $\mathfrak {R}\ \subseteq \ \approx ^\mathtt{H}$, with a case analysis on the defining requirements of higher-order bisimulation. Suppose that $(\nu \, \widetilde{m_1})(P \;|\;t \hookleftarrow _{\texttt {H}} V_1)$ moves; we need to show an appropriate matching action from $(\nu \, \widetilde{m_2})(Q \;|\;t \hookleftarrow _{\texttt {H}} V_2)$. We analyze three possibilities:
  (i) *P* moves on its own, i.e., for some $\varDelta '_1$ we have:
    The proof is similar to case (a) of Part 1 of this lemma.
  (ii) An input action of the form $t ?\langle n \rangle$ along a fresh name *t*. Let *U* be such that $[\!\!(U)\!\!]_{\textsf {c}} = n$ and let $V_1$ be a higher-order value. There exists a $\varDelta '_1$ such that:
    Furthermore, we can see that, for some $\varDelta '_2$, we have
    We therefore need to show that
    $$\begin{aligned} \varGamma ; \varDelta _1' \vdash (\nu \, \widetilde{m_1}')(P \;|\;(V_1\, {n})) \approx ^\mathtt{H} \varDelta _2' \vdash (\nu \, \widetilde{m_2}')(Q' \;|\;(V_2\, {n})) \end{aligned}$$
    This is done by considering the requirements of $\mathfrak {R}$.
    Because of the definition of the alternative trigger, the input of the trigger value has no effect on the bisimulation relation:
    Since $[\!\!(U)\!\!]_{\textsf {c}} = n$, we have that $[\!\!((?(U) ; \texttt {end})\!\! \rightarrow \! \diamond )\!\!]_{\textsf {c}} = \lambda z.\, z ?(y) . ( t' !\langle z \rangle . \mathbf {0}\;|\;(y\, {n}) )$:
    Furthermore, we can see that
    We also have
    and so we can infer from the up-to technique for deterministic transitions (Lemma 1) that
    $$\begin{aligned} \varGamma ; \varDelta _1' \vdash (\nu \, \widetilde{m_1}')(P \;|\;(V_1\, {n}) \;|\;(\nu \, s)(t' !\langle s \rangle . \mathbf {0}))\\ \approx ^\mathtt{H} \varDelta _2' \vdash (\nu \, \widetilde{m_2}')(Q' \;|\;(V_2\, {n}) \;|\;(\nu \, s)(t' !\langle s \rangle . \mathbf {0})) \end{aligned}$$
    which implies, by Part 1 of this lemma, the desired conclusion:
    $$\begin{aligned} \varGamma ; \varDelta _1' \vdash (\nu \, \widetilde{m_1}')(P \;|\;(V_1\, {n})) \approx ^\mathtt{H} \varDelta _2' \vdash (\nu \, \widetilde{m_2}')(Q' \;|\;(V_2\, {n}) ) \end{aligned}$$
  (iii) An action of the form $t ?\langle \lambda z.\,[\!\!(U')\!\!]^{z} \rangle$ along the fresh name *t*. Let *U* such that $[\!\!(U)\!\!]_{\textsf {c}} = \lambda z.\,[\!\!(U')\!\!]^{z}$. There exist *U* and $\varDelta _1'$ such that
    Furthermore, we have the following transition, for some $\varDelta '_2$:
    We need to show that, for some $\varDelta '_3, \varDelta '_4$, the following holds:
    $$\begin{aligned} \varGamma ; \varDelta _3' \vdash (\nu \, \widetilde{m_1}')(P \;|\;(\lambda z.\,[\!\!(U')\!\!]^{z})\, {V_1}) \approx ^\mathtt{H} \varDelta _4' \vdash (\nu \, \widetilde{m_2}')(Q' \;|\;(\lambda z.\,[\!\!(U')\!\!]^{z})\, {V_2}) \end{aligned}$$
    This is done by considering the requirements of $\mathfrak {R}$.
    Here again note that the input of the trigger value has no effect on the bisimulation relation.
    We now consider the input of the characteristic value on *t*. From the fact that $[\!\!(U)\!\!]_{\textsf {c}} = \lambda z.\,[\!\!(U')\!\!]^{z}$ we obtain that $[\!\!((?(U) ; \texttt {end})\!\! \rightarrow \! \diamond )\!\!]_{\textsf {c}} = \lambda w.\, w ?(y) . ( t' !\langle w \rangle . \mathbf {0}\;|\;(\lambda z.\,[\!\!(U')\!\!]^{z})\, {y} )$ and
    Furthermore, we have the following transition, for some $\varDelta '_2$:
    We also have
    and so we can infer from the up-to technique for deterministic transitions (Lemma 1) that
    $$\begin{aligned} \begin{array}{c} \varGamma ; \varDelta _1' \vdash (\nu \, \widetilde{m_1}')(P \;|\;(\lambda z.\,[\!\!(U')\!\!]^{z})\, {V_1} \;|\;(\nu \, s)(t' !\langle s \rangle . \mathbf {0})) \approx ^\mathtt{H} \\ \vdash \varDelta _2'{(\nu \, \widetilde{m_2}')(Q' \;|\;(\lambda z.\,[\!\!(U')\!\!]^{z})\, {V_2} \;|\;(\nu \, s)(t' !\langle s \rangle . \mathbf {0}))} \end{array} \end{aligned}$$
    which implies, by Part 1 of this lemma, the desired conclusion:
    $$\begin{aligned} \varGamma ; \varDelta _1' \vdash (\nu \, \widetilde{m_1}')(P \;|\;(\lambda z.\,[\!\!(U')\!\!]^{z})\, {V_1}) \approx ^\mathtt{H} \varDelta _2' \vdash (\nu \, \widetilde{m_2}')(Q' \;|\;(\lambda z.\,[\!\!(U')\!\!]^{z})\, {V_2}) \end{aligned}$$
- Second direction. Let $\mathfrak {R}$ be the typed relation (we omit the type information):
  $$\begin{aligned} \mathfrak {R}= & {} \{ ((\nu \, \widetilde{m_1})(P \;|\;t \leftharpoonup _\texttt {A} V_1)\ ,\ (\nu \, \widetilde{m_2})(Q \;|\;t \leftharpoonup _\texttt {A} V_2)) \ \ |\ \ \\&\qquad \qquad \qquad \varGamma ; \varDelta _3 \vdash (\nu \, \widetilde{m_1})(P \;|\;t \hookleftarrow _{\texttt {H}} V_1 ) \approx ^\mathtt{H} \varDelta _4 \vdash (\nu \, \widetilde{m_2})(Q \;|\;t \hookleftarrow _{\texttt {H}} V_2 ) \} \end{aligned}$$
  We show that $\mathfrak {R}\ \subseteq \ \approx ^\mathtt{H}$, with a case analysis on the defining requirements of higher-order bisimulation. We focus on the cases related to an input action on the fresh name *t*; other cases are similar.
  i. Value $V_1$ is a higher-order value: This implies that there exist *U* and $\varDelta '_1$ such that $[\!\!(?(U) ; \texttt {end})\!\!]_{\textsf {c}} = \lambda z.\, z ?(y) . (t' !\langle z \rangle . \mathbf {0}\;|\;y\, {n})$ and
    and
    Furthermore we can see that there exists $\varDelta _2'$ such that
    We need to show that
    $$\begin{aligned} \varGamma ; \varDelta _1' \vdash (\nu \, \widetilde{m_1}')(P \;|\;(V_1\, {n}) \;|\;(\nu \, s)(t' !\langle s \rangle . \mathbf {0}))\\ \quad \approx ^\mathtt{H} \varDelta _2' \vdash (\nu \, \widetilde{m_2}')(Q \;|\;(V_2\, {n}) \;|\;(\nu \, s)(t' !\langle s \rangle . \mathbf {0})) \end{aligned}$$
    This is done by considering the requirements of $\mathfrak {R}$. We know that $[\!\!(U)\!\!]_{\textsf {c}} = n$:
    for some $\varDelta '_3$. Furthermore we can see that for some $\varDelta '_4$
    and
    $$\begin{aligned} \varGamma ; \varDelta _3' \vdash (\nu \, \widetilde{m_1}')(P \;|\;(V_1\, {n})) \approx ^\mathtt{H} \varDelta _4' \vdash (\nu \, \widetilde{m_2}')(Q' \;|\;(V_2\, {n})) \end{aligned}$$
    which imply, by Part 1 of this lemma, the desired conclusion:
    $$\begin{aligned} \varGamma ; \varDelta _3' \vdash (\nu \, \widetilde{m_1}')(P \;|\;(V_1\, {n}) \;|\;(\nu \, s)(t' !\langle s \rangle . \mathbf {0}))\\ \quad \approx ^\mathtt{H} \varDelta _4' \vdash (\nu \, \widetilde{m_2}')(Q' \;|\;(V_2\, {n}) \;|\;(\nu \, s)(t' !\langle s \rangle . \mathbf {0})) \end{aligned}$$
  ii Value $V_1$ is a first-order value: This implies that there exist *U* and $\varDelta '_3$ such that $[\!\!(?(U) ; \texttt {end})\!\!]_{\textsf {c}} = \lambda w.\, w ?(y) . ( t' !\langle w \rangle . \mathbf {0}\;|\;\lambda z.\,[\!\!(U')\!\!]^{z}\, {y} )$ and
    This case follows a similar proof structure as the previous case.
  This concludes the proof of Part 3.$\square$

The next lemma states the equivalence between the characteristic and higher-order trigger processes (cf. (6) and (7)).

#### Lemma 13

(Trigger process equivalence) Let *P* and *Q* be processes, *t* be a fresh name, and let $\varGamma ; \emptyset ; \varDelta \vdash V_i \triangleright U, i \in \{1, 2\}$.

1. If
  $$\begin{aligned} \varGamma ; \varDelta _1 \vdash (\nu \, \widetilde{m_1})(P \;|\;t \hookleftarrow _{\texttt {H}} V_1) \approx ^\mathtt{H} \varDelta _2 \vdash (\nu \, \widetilde{m_2})(Q \;|\;t \hookleftarrow _{\texttt {H}} V_2 ) \end{aligned}$$
  then there exist $\varDelta _1', \varDelta _2'$ such that
  $$\begin{aligned} \varGamma ; \varDelta _1' \vdash (\nu \, \widetilde{m_1})(P \;|\;t \Leftarrow _{\texttt {C}} V_1{\,:\,}U) \approx ^\mathtt{H} \varDelta _2' \vdash (\nu \, \widetilde{m_2})(Q \;|\;t \Leftarrow _{\texttt {C}} V_2{\,:\,}U). \end{aligned}$$
2. If
  $$\begin{aligned} \varGamma ; \varDelta _1 \vdash (\nu \, \widetilde{m_1})(P \;|\;t \Leftarrow _{\texttt {C}} V_1{\,:\,}U) \approx ^\mathtt{C} \varDelta _2 \vdash (\nu \, \widetilde{m_2})(Q \;|\;t \Leftarrow _{\texttt {C}} V_2{\,:\,}U) \end{aligned}$$
  then there exist $\varDelta _1', \varDelta _2'$ such that
  $$\begin{aligned} \varGamma ; \varDelta _1' \vdash (\nu \, \widetilde{m_1})(P \;|\;t \hookleftarrow _{\texttt {H}} V_1) \approx ^\mathtt{C} \varDelta _2' \vdash (\nu \, \widetilde{m_2})(Q \;|\;t \hookleftarrow _{\texttt {H}} V_2 ). \end{aligned}$$

#### Proof

We analyse both parts separately:

1. Consider the typed relation (for readability, we omit type information):
  $$\begin{aligned} \mathfrak {R}= & {} \{ ((\nu \, \widetilde{m_1})(P \;|\;t \Leftarrow _{\texttt {C}} V_1{\,:\,}U ), (\nu \, \widetilde{m_2})(Q \;|\;t \Leftarrow _{\texttt {C}} V_2{\,:\,}U)) \ \ |\ \ \\&\qquad \varGamma ; \varDelta _1' \vdash (\nu \, \widetilde{m_1})(P \;|\;t \hookleftarrow _{\texttt {H}} V_1) \approx ^\mathtt{H} \varDelta _2' \vdash (\nu \, \widetilde{m_2})(Q \;|\;t \hookleftarrow _{\texttt {H}} V_2) \} \end{aligned}$$
  We show that $\mathfrak {R}\ \subseteq \ \approx ^\mathtt{H}$. Suppose that $(\nu \, \widetilde{m_1})(P \;|\;t \Leftarrow _{\texttt {C}} V_1{\,:\,}U )$ moves; we need to find a matching move from $(\nu \, \widetilde{m_2})(Q \;|\;t \Leftarrow _{\texttt {C}} V_2{\,:\,}U )$. We distinguish three cases, depending on the source/kind of visible action:
  - *P* moves autonomously, i.e., for some $\varDelta _3$ we have:
    We follow the requirements of $\mathfrak {R}$ and the freshness of *t* to conclude that there exists a $\varDelta _1''$ such that
    which implies, from the higher-order bisimilarity requirement of $\mathfrak {R}$ and the freshness of *t*, that there exist $Q'$ and $\varDelta _2''$ such that
    17
    and, for some $\varDelta _1'''$ and $\varDelta _2'''$, that
    18 $$\begin{aligned} \varGamma ; \varDelta _1''' \vdash (\nu \, \widetilde{m_1}'')(P' \;|\;t \hookleftarrow _{\texttt {H}} V_1 \;|\;C_1) \approx ^\mathtt{H} \varDelta _2''' \vdash (\nu \, \widetilde{m_2}'')(Q' \;|\;t \hookleftarrow _{\texttt {H}} V_2 \;|\;C_2)\nonumber \\ \end{aligned}$$
    where the shape of $C_1, C_2$ depends on $\ell$ and $\breve{\ell }$: if they are output actions with objects $V'_1$ and $V'_2$, respectively, then $C_1 = t' \hookleftarrow _{\texttt {H}} V'_1$ and $C_2 = t' \hookleftarrow _{\texttt {H}} V'_2$; otherwise, $C_1 = C_2 = \mathbf {0}$. From (17) and the definition of $\mathfrak {R}$ we can conclude that there exists a $\varDelta _4$ such that
    Equation (18) then allows us to infer the required conclusion, for some $\varDelta _3', \varDelta _4'$:
    $$\begin{aligned} \varGamma ; \varDelta _3' \vdash (\nu \, \widetilde{m_1}''')(P' \;|\;t \Leftarrow _{\texttt {C}} V_1{\,:\,}U \;|\;C_1) \ \mathfrak {R}\ \varDelta _4' \vdash (\nu \, \widetilde{m_2}''')(Q' \;|\;t \Leftarrow _{\texttt {C}} V_2{\,:\,}U \;|\;C_2) \end{aligned}$$
  - $t \Leftarrow _{\texttt {C}} V_1{\,:\,}U$ moves autonomously, i.e., for some $\varDelta _3$ we have:
    Following requirements of $\mathfrak {R}$ and the freshness of *t* we can infer that there exists a $\varDelta _1''$ such that
    which implies, from the higher-order bisimilarity requirement of $\mathfrak {R}$ and the freshness of *t*, that there exist $Q'$ and $\varDelta _2''$ such that
    19
    and
    20 $$\begin{aligned} {\varGamma }{\varDelta _1''}{(\nu \, \widetilde{m_1})(P \;|\;(\nu \, s)(s ?(y) . [\!\!(U)\!\!]^{y} \;|\;\overline{s} !\langle V_1 \rangle . \mathbf {0}))} \,{\approx ^\mathtt{H}}\, {\varDelta _2''}{(\nu \, \widetilde{m_2})Q'} \end{aligned}$$
    The freshness of *t* allows us to mimic the transitions in (19); for some $\varDelta _4$ we obtain:
    The conclusion is immediate from (20).
  - The action comes from the interaction of *P* and $t \hookleftarrow _{\texttt {H}} V_1$: This case is not possible, due to the freshness of *t*.
2. Consider the typed relation (for readability, we omit type information):
  $$\begin{aligned} \mathfrak {R}'= & {} \{ ((\nu \, \widetilde{m_1})(P \;|\;t \hookleftarrow _{\texttt {H}} V_1), (\nu \, \widetilde{m_2})(Q \;|\;t \hookleftarrow _{\texttt {H}} V_2)) \ \ |\ \ \\&\qquad \varGamma ; \varDelta _1' \vdash (\nu \, \widetilde{m_1})(P \;|\;t \Leftarrow _{\texttt {C}} V_1{\,:\,}U) \approx ^\mathtt{C} \varDelta _2' \vdash (\nu \, \widetilde{m_2})(Q \;|\;t \Leftarrow _{\texttt {C}} V_2{\,:\,}U)\} \end{aligned}$$
  To prove that $\mathfrak {R}'\ \subseteq \ \approx ^\mathtt{C}$ we first consider relation $\mathfrak {R}$ which uses the alternative trigger in (15) (for readability, we omit type information):
  $$\begin{aligned} \mathfrak {R}= & {} \{ ((\nu \, \widetilde{m_1})(P \;|\;t \leftharpoonup _\texttt {A} V_1), (\nu \, \widetilde{m_2})(Q \;|\;t \leftharpoonup _\texttt {A} V_2)) \ \ |\ \ \\&\qquad \varGamma ; \varDelta _1' \vdash (\nu \, \widetilde{m_1})(P \;|\;t \Leftarrow _{\texttt {C}} V_1{\,:\,}U) \approx ^\mathtt{C} \varDelta _2' \vdash (\nu \, \widetilde{m_2})(Q \;|\;t \Leftarrow _{\texttt {C}} V_2{\,:\,}U)\} \end{aligned}$$
  By proving that $\mathfrak {R}\ \subseteq \ \approx ^\mathtt{C}$ we can apply Lemma 12 (Part 3), to obtain that $\mathfrak {R}'\ \subseteq \ \approx ^\mathtt{C}$. Suppose that $(\nu \, \widetilde{m_1})(P \;|\;t \leftharpoonup _\texttt {A} V_1)$ moves; we must exhibit a matching move from $(\nu \, \widetilde{m_2})(Q \;|\;t \leftharpoonup _\texttt {A} V_2)$. We distinguish four cases, depending on the source/kind of visible action:
  - *P* moves autonomously, i.e., for some $\varDelta _3$ we have:
    Then, following the requirements of $\mathfrak {R}$ and the freshness of *t*, we infer that there exists a $\varDelta _1''$ such that
    which implies, from the characteristic bisimilarity requirement of $\mathfrak {R}$ and the freshness of *t*, that there exist $Q'$ and $\varDelta _2''$ such that
    21
    and
    22 $$\begin{aligned} {\varGamma }{\varDelta _1'''}{(\nu \, \widetilde{m_1}'')(P' \;|\;t \Leftarrow _{\texttt {C}} V_1{\,:\,}U \;|\;C_1)}\, {\approx ^\mathtt{C}} {\varDelta _2'''}{(\nu \, \widetilde{m_2}'')(Q' \;|\;t \Leftarrow _{\texttt {C}} V_2{\,:\,}U \;|\;C_2)} \nonumber \\ \end{aligned}$$
    with $C_1$ (resp., $C_2$) being the characteristic trigger process in the cases where $\ell = (\nu \, \widetilde{m}) n !\langle V_1' \rangle$ (resp., $\breve{\ell } = (\nu \, \widetilde{m}') n !\langle V_2' \rangle$), and $C_1 = C_2 = \mathbf {0}$ otherwise. From (21) we can infer that there exists $\varDelta _4$ such that
    Equation (22) then allows us to obtain the desired conclusion:
    $$\begin{aligned} \varGamma ; \varDelta _3' \vdash (\nu \, \widetilde{m_1}''')(P' \;|\;t \leftharpoonup _\texttt {A} V_1 \;|\;C_1) \ \mathfrak {R}\ \varDelta _4' \vdash {(\nu \, \widetilde{m_2}''')(Q' \;|\;t \leftharpoonup _\texttt {A} V_2 \;|\;C_2)} \end{aligned}$$
  - $t \leftharpoonup _\texttt {A} V_1$ moves autonomously due to the input of characteristic value, i.e., for some $\varDelta _3$ we have:
    Following requirements of $\mathfrak {R}$ and the freshness of *t*, we infer that there is a $\varDelta _1''$ such that
    which implies, from the characteristic bisimulation requirement of $\mathfrak {R}$ and the freshness of *t*, that there exist $Q'$ and $\varDelta _2''$ such that
    23
    and
    $$\begin{aligned} \varGamma ; \varDelta _1'' \vdash (\nu \, \widetilde{m_1})P' \approx ^\mathtt{C} \varDelta _2'' \vdash (\nu \, \widetilde{m_2})Q' \end{aligned}$$
    which in turn implies from Lemma 12 (Part 2) the following, for a fresh $t'$:
    24 $$\begin{aligned} \varGamma ; \varDelta _1'' \vdash (\nu \, \widetilde{m_1})(P' \;|\;(\nu \, s)(t' !\langle s \rangle . \mathbf {0}) ) \approx ^\mathtt{C} \varDelta _2'' \vdash (\nu \, \widetilde{m_2})(Q') \;|\;(\nu \, s)(t' !\langle s \rangle . \mathbf {0}) \quad \quad \end{aligned}$$
    The freshness of *t* allows us to mimic the transitions in (23) to infer that, for some $\varDelta _4$, we have
    and
    The conclusion is immediate from (24).
  - $t \leftharpoonup _\texttt {A} V_1$ moves autonomously due to the input of a trigger process, i.e., for some $\varDelta _3$ we have:
    We show that there exist $\varDelta _4$ and $(\nu \, \widetilde{m_1})(Q \;|\;(\nu \, s)( (\lambda x.\,t' ?(y) .{(y\, {x})}){s} \;|\;\overline{s} !\langle V_2 \rangle . \mathbf {0}\, {)})$ such that
    and
    The result
    $$\begin{aligned} \varGamma ; \varDelta _3 \vdash (\nu \, \widetilde{m_1})(P \;|\;t' \leftharpoonup _\texttt {A} V_1) \ \mathfrak {R}\ \varDelta _4 \vdash (\nu \, \widetilde{m_1})(Q \;|\;t' \leftharpoonup _\texttt {A} V_2) \end{aligned}$$
    is immediate from the definition of $\mathfrak {R}$.
  - The action comes from the interaction of *P* and $t \leftharpoonup _\texttt {A} V_1$: This case is not possible, due to the freshness of *t*.$\square$

#### Lemma 14

$$\approx ^\mathtt{H}\ =\ \approx ^\mathtt{C}$$.

#### Proof

We split the proof into two parts: the direction $\approx ^\mathtt{H}\ \subseteq \ \approx ^\mathtt{C}$ and the direction $\approx ^\mathtt{C}\ \subseteq \ \approx ^\mathtt{H}$. Since the two equivalences differ only in the output case, our analysis focuses on output actions.

1. Direction $\approx ^\mathtt{H}\ \subseteq \ \approx ^\mathtt{C}$. Consider the typed relation (for readability, we omit type information):
  $$\begin{aligned} \mathfrak {R}= \{ (P, Q) \ \ |\ \ \varGamma ; \varDelta _1 \vdash P \approx ^\mathtt{H} \varDelta _2 \vdash Q\} \end{aligned}$$
  We show that $\mathfrak {R}$ is a characteristic bisimulation. Suppose . We need to show that $\varGamma ; \emptyset ; \varDelta _2 \vdash Q \triangleright \diamond$ can match $\ell$. The proof proceeds by a case analysis on the transition label $\ell = (\nu \, \widetilde{m_1}) n !\langle V_1 \rangle$, which is the only non-trivial case.
  From the definition of $\mathfrak {R}$ we have that if:
  25
  then there exist $\varDelta _2''$, *Q*, and $V_2$ such that:
  26
  and for a fresh *t* and some $\varDelta '_1$ and $\varDelta '_2$:
  27 $$\begin{aligned} \varGamma ; \varDelta _1' \vdash (\nu \, \widetilde{m_1})(P' \;|\;t \hookleftarrow _{\texttt {H}} V_1) \approx ^\mathtt{H} \varDelta _2' \vdash (\nu \, \widetilde{m_2})(Q' \;|\;t \hookleftarrow _{\texttt {H}} V_2 ) \end{aligned}$$
  To show that $\mathfrak {R}$ is a characteristic bisimulation after the fact that transition (25) implies transition (26), we need to show that for a fresh *t* and for some $\varDelta _3, \varDelta _4$:
  28 $$\begin{aligned} \varGamma ; \varDelta _3 \vdash (\nu \, \widetilde{m_1})(P' \;|\;t \Leftarrow _{\texttt {C}} V_1{\,:\,}U) \ \mathfrak {R}\ \varDelta _4 \vdash (\nu \, \widetilde{m_2})(Q' \;|\;t \Leftarrow _{\texttt {C}} V_2{\,:\,}U) \end{aligned}$$
  which follows from (27), Lemma 13(1), and the definition of $\mathfrak {R}$.
2. Direction $\approx ^\mathtt{C}\ \subseteq \ \approx ^\mathtt{H}$. Consider the typed relation (for readability, we omit type information):
  $$\begin{aligned} \mathfrak {R}= \{ (P, Q) \ \ |\ \ \varGamma ; \varDelta _1 \vdash P \approx ^\mathtt{C} \varDelta _2 \vdash Q\} \end{aligned}$$
  We show that $\mathfrak {R}$ is a higher-order bisimulation. Suppose  with $\ell = (\nu \, \widetilde{m_1}) n !\langle V_1 \rangle$. We need to show that $\varGamma ; \emptyset ; \varDelta _2 \vdash Q \triangleright \diamond$ can match $\ell$.
  From the definition of $\mathfrak {R}$ we have that if:
  29
  then there exist $\varDelta ''_2$, *Q*, and $V_2$ such that:
  30
  and for a fresh *t* and some $\varDelta '_1, \varDelta '_2$:
  31 $$\begin{aligned} \varGamma ; \varDelta _1' \vdash (\nu \, \widetilde{m_1})(P' \;|\;t \Leftarrow _{\texttt {C}} V_1{\,:\,}U) \approx ^\mathtt{C} \varDelta _2' \vdash (\nu \, \widetilde{m_2})(Q' \;|\;t \Leftarrow _{\texttt {C}} V_2{\,:\,}U ) \end{aligned}$$
  To show that $\mathfrak {R}$ is a higher-order bisimulation after the fact that transition (29) implies transition (30), we need to show that for a fresh *t* and some $\varDelta _3, \varDelta _4$:
  32 $$\begin{aligned} \varGamma ; \varDelta _3 \vdash (\nu \, \widetilde{m_1})(P' \;|\;t \hookleftarrow _{\texttt {H}} V_1) \ \mathfrak {R}\ \varDelta _4 \vdash (\nu \, \widetilde{m_2})(Q' \;|\;t \hookleftarrow _{\texttt {H}} V_2) \end{aligned}$$
  which follows from (31), Lemma 13(2), and the definition of $\mathfrak {R}$.$\square$

We state an auxiliary lemma that captures a property of trigger processes in terms of process equivalence.

#### Lemma 15

(Trigger process application) Let *P* and *Q* be processes. Also, let *t* be a fresh name.

1. If $n_1 \not = n_2$ with $\varGamma ; \emptyset ; \varDelta \vdash n_i \triangleright U$ with $U \not = \texttt {end}$ and
  $$\begin{aligned} \varGamma ; \varDelta _1 \vdash (\nu \, \widetilde{m_1})(P \;|\;(\lambda x.\,t ?(y) . (y\, {x}))\, {n_1} ) \approx ^\mathtt{H} \varDelta _2 \vdash (\nu \, \widetilde{m_2})(Q \;|\;(\lambda x.\,t ?(y) . (y\, {x}))\, {n_2} ) \end{aligned}$$
  then $n_1, n_2$ are session names and $\overline{n_1} \in \texttt {fn}(P)$ and $\overline{n_2} \in \texttt {fn}(Q)$.
2. If $\varGamma ; \varDelta _1 \vdash (\nu \, \widetilde{m_1})(P \;|\;[\!\!(U)\!\!]_{\textsf {c}}\, {n_1} ) \approx ^\mathtt{H} \varDelta _2 \vdash (\nu \, \widetilde{m_2})(Q \;|\;[\!\!(U)\!\!]_{\textsf {c}}\, {n_2} )$ then for all $\ell$ whenever
  then there exist $\varDelta _2'$, $(\nu \, \widetilde{m_2}')(Q' \;|\;(\lambda x.\,t ?(y) . (y\, {x}))\, {n_2} )$ such that
  with $$\ell _2 = \breve{\ell }$$.
3. If $$ \varGamma ; \varDelta _1 \vdash (\nu \, \widetilde{m_1})(P \;|\;t !\langle n_1 \rangle . \mathbf {0}) \approx ^\mathtt{H} \varDelta _2 \vdash (\nu \, \widetilde{m_2})(Q \;|\;t !\langle n_2 \rangle . \mathbf {0}) $$ then
  $$\begin{aligned} \varGamma ; \varDelta _1 \vdash (\nu \, m_1)(P \;|\;t ?(x) .{(x\, {n_1})}) \approx ^\mathtt{H} \varDelta _2 \vdash (\nu \, m_2)(Q \;|\;t ?(x) .{(x\, {n_2})}) \end{aligned}$$
4. If *n* is fresh and
  $$\begin{aligned} \varGamma ; \varDelta _1 \vdash (\nu \, \widetilde{m_1})(P n/x \;|\;t !\langle n_1 \rangle . \mathbf {0}) \approx ^\mathtt{H} \varDelta _2 \vdash (\nu \, \widetilde{m_2})(Q n/x \;|\;t !\langle m_1 \rangle . \mathbf {0}) \end{aligned}$$
  then
  $$\begin{aligned} \varGamma ; \varDelta _1 \vdash (\nu \, \widetilde{m_1})(P n_1/x ) \approx ^\mathtt{H} \varDelta _2 \vdash (\nu \, \widetilde{m_2})(Q m_1/x ) \end{aligned}$$

#### Proof

We analyse each part separately:

1. The proof for Part 1 is by contradiction. Assume that $\overline{n_1} \notin \texttt {fn}(P)$ or $\overline{n_2} \notin \texttt {fn}(Q)$. Then the bisimulation requirement allows us to observe the following transition, for some $U \not = \texttt {end}$. Note that the shape of $[\!\!(U)\!\!]^{n_1}$ enables an observable action on $n_1$, which results in the process $t' !\langle n_1 \rangle . \mathbf {0}$:
  where $C = \mathbf {0}$ if $\ell$ is not an input action, and $C = [\!\!(U')\!\!]^{m}$ if $\ell$ is an input action and $\texttt {subj}(\ell ) = n_1$. Because of the characteristic process interaction, from the freshness of *t*, we have:
  with $\texttt {subj}(\breve{\ell }) = n_2$ But since $(\nu \, \widetilde{m_1})(P \;|\;t' !\langle n_1 \rangle . \mathbf {0})$ has an action on $t'$ not present in $(\nu \, \widetilde{m_2})(Q' \;|\;C \;|\;[\!\!(U\!\! \rightarrow \! \diamond )\!\!]^{\,} {n_2})$, we derive a contradiction with respect to the bisimilarity assumption.
2. The proof for Part 2 is also by contradiction. Assume that
  From the bisimilarity requirement we can observe
  But then we can observe an action on the fresh name *t* on process
  $$\begin{aligned} \varGamma ; \emptyset ; \varDelta _1' \vdash (\nu \, \widetilde{m_1}')(P' \;|\;(\lambda x.\,t ?(y) . (y\, {x}))\, {n_1} ) \triangleright \diamond \end{aligned}$$
  that cannot be observed by process $\varGamma ; \emptyset ; \varDelta _2' \vdash (\nu \, \widetilde{m_2}')(Q' \;|\;[\!\!(U)\!\!]_{\textsf {c}}\, {n_2} )$—a contradiction.
3. For the proof of Part 3 we do a case analysis on the transitions for checking the bisimulation requirements. The most interesting case is when, for some $\varDelta ''_1$:
  From the freshness of *t* we can derive that, for some $\varDelta ''_2$ and $Q''$
  From the bisimulation requirement of the hypothesis we have that
  implies
  for some $\varDelta '_1, \varDelta '_2$ and
  $$\begin{aligned} \begin{array}{rcll} \varGamma ; \emptyset ; &{}\varDelta _1'&{} \vdash &{} (\nu \, \widetilde{m_1}')(P \;|\;t ?(x) . (\nu \, s)( s ?(y) .(x\, {y}) \;|\;\overline{s} !\langle n_1 \rangle . \mathbf {0}) ) \\ \approx ^\mathtt{H} &{}\varDelta _2'&{} \vdash &{} (\nu \, \widetilde{m_2}')(Q' \;|\;t ?(x) . (\nu \, s)( s ?(y) .(x\, {y}) \;|\;\overline{s} !\langle n_2 \rangle . \mathbf {0})) \end{array} \end{aligned}$$
  Whenever
  then for some $Q_2''$
  which concludes the case.
4. For the proof of Part 4, let $\mathfrak {R}$ be the typed relation
  $$\begin{aligned} \mathfrak {R}= & {} \{ \varGamma ; \varDelta _1 \vdash (\nu \, \widetilde{m_1})(P n_1/x ) \approx ^\mathtt{H} \varDelta _2 \vdash (\nu \, \widetilde{m_2})(Q m_1/x ) \ \ |\ \ \\&\varGamma ; \varDelta _1 \vdash (\nu \, \widetilde{m_1})(P n/x \;|\;t_1 !\langle n_1 \rangle . \mathbf {0}) \approx ^\mathtt{H} \varDelta _2 \vdash (\nu \, \widetilde{m_2})(Q n/x \;|\;t_1 !\langle m_1 \rangle . \mathbf {0}) \} \end{aligned}$$
  Suppose that $(\nu \, \widetilde{m_1})(P n_1/x)$ moves:
  We need to show a matching action from $(\nu \, \widetilde{m_2})(Q m_1/x)$; we proceed to show that $\mathfrak {R}$ is a higher-order bisimulation by a case analysis on the subject/shape of action $\ell$. There are three cases:
  - If $\texttt {subj}(\ell ) \not = n_1$ then the proof is straightforward from the premise of the proposition. First observe that
    implies
    for some $\varDelta '_2$ and
    $$\begin{aligned} \varGamma ; \varDelta _2 \vdash (\nu \, \widetilde{m_1}')(P' n/x) \;|\;t !\langle n_1 \rangle . \mathbf {0}\;|\;C_1 \approx ^\mathtt{H} \varDelta _2' \vdash (\nu \, \widetilde{m_2}')(Q' n/x) \;|\;t !\langle m_1 \rangle . \mathbf {0}\;|\;C_2 \end{aligned}$$
    with $C_1 = t \hookleftarrow _{\texttt {H}} n_1$ and $C_2 = t \hookleftarrow _{\texttt {H}} m_1$ if $\ell$ and $\breve{\ell }$ are output actions, $C_1 = \mathbf {0}$ and $C_2 = \mathbf {0}$ otherwise. From here we can deduce that
    Furthermore, we can easily see that
    $$\begin{aligned} \varGamma ; \varDelta _2 \vdash (\nu \, \widetilde{m_1}')(P n_1/x) \;|\;C_1 \ \mathfrak {R}\ \varDelta _2' \vdash (\nu \, \widetilde{m_2}')(Q' m_1/x) \;|\;C_2 \end{aligned}$$
  - $\texttt {subj}(\ell ) = n_1$. We distinguish two sub-cases:
    - $n_1 = m_1$. The case is similar to the previous case.
    - $n_1 \not = m_1$. From the premise and Part 1 of this lemma we get that $\overline{n_1} \in \texttt {fn}(P)$ and $\overline{m_1} \in \texttt {fn}(Q)$. The latter implies that this case is not possible, since no external action $\ell$ would be observed, because of the typed transition requirement.
  - $\ell = \tau$. This implies the untyped transitions:
    33
    34
    35
    We distinguish two sub-cases:
    - $\texttt {subj}(\ell _{11}') \not = n_1$. This case is similar to Case 1 of this proof.
    - $\texttt {subj}(\ell _{11}') = n_1$. First observe that
      for some $\varDelta '_1$ with $\ell _{11}'' n_1/n = \ell _{11}'$, which implies
      with $\ell _{21}'' m_1/n = \ell _{21}'$, which in turn implies
      36
      Also observe that
      for some $\varDelta ''''_1$ which implies
      for some $\varDelta ''''_2$ with
      $$\begin{aligned} \begin{array}{rcll} \varGamma ; \emptyset ; &{}\varDelta _1''''&{} \vdash &{} (\nu \, \widetilde{m_1}'')(P n/x \;|\;t' \hookleftarrow _{\texttt {H}} n_1)\\ \approx ^\mathtt{H} &{}\varDelta _2''''&{} \vdash &{} (\nu \, \widetilde{m_2}'')(Q' n/x \;|\;t' \hookleftarrow _{\texttt {H}} m_1) \end{array} \end{aligned}$$
      From here observe that for $U = \varDelta '''(n_1)$
      for some $\varDelta ''_1$, which implies
      for some $\varDelta ''_2$ with
      $$\begin{aligned} \begin{array}{rcll} \varGamma ; \emptyset ; &{}\varDelta _1''&{} \vdash &{} (\nu \, \widetilde{m_1}'')(P n/x \;|\;[\!\!(U)\!\!]^{x} n_1/x)\\ \approx ^\mathtt{H} &{}\varDelta _2''&{} \vdash &{} (\nu \, \widetilde{m_2}'')(Q' n/x \;|\;[\!\!(U)\!\!]^{x} m_1/x) \end{array} \end{aligned}$$
      From (34), i.e., the fact that the two parallel components of the process interact on name $n_1$, we can see that, for some $\varDelta '''_1$
      where $C_1 = \mathbf {0}$ if the action on $[\!\!(U)\!\!]^{x} n_1/x$ is not an input action and $C_1 = [\!\!(U')\!\!]^{a}$ otherwise. This in turn implies from Part 2 of this lemma
      37
      for some $\varDelta '''_2$ and $C_2 = \mathbf {0}$ when the action on $[\!\!(U)\!\!]^{x} m_1/x$ is not an input action and $C_2 = [\!\!(U')\!\!]^{b}$ otherwise. It is then implied that
      38 $$\begin{aligned} \begin{array}{rcll} \varGamma ; \emptyset ; &{}\varDelta _1'''&{} \vdash &{} (\nu \, \widetilde{m_1}'')(P_2 n/x \;|\;C_1 \;|\;t' !\langle n_1 \rangle . \mathbf {0})\\ \approx ^\mathtt{H} &{}\varDelta _2'''&{} \vdash &{} (\nu \, \widetilde{m_2}'')(Q_2 n/x \;|\;C_2 \;|\;t' !\langle m_1 \rangle . \mathbf {0}) \end{array} \end{aligned}$$
      where (37) implies the untyped transition
      and furthermore,
      with $\ell _{22}'' m_1/n = \ell _{22}'$. From the last result and (36) we get
      Furthermore, from (38) we can get that, for some $\varDelta _3$,
      which implies
      and
      which in turn implies the required conclusion:
      $$\begin{aligned} \varGamma ; \varDelta _1' \vdash (\nu \, \widetilde{m_1}''')(P' n_1/x \;|\;C_1) \ \mathfrak {R}\ \varDelta _2' \vdash (\nu \, \widetilde{m_2}''')(Q'' m_1/x \;|\;C_2) \end{aligned}$$

$$\square $$

A process substitution lemma is useful for showing the contextuality property for higher-order and characteristic bisimilarities. Before we state and prove a process substitution lemma, we give an intermediate result. (This is Lemma 4 in the main text.)

#### Lemma 16

(Trigger substitution) Let *P* and *Q* be processes. Suppose that all $t_i, i \in I$ are fresh names. If

$$\begin{aligned} \varGamma ; \varDelta _1 \vdash (\nu \, \widetilde{m_1})\left( P \;|\;\prod _{i \in I} (\lambda x.\,t_i ?(y) . (y\, {x}))\, {n_i} \right) \\ \approx ^\mathtt{H} \varDelta _2 \vdash (\nu \, \widetilde{m_2})\left( Q \;|\;\prod _{i \in I} (\lambda x.\,t_i ?(y) . (y\, {x}))\, {m_i} \right) \end{aligned}$$

then for all $\lambda \widetilde{x}.\,R$ there exist $\varDelta _1', \varDelta _2'$ such that

$$\begin{aligned} \varGamma ; \varDelta _1' \vdash (\nu \, \widetilde{m_1})(P \;|\;(\lambda \widetilde{x}.\,R)\, {\widetilde{n}} ) \approx ^\mathtt{H} \varDelta _2' \vdash (\nu \, \widetilde{m_2})(Q \;|\;(\lambda \widetilde{x}.\,R)\, {\widetilde{m}} ). \end{aligned}$$

#### Proof

We prove the result up-to the application of names $n_i$ and $m_i$ to process *R*. Let $\mathfrak {R}$ be the relation

$$\begin{aligned} \mathfrak {R}= & {} \left\{ (\varGamma ; \varDelta _1' \vdash (\nu \, \widetilde{m_1})(P \;|\;R \widetilde{n}/\widetilde{x} )\right. \\&\varDelta _2' \vdash (\nu \, \widetilde{m_2})(Q \;|\;R \widetilde{m}/\widetilde{x} )) \ \ |\ \ \forall \lambda \widetilde{x}.\,R, \exists \varDelta _1', \varDelta _2'.\\&\varGamma ; \varDelta _1 \vdash (\nu \, \widetilde{m_1})\left( P \;|\;\prod _{i \in I} (\lambda x.\,t_i ?(y) . (y\, {x}))\, {n_i} \right) \\&\left. \approx ^\mathtt{H} \vdash \varDelta _2{(\nu \, \widetilde{m_2})\left( Q \;|\;\prod _{i \in I} (\lambda x.\,t_i ?(y) . (y\, {x}))\, {m_i}\right) }\right\} \end{aligned}$$

We show that $\mathfrak {R}$ is a higher-order bisimulation. The proof is done by a case analysis on the actions that can be observed on the pairs of processes, so to check their higher-order bisimulation requirements. There are three cases:

1. Suppose an action from *P*, for some $\varDelta _1''$:
  This transition implies, for some $\varDelta _3'$, the following:
  which in turn implies, for some $\varDelta _5$:
  The latter implies the following, from the definition of $\approx ^\mathtt{H}$ and the freshness of $t_i$, for some $\varDelta _6$:
  and
  39 $$\begin{aligned}&\begin{array}{rcll} \varGamma ; \emptyset ; &{}\varDelta _5&{} \vdash &{} (\nu \, \widetilde{m_1}')\left( P' \;|\;\prod _{i \in I} (\lambda x.\,t_i ?(y) . (y\, {x}))\, {n_i} \;|\;C_1\right) \\ \approx ^\mathtt{H} &{}\varDelta _6&{} \vdash &{} (\nu \, \widetilde{m_2}')\left( Q' \;|\;\prod _{i \in I} (\lambda x.\,t_i ?(y) . (y\, {x}))\, {m_i} \;|\;C_2\right) \end{array} \end{aligned}$$
  where $C_1, C_2$ are higher-order trigger processes if $\ell , \breve{\ell }$ are output actions, and $C_1 = C_2 = \mathbf {0}$ otherwise. At this point we can infer, for some $\varDelta _4'$:
  which in turn implies, for some $\varDelta _2''$:
  Equation (39) and the definition of $\mathfrak {R}$ imply the desired conclusion for the case:
  $$\begin{aligned} \varGamma ; \varDelta _1'' \vdash (\nu \, \widetilde{m_1}')(P' \;|\;R \widetilde{n}/\widetilde{x} \;|\;C_1) \ \mathfrak {R}\ \varDelta _2'' \vdash (\nu \, \widetilde{m_2}')(Q' \;|\;R \widetilde{m}/\widetilde{x} \;|\;C_2 ) \end{aligned}$$
2. Suppose an action from *R*:
  for some $\varDelta _1''$. We identify three sub-cases:
  i. $\texttt {subj}(\ell ) \not = n_i$, i.e.  the subject of $\ell$ is not in $\widetilde{n}$. The case is similar as above.
  ii. $\texttt {subj}(\ell ) = n_k$ and $n_k = m_k$. From the definition of $\mathfrak {R}$ we get that
    for some $\varDelta _3$. Recall that $[\!\!(U\!\! \rightarrow \! \diamond )\!\!]_{\textsf {c}} = \lambda x.\,[\!\!(U)\!\!]^{x}$ (cf. Fig. 6); this transition implies
    and from bisimilarity up-to deterministic transition (Lemma 1):
    for some $\varDelta _4$. From the shape of $[\!\!(U)\!\!]^{x}$ we can observe
    implies, for some $\varDelta _4'$:
    and furthermore, from Part 3 of Lemma 15
    $$\begin{aligned}&\begin{array}{rcll} \varGamma ; \emptyset ; &{}\varDelta _3'&{} \vdash &{} (\nu \, \widetilde{m_1}')\left( P \;|\;\prod _{i \in I \backslash {\{k\}}} t_i ?(x) . (x\, {n_i}) \;|\;t' ?(y) .{(y\, {n_k})}\right) \\ \approx ^\mathtt{H} &{}\varDelta _4'&{} \vdash &{} (\nu \, \widetilde{m_2}')\left( Q'' \;|\;\prod _{i \in I \backslash {\{k\}}} t_i ?(x) . (x\, {m_i}) \;|\;t' ?(y) .{(y\, {m_k})}\right) \end{array} \end{aligned}$$
    that implies from the definition of $\mathfrak {R}$ that for all *R* such that $\{\widetilde{x}\} \subseteq \texttt {fv}(R)$
    $$\begin{aligned} \varGamma ; \varDelta _3' \vdash (\nu \, \widetilde{m_1}')(P \;|\;R \widetilde{n}/\widetilde{x} ) \ \mathfrak {R}\ \varDelta _4' \vdash (\nu \, \widetilde{m_2}')(Q'' \;|\;R \widetilde{m}/\widetilde{x}) \end{aligned}$$
    The case concludes when we verify that, for some $\varDelta _2''$, we have:
  iii. $\texttt {subj}(\ell ) = n_k$ and $n_k \not = m_k$. This case is not possible. Lemma 15 implies that $n_k$ is a session and $\overline{n_k} \in \texttt {fn}(P)$. From the definition of typed transition (Definition 5) we get that we cannot observe $\ell$ on $R \widetilde{n}/\widetilde{x}$, because .
3. Suppose the interaction of *P* and *R*, for some $\varDelta _1'$:
  From the typed reduction definition (Definition 5) we get that
  40
  41
  We distinguish several subcases:
  i. $\ell _1 = n_k ?\langle V \rangle$ and $\ell _2 = (\nu \, \widetilde{m})(\overline{n_k} !\langle V \rangle )$. From the requirement of $\mathfrak {R}$ we get that there exists $U\!\! \rightarrow \! \diamond$ such that, for some $\varDelta _3$:
    which in turn implies, for some $\varDelta _4$, that
    and
    $$\begin{aligned} \begin{array}{rcll} \varGamma ; \emptyset ; &{}\varDelta _3&{} \vdash &{} (\nu \, \widetilde{m_1}')\left( P \;|\;\prod _{i \in I\backslash {\{k\}}} t_i ?(x) . (x\, {n_i}) \;|\;[\!\!(U)\!\!]^{x} n_k/x \right) \\ \approx ^\mathtt{H} &{}\varDelta _4&{} \vdash &{} (\nu \, \widetilde{m_2}')\left( Q' \;|\;\prod _{i \in I\backslash {\{k\}}} t_i ?(x) . (x\, {m_i}) \;|\;[\!\!(U)\!\!]^{x} m_k/x \right) \end{array} \end{aligned}$$
    From the shape of $[\!\!(U)\!\!]^{x}$ we can observe the interaction between $[\!\!(U)\!\!]^{x}$ and *P* to obtain that if, for some $\varDelta _3'$ and some $[\!\!(U')\!\!]_{\textsf {c}}$ (cf. Definition 13), we have
    then
    and
    42 $$\begin{aligned} \begin{array}{rcll} \varGamma ; \emptyset ; &{}\varDelta _3'&{} \vdash &{} (\nu \, \widetilde{m_1}'')\left( P' [\!\!(U')\!\!]_{\textsf {c}}/x \;|\;\prod _{i \in I\backslash {\{k\}}} t_i ?(x) . (x\, {n_i}) \;|\;t'_k !\langle n_k \rangle . \mathbf {0}\right) \\ \approx ^\mathtt{H} &{}\varDelta _4'&{} \vdash &{} (\nu \, \widetilde{m_2}'')\left( Q'' [\!\!(U')\!\!]_{\textsf {c}}/x \;|\;\prod _{i \in I\backslash {\{k\}}} t_i ?(x) . (x\, {m_i}) \;|\;t'_k !\langle m_k \rangle . \mathbf {0}\right) \end{array} \end{aligned}$$
    for some $\varDelta _4'$. From Lemma 15(3) we obtain
    43 $$\begin{aligned} \begin{array}{rcll} \varGamma ; \emptyset ; &{}\varDelta _3''&{} \vdash &{} (\nu \, \widetilde{m_1}'')\left( P' [\!\!(U')\!\!]_{\textsf {c}}/x \;|\;\prod _{i \in I\backslash {\{k\}}} t_i ?(x) . (x\, {n_i}) \;|\;t'_k ?(x) . (x\, {n_k})\right) \\ \approx ^\mathtt{H} &{}\varDelta _4''&{} \vdash &{} (\nu \, \widetilde{m_2}'')\left( Q'' [\!\!(U')\!\!]_{\textsf {c}}/x \;|\;\prod _{i \in I\backslash {\{k\}}} t_i ?(x) . (x\, {m_i}) \;|\;t'_k ?(x) . (x\, {m_k})\right) \end{array} \end{aligned}$$
    From the definition of $\mathfrak {R}$ we get that
    $$\begin{aligned} \begin{array}{rcll} \varGamma ; \emptyset ; &{}\varDelta _3''&{} \vdash &{} (\nu \, \widetilde{m_1}'')(P' [\!\!(U')\!\!]_{\textsf {c}}/x \;|\;R' \widetilde{n}/\widetilde{x})\\ \mathfrak {R} &{}\varDelta _4''&{} \vdash &{} (\nu \, \widetilde{m_2}'')(Q'' [\!\!(U')\!\!]_{\textsf {c}}/x \;|\;R' \widetilde{m}/\widetilde{x}) \end{array} \end{aligned}$$
    From the above result we can match actions in (40) and (41):
    to obtain, for some $\varDelta _2''$, that
    Furthermore the definition of $\mathfrak {R}$ and (42) allow us to conclude the case:
    $$\begin{aligned} \varGamma ; \varDelta _1'' \vdash (\nu \, \widetilde{m_1}')(P'V/x \;|\;R' \widetilde{n}/\widetilde{x} ) \ \mathfrak {R}\ \varDelta _2'' \vdash (\nu \, \widetilde{m_2}')(Q'' V/x \;|\;R' \widetilde{m}/\widetilde{x} ) \end{aligned}$$
  ii. An important sub-case is when $\ell _1 = n ?\langle n_k \rangle$ and $\ell _2 = n !\langle n_k \rangle$. From the definition of $\mathfrak {R}$ we have that
    for some $\varDelta _3$. This transition implies, for some $\varDelta _4$, that
    44
    and
    $$\begin{aligned}&\varGamma ; \varDelta _3 \vdash (\nu \, \widetilde{m_1})\left( P' m/x \;|\;\prod _{i \in I} t_i ?(x) . (x\, {n_i})\right) \\&\quad \approx ^\mathtt{H} \varDelta _4 \vdash (\nu \, \widetilde{m_2})\left( Q' m/x \;|\;\prod _{i \in I} t_i ?(x) . (x\, {m_i})\right) \end{aligned}$$
    We infer from Lemma 15(4) that
    $$\begin{aligned} \begin{array}{rcll} \varGamma ; \emptyset ; &{}\varDelta _3'&{} \vdash &{} (\nu \, \widetilde{m_1})\left( P' n_k/x \;|\;\prod _{i \in I\backslash \{k\}} t_i ?(x) .(x\, {n_i})\right) \\ \approx ^\mathtt{H} &{}\varDelta _4'&{} \vdash &{} (\nu \, \widetilde{m_2})\left( Q' m_k/x \;|\;\prod _{i \in I\backslash \{k\}} t_i ?(x) .(x\, {m_i})\right) \end{array} \end{aligned}$$
    which implies from the definition of $\mathfrak {R}$ that
    $$\begin{aligned} \begin{array}{rcll} \varGamma ; \emptyset ; &{}\varDelta _1'&{} \vdash &{} (\nu \, \widetilde{m_1})(P' n_k/x \;|\;R \widetilde{n}/\widetilde{x})\\ \ \mathfrak {R}\ &{}\varDelta _2'&{} \vdash &{} (\nu \, \widetilde{m_2})(Q' m_k/x \;|\;R' \widetilde{m}/\widetilde{x} ) \end{array} \end{aligned}$$
    From (44) and (41) we obtain, for some $\varDelta _2''$, the following
    which concludes the case.
  iii. The sub-case $\ell _1 = n_k ?\langle n_l \rangle$ and $\ell _2 = n_k !\langle n_l \rangle$. The proof is a consequence of the previous two sub-cases.
  iv. The rest of the sub-cases are similar (or easier) to the above cases.$\square$

We can now state a process substitution lemma (Lemma 3 in the main text). Given a higher-order bisimulation under a trigger value substitution, we can generalise for any value substitution.

#### Lemma 17

(Process substitution) Let $P_1$ and $P_2$ be processes, with $z \in \texttt {fv}(P_1)$ and $z \in \texttt {fv}(P_2)$. Also, let *t* be a fresh name. If

$$\begin{aligned} \varGamma ; \varDelta _1 \vdash (\nu \, \widetilde{m_1})(P_1 \lambda x.\,t ?(y) . (y\, {x})/z ) \approx ^\mathtt{H} \varDelta _2 \vdash (\nu \, \widetilde{m_2})(P_2 \lambda x.\,t ?(y) . (y\, {x})/z ) \end{aligned}$$

then for all $\lambda x.\,R$ there exist $\varDelta _1'$ and $\varDelta _2'$ such that

$$\begin{aligned} \varGamma ; \varDelta _1' \vdash (\nu \, \widetilde{m_1})(P_1 {\lambda x.\,R}/z ) \approx ^\mathtt{H} \varDelta _2' \vdash (\nu \, \widetilde{m_2})(P_2 {\lambda x.\,R}/z ) \end{aligned}$$

#### Proof

Consider the typed relation (for readability, we omit type information):

$$\begin{aligned} \mathfrak {R}= & {} \{ (\varGamma ; \varDelta _1' \vdash (\nu \, \widetilde{m_1})(P_1 {\lambda x.\,R}/z), \varDelta _2' \vdash (\nu \, \widetilde{m_2})(P_2 {\lambda x.\,R}/z)) \ \ |\ \ \\&\qquad \varGamma ; \varDelta _1 \vdash (\nu \, \widetilde{m_1})(P_1 \lambda x.\,t ?(y) . (y\, {x})/z ) \approx ^\mathtt{H} \varDelta _2 \vdash (\nu \, \widetilde{m_2})(P_2 \lambda x.\,t ?(y) . (y\, {x})/z )\} \end{aligned}$$

We show that $\mathfrak {R}$ is a higher-order bisimulation. Suppose that

45

for some $\varDelta _3$. We should exhibit an appropriate matching action from $(\nu \, \widetilde{m_2})(P_2 {\lambda x.\,R}/z)$. Our analysis distinguishes two cases, depending on whether the substitution ${\lambda x.\,R}/z$ has an effect on the action denoted by $\ell$:

1. Case $P_1 \not \equiv Q \;|\;z\, {n}$: That is, the substitution does not affect top-level processes. In other words, we can infer from the freshness of *t* that $\texttt {subj}(\ell ) \not = t$. Furthermore, from the requirements of $\mathfrak {R}$ we get that there exist $\varDelta _1''$ and $P'_1$ such that
  which, in turn, implies that there exist $\varDelta _2''$ and $P_2'$ such that
  46
  and
  $$\begin{aligned} \varGamma ; \varDelta _1 \vdash (\nu \, \widetilde{m_1}'')(P_1' \lambda x.\,t ?(y) . (y\, {x})/z \;|\;C_1) \approx ^\mathtt{H} \varDelta _2 \vdash (\nu \, \widetilde{m_2}'')(P_2' \lambda x.\,t ?(y) . (y\, {x})/z \;|\;C_2) \end{aligned}$$
  with $C_1$ (resp., $C_2$) being the higher-order trigger process in the cases where $\ell = (\nu \, \widetilde{m}) n !\langle V_1 \rangle$ (resp., $\breve{\ell } = (\nu \, \widetilde{m}') n !\langle V_2 \rangle$), and $C_1 = C_2 = \mathbf {0}$ otherwise. Because $C_1$ and $C_2$ are closed terms we can rewrite the substitution as:
  $$\begin{aligned} \varGamma ; \varDelta _1 \vdash (\nu \, \widetilde{m_1}'')((P_1'\;|\;C_1) \lambda x.\,t ?(y) . (y\, {x})/z) \approx ^\mathtt{H} \varDelta _2 \vdash (\nu \, \widetilde{m_2}'')((P_2'\;|\;C_2) \lambda x.\,t ?(y) . (y\, {x})/z) \end{aligned}$$
  Since $\ell , \breve{\ell }$ do not act on the substitution, we can consider the same transition with any $\lambda x.\,R$ instead of $\lambda x.\,t ?(y) . (y\, {x})$. Thus, from the definition of $\mathfrak {R}$, we further deduce that
  47 $$\begin{aligned} \varGamma ; \varDelta _3' \vdash (\nu \, \widetilde{m_1}'')((P_1'\;|\;C_1) {\lambda x.\,R}/z) \ \mathfrak {R}\ \varDelta _4' \vdash (\nu \, \widetilde{m_2}'')((P_2'\;|\;C_2) {\lambda x.\,R}/z) \end{aligned}$$
  Note that $C_1$ and $C_2$ are used to meet the bisimulation requirements for the output case. From (46) we can derive the transition
  Equation (47) concludes the case.
2. Case $P_1 \equiv P \;|\;\prod _{i \in I} z\, {n_i} \;|\;z\, {n_1}$, such that $P \not \equiv P' \;|\;z\, {n'}$. This is the case where action $\ell$ might happen on the process that is being substituted (note that a substituted process needs to be applied first).
  We identify two sub-cases, depending on the source of the action $\ell$:
  - Consider the following transition, for some $\varDelta _3$:
    This sub-case is similar to the previous case.
  - Consider the following transition, for some $\varDelta _3$, and assuming that $Q = P \;|\;\prod _{i \in I} z\, {n_i}$:
    48
    which is the application of name $n_1$ on abstraction $\lambda x.\,R$. From the requirements of $\mathfrak {R}$ we infer that
    for some $\varDelta _1''$. This implies that there exist $P_2'$ and $\varDelta _2''$ such that
    49
    and
    $$\begin{aligned} \begin{array}{rcll} \varGamma ; \emptyset ; &{}\varDelta _1''&{} \vdash &{} (\nu \, \widetilde{m_1})(Q \lambda x.\,t ?(y) . (y\, {x})/z \;|\;t ?(y) . (y\, {n_1}))\\ \approx ^\mathtt{H} &{}\varDelta _2''&{} \vdash &{} (\nu \, \widetilde{m_2})(P_2' \lambda x.\,t ?(y) . (y\, {x})/z) \end{array} \end{aligned}$$
    From the last pair we can see that for a fresh $t'$ if
    then from the freshness of *t*, there exist $P_2'', \varDelta _2'''$ such that
    50
    and
    $$\begin{aligned} \begin{array}{rcll} \varGamma ; \emptyset ; &{}\varDelta _1'''&{} \vdash &{} (\nu \, \widetilde{m_1})(Q \lambda x.\,t ?(y) . (y\, {x})/z \;|\;(\lambda x.\,t' ?(y) . (y\, {x}))\, {n_1})\\ \approx ^\mathtt{H} &{}\varDelta _2'''&{} \vdash &{} (\nu \, \widetilde{m_2})(P_2'' \lambda x.\,t ?(y) . (y\, {x})/z \;|\;(\lambda x.\,t' ?(y) . (y\, {x}))\, {n_2}) \end{array} \end{aligned}$$
    From Lemma 16 we can deduce that, for all $\lambda x.\,R$, there exist $\varDelta _5$ and $\varDelta _6$ such that
    $$\begin{aligned} \begin{array}{rcll} \varGamma ; \emptyset ; &{}\varDelta _5&{} \vdash &{} (\nu \, \widetilde{m_1})(Q \lambda x.\,t ?(y) . (y\, {x})/z \;|\;(\lambda x.\,R)\, {n_1})\\ \approx ^\mathtt{H} &{}\varDelta _6&{} \vdash &{} (\nu \, \widetilde{m_2})(P_2'' \lambda x.\,t ?(y) . (y\, {x})/z \;|\;(\lambda x.\,R)\, {n_2}) \end{array} \end{aligned}$$
    from the definition of $\mathfrak {R}$ we have that for all $\lambda x.\,R$, if there exist $\varDelta _3$ and $\varDelta _4$
    51 $$\begin{aligned} {\varGamma }{\varDelta _3}{(\nu \, \widetilde{m_1})(Q (\lambda x.\,R)/z \;|\;(\lambda x.\,R)\, {n_1})} {\ \mathfrak {R}\ } {\varDelta _4}{(\nu \, \widetilde{m_2})(P_2'' (\lambda x.\,R)/z \;|\;(\lambda x.\,R)\, {n_2})}\nonumber \\ \end{aligned}$$
    We show that we can mimic first the transition in (49) and then the silent part of transitions (50) to get:
    52
    We showed that if (48) then (52) and (51) as required to show that $\mathfrak {R}$ is a higher-order bisimulation.$\square$

#### Lemma 18

$$\approx ^\mathtt{H}\ \subseteq \ \approx $$.

#### Proof

Let $\mathfrak {R}$ be the typed relation (for readability, we omit typing information):

$$\begin{aligned} \mathfrak {R}= \{(P_1, Q_1) \ \ |\ \ \varGamma ; \varDelta _1 \vdash P_1 \approx ^\mathtt{H} \varDelta _2 \vdash Q_1\} \end{aligned}$$

We show that $\mathfrak {R}$ is a context bisimulation (cf. Definition 12). Suppose that

53

We need to infer an appropriate matching transition from $Q_1$. The proof proceeds by a case analysis on $\ell$. We distinguish four cases: $\ell$ is not an output or a higher-order input action; $\ell$ is a higher-order input action; $\ell$ is an higher-order output; $\ell$ is a first-order output.

1. Case $\ell \notin \{ (\nu \, \widetilde{m_1}) n !\langle \lambda \widetilde{x}.\,P \rangle , (\nu \, \widetilde{m_1}') n !\langle \widetilde{m_1} \rangle , n ?\langle \lambda \widetilde{x}.\,P \rangle \}$: We first notice that in this case the definition of $\approx$ and $\approx ^\mathtt{H}$ coincide, so we have to show the existence of $Q_2$ and $\varDelta '_2$ such that:
  and
  This is immediate from transition (53) and the definition of $\approx ^\mathtt{H}$ (cf. Definition 17).
2. Case $\ell = n ?\langle \lambda \widetilde{x}.\,P \rangle$: In this case, the transition (53) can be written as
  for some $\varDelta ''_1$. In turn, the above transition and $\mathfrak {R}$ imply the existence of $Q_2$ and $\varDelta ''_2$ such that:
  and
  $$\begin{aligned} \begin{array}{l} \varGamma ; \varDelta _1'' \vdash P_2 \lambda \widetilde{z}.\,t ?(y) . (y\, {\widetilde{z}})/x \approx ^\mathtt{H} \varDelta _2'' \vdash Q_2 \lambda \widetilde{z}.\,t ?(y) . (y\, {\widetilde{z}})/x. \end{array} \end{aligned}$$
  Then, by using the previous equality and Lemma 17, we may conclude that
  for some $\varDelta '_1$, $\varDelta '_2$, for all *P* with $\texttt {fv}(P) = \{\widetilde{x}\}$, as required.
3. Case $\ell = (\nu \, \widetilde{m_1}') n !\langle \widetilde{m_1} \rangle$: In this case, transition (53) and $\mathfrak {R}$ imply the existence of $\varDelta '_2$, a process $Q_2$, and name $m_2$ such that
  and
  for some fresh *t*. From Case 2 of this proof (higher-order input) we can conclude that for all *R* with $\texttt {fv}(R) = \{x\}$ and for some $\varDelta _1''$, $\varDelta _2''$:
  $$\begin{aligned} \begin{array}{rcl} \varGamma ; \emptyset ; \varDelta _1' \vdash (\nu \, \widetilde{m_1}')(P_2 \;|\;t \hookleftarrow _{\texttt {H}} m_1) &{} \xrightarrow {t ?\langle \lambda z.\,z ?(x) . R \rangle }&{} (\nu \, \widetilde{m_1}')(P_2 \;|\;(\nu \, s)(s ?(x) . R \;|\;\overline{s} !\langle m_1 \rangle . \mathbf {0}))\\ &{} \xrightarrow {\tau _{\textsf {d}}} &{}\varDelta _1'' \vdash (\nu \, \widetilde{m_1}')(P_2 \;|\;R m_1/x) \end{array} \end{aligned}$$
  and
  $$\begin{aligned} \begin{array}{rcl} \varGamma ; \emptyset ; \varDelta _2' \vdash (\nu \, \widetilde{m_2}')(Q_2 \;|\;t \hookleftarrow _{\texttt {H}} m_2 ) &{} \xrightarrow {t ?\langle \lambda z.\,z ?(x) . R \rangle } &{}(\nu \, \widetilde{m_2}')(Q_2 \;|\;(\nu \, s)(s ?(x) . R \;|\;\overline{s} !\langle m_2 \rangle . \mathbf {0}))\\ &{} \xrightarrow {\tau _{\textsf {d}}} &{}\varDelta _2'' \vdash (\nu \, \widetilde{m_2}')(Q_2 \;|\;R m_2/x) \end{array} \end{aligned}$$
  where, due to the deterministic internal transitions (cf. Definition 19), it is easy to see that
  $$\begin{aligned} \varGamma ; \varDelta _1'' \vdash (\nu \, \widetilde{m_1}')(P_2 \;|\;R m_1/x) \approx ^\mathtt{H} \varDelta _2'' \vdash (\nu \, \widetilde{m_2}')(Q_2 \;|\;R m_2/x) \end{aligned}$$
  for all *R* with $\texttt {fv}(R) = \{x\}$, as required by the definition of $\approx$ ((Definition 12).
4. Case $\ell = (\nu \, \widetilde{m_1}') n !\langle \lambda \widetilde{x}.\,P \rangle$: This case is similar to the previous case but makes use of the alternative trigger, $t \leftharpoonup _\texttt {A} V$ (cf. (15)). The definition of $\mathfrak {R}$ and transition (53) allow us to infer the existence of some $\varDelta _2'$, *Q*, and $Q_2$ such that
  and
  $$\begin{aligned} \varGamma ; \varDelta _1' \vdash (\nu \, \widetilde{m_1}')(P_2 \;|\;t \hookleftarrow _{\texttt {H}} \lambda \widetilde{x}.\,P) \approx ^\mathtt{H} \varDelta _2' \vdash (\nu \, \widetilde{m_2}' )(Q_2 \;|\;t \hookleftarrow _{\texttt {H}} \lambda \widetilde{x}.\,Q) \end{aligned}$$
  for some fresh *t*. Using Lemma 12, we above equality implies that
  $$\begin{aligned} \varGamma ; \varDelta _1' \vdash (\nu \, \widetilde{m_1}')(P_2 \;|\;t \leftharpoonup _\texttt {A} \lambda \widetilde{x}.\,P) \approx ^\mathtt{H} \varDelta _2' \vdash (\nu \, \widetilde{m_2}' )(Q_2 \;|\;t \leftharpoonup _\texttt {A} \lambda \widetilde{x}.\,Q) \end{aligned}$$
  which in turn implies
  for some $\varDelta _1''$ and
  for some $\varDelta _2''$, and
  $$\begin{aligned} \begin{array}{rcll} \varGamma ; \emptyset ; &{}\varDelta _1''&{} \vdash &{} (\nu \, \widetilde{m_1}')(P_2 \;|\;(\nu \, s)( (\lambda y.\,t' ?(x) . (x\, {y})) {s}\, {\;|\;}\overline{s} !\langle \lambda \widetilde{x}.\,P \rangle . \mathbf {0}) )\\ \approx ^\mathtt{H} &{}\varDelta _2''&{} \vdash &{} (\nu \, \widetilde{m_1}')(Q_2' \;|\;(\nu \, s)((\lambda y.\,t' ?(x) . (x\, {y})) {s}\, {\;|\;}\overline{s} !\langle \lambda \widetilde{x}.\,Q \rangle . \mathbf {0}) ) \end{array} . \end{aligned}$$
  From the Case 2 of this proof (higher-order input), we have
  for all *R* with $\texttt {fv}(R) = \{x\}$. Now, using deterministic transitions (cf. Definition 19) is easy to see that
  $$\begin{aligned} \varGamma ; \varDelta _1'' \vdash (\nu \, \widetilde{m_1})(P_2 \;|\;R \lambda \widetilde{x}.\,P/y) \approx ^\mathtt{H} \varDelta _2'' \vdash (\nu \, \widetilde{m_2})(Q_2 \;|\;R \lambda \widetilde{x}.\,Q/y) \end{aligned}$$
  for all *R* with $\texttt {fv}(R) = \{x\}$, as required by the definition of $\approx$ (cf. Definition 12).$\square$

#### Lemma 19

.

#### Proof

We prove that $\approx$ (cf. Definition 12) satisfies the three defining properties of $\cong$: reduction closure, barb preservation, and congruence (cf. Definition 11).

I. *Reduction-closed* Let $\varGamma ; \varDelta _1 \vdash P_1 \approx \varDelta _2 \vdash P_2$. The reduction
  $$\begin{aligned} \varGamma ; \varDelta _1 \vdash P_1 \longrightarrow \varDelta _1' \vdash P_1' \end{aligned}$$
  implies that there exist $\varDelta _2'$ and $P_2'$ such that
  $$\begin{aligned} \varGamma ; \varDelta _2 \vdash P_2 \mathop {\Longrightarrow }\limits ^{} \varDelta _2' \vdash P_2' \qquad \text {and} \qquad \varGamma ; \varDelta _1 \vdash P_1' \approx \varDelta _2' \vdash P_2' \end{aligned}$$
  The same arguments hold for the symmetric case, thus $\approx$ is reduction-closed.
II. *Barb preservation* Following Definition 9, we have that $\varGamma ; \emptyset ; \varDelta _1 \vdash P_1 \downarrow _{n}$ implies
  $$\begin{aligned} P\cong & {} (\nu \, \widetilde{m})(n !\langle V_1 \rangle . P_3 \;|\;P_4) \end{aligned}$$
  with $\overline{n} \notin \varDelta _1$. From the definition of $\approx$ we infer that
  $$\begin{aligned} \varGamma ; \varDelta _1 \vdash (\nu \, \widetilde{m})(n !\langle V_1 \rangle . P_3 \;|\;P_4) \xrightarrow {(\nu \, s_1) n !\langle V_1 \rangle } \varDelta _1' \vdash (\nu \, \widetilde{m'})(P_3 \;|\;P_4) \end{aligned}$$
  implies the existence of $\varDelta _2'$, $V_2$, and $P'_2$ such that
  $$\begin{aligned} \varGamma ; \varDelta _2 \vdash P_2 \mathop {\Longrightarrow }\limits ^{(\nu \, m_2) n !\langle V_2 \rangle } \varDelta _2' \vdash P_2' \end{aligned}$$
  Therefore, we infer that $\varGamma ; \emptyset ; \varDelta _2 \vdash P_2 \Downarrow _{n}$, as desired.
III. *Congruence* We have to show that $\approx$ is preserved under any context. The most interesting context case is parallel composition. Input congruence, which is the case that generates substitution, is straightforward, since we are dealing with closed terms.
  To show the congruence of the parallel composition we construct a typed relation defined as
  $$\begin{aligned} \begin{array}{rcl} {\mathcal {S}} &{}=&{} \{ (\varGamma ; \emptyset ; \varDelta _1 \cdot \varDelta _3 \vdash (\nu \, \widetilde{n_1})(P_1 \;|\;R) \triangleright \diamond , \varGamma ; \emptyset ; \varDelta _2 \cdot \varDelta _3 \vdash (\nu \, \widetilde{n_2})(P_2 \;|\;R)\triangleright \diamond ) \ \ |\ \ \\ &{} &{} \qquad \varGamma ; \varDelta _1 \vdash P_1 \approx \varDelta _2 \vdash P_2, \forall \varGamma ; \emptyset ; \varDelta _3 \vdash R \triangleright \diamond \}\\ \end{array} \end{aligned}$$
  We show that ${\mathcal {S}}$ is a context bisimulation. Suppose that
  $$\begin{aligned} \varGamma ; \varDelta _1 \cdot \varDelta _3 \vdash (\nu \, \widetilde{n_1})(P_1 \;|\;R) \xrightarrow {\ell } \varDelta _1' \cdot \varDelta _3 \vdash P' \end{aligned}$$
  for some $\varDelta '_1$. We must show an appropriate matching action from $(\nu \, \widetilde{n_2})(P_2 \;|\;R)$. We proceed by a case analysis on the “source” of action $\ell$ (i.e., $P_1$, *R*, an interaction between $P_1$ and *R*). There are three cases:
  1. Suppose that $\ell$ originates in $P_1$:
    $$\begin{aligned} \varGamma ; \varDelta _1 \cdot \varDelta _3 \vdash (\nu \, \widetilde{n_1})(P_1 \;|\;R) \xrightarrow {\ell } \varDelta _1' \cdot \varDelta _3 \vdash (\nu \, \widetilde{n_1'})(P_1' \;|\;R) \end{aligned}$$
    The case is divided into three sub-cases, depending on the shape of $\ell$:
    i. Sub-case $\ell \notin \{(\nu \, \widetilde{m}) n !\langle \lambda \widetilde{x}.\,Q \rangle , (\nu \, \widetilde{m}\widetilde{m_1}) n !\langle \widetilde{m_1} \rangle \}$: Then from the definition of typed transition we infer:
      $$\begin{aligned} \varGamma ; \varDelta _1 \vdash P_1 \xrightarrow {\ell } \varDelta _1' \vdash P_1' \end{aligned}$$
      which implies the existence of $P_2'$ and $\varDelta _2'$ such that
      54 $$\begin{aligned}&\varGamma ; \varDelta _1 \vdash P_2 \mathop {\Longrightarrow }\limits ^{\ell } \varDelta _2' \vdash P_2' \end{aligned}$$
      55 $$\begin{aligned}&\varGamma ; \varDelta _1' \vdash P_1' \approx \varDelta _2' \vdash P_2'. \end{aligned}$$
      From transition (54) we may infer that
      $$\begin{aligned} \varGamma ; \varDelta _2 \cdot \varDelta _3 \vdash (\nu \, \widetilde{n_2})(P_2 \;|\;R) \mathop {\Longrightarrow }\limits ^{\ell } \varDelta _2' \cdot \varDelta _3 \vdash (\nu \, \widetilde{n_2}')(P_2' \;|\;R) \end{aligned}$$
      Furthermore, from (55) and the definition of ${\mathcal {S}}$ we infer the desired conclusion:
      $$\begin{aligned} \varGamma ; \varDelta _1' \cdot \varDelta _3 \vdash (\nu \, \widetilde{n_1}')(P_1' \;|\;R) \ {\mathcal {S}}\ \varDelta _2' \cdot \varDelta _3 \vdash (\nu \, \widetilde{n_2}')(P_2' \;|\;R) \end{aligned}$$
    ii. Sub-case $\ell = (\nu \, \widetilde{m_1}) n !\langle \lambda \widetilde{x}.\,Q_1 \rangle$: Then we infer the typed transition
      $$\begin{aligned} \varGamma ; \varDelta _1 \vdash P_1 \xrightarrow {(\nu \, \widetilde{m_1}) n !\langle \lambda \widetilde{x}.\,Q_1 \rangle } \varDelta _1' \vdash P_1' \end{aligned}$$
      which implies the existence of $P'_2$, $\varDelta _2'$, $\varDelta _1''$, and $\varDelta _2''$ such that
      56 $$\begin{aligned} \varGamma ; \varDelta _1 \vdash P_2 \mathop {\Longrightarrow }\limits ^{(\nu \, \widetilde{m_2}) n !\langle \lambda \widetilde{x}.\,Q_2 \rangle } \varDelta _2' \vdash P_2' \end{aligned}$$
      and
      57 $$\begin{aligned} \varGamma ; \varDelta _1'' \vdash (\nu \, \widetilde{n_1}'')(P_1' \;|\;Q \lambda \widetilde{x}.\,Q_1/x) \ \approx \ \varDelta _2'' \vdash (\nu \, \widetilde{n_2}'')(P_2' \;|\;Q \lambda \widetilde{x}.\,Q_2/x)\nonumber \\ \end{aligned}$$
      for all *Q* with $x \in \texttt {fv}(Q)$. From transition (56), we infer that
      $$\begin{aligned} \varGamma ; \varDelta _2 \cdot \varDelta _3 \vdash (\nu \, \widetilde{n_2})(P_2 \;|\;R) \mathop {\Longrightarrow }\limits ^{(\nu \, \widetilde{m_2}) n !\langle \lambda \widetilde{x}.\,Q_2 \rangle } \varDelta _2' \cdot \varDelta _3 \vdash (\nu \, \widetilde{n_2}')(P_2' \;|\;R) \end{aligned}$$
      Furthermore, from (57) we conclude that
      $$\begin{aligned}&\varGamma ; \varDelta _1'' \cdot \varDelta _3 \vdash (\nu \, \widetilde{n_1}'')(P_1' \;|\;Q \lambda \widetilde{x}.\, Q_1/x \;|\;R) \ {\mathcal {S}}\ \\ \vdash&\quad {\varDelta _2'' \cdot \varDelta _3}{(\nu \, \widetilde{n_2}'')(P_2' \;|\;Q \lambda \widetilde{x}.\,Q_2/x \;|\;R)} \end{aligned}$$
      for all *Q*, with $x \in \texttt {fv}(Q)$, as desired.
    iii. Sub-case $\ell = (\nu \, \widetilde{m}\widetilde{m_1}) n !\langle \widetilde{m_1} \rangle$: From the definition of typed transition we infer that
      $$\begin{aligned} \varGamma ; \varDelta _1 \vdash P_1 \xrightarrow {(\nu \, \widetilde{m}\widetilde{m_1}) n !\langle \widetilde{m_1} \rangle } \varDelta _1' \vdash P_1' \end{aligned}$$
      which, in turn, implies that there exist $\varDelta _2'$, $P_2'$, and $m_2$ such that
      58 $$\begin{aligned} \varGamma ; \varDelta _1 \vdash P_2 \mathop {\Longrightarrow }\limits ^{(\nu \, \widetilde{m}\widetilde{m_2}) n !\langle \widetilde{m_2} \rangle } \varDelta _2' \vdash P_2' \end{aligned}$$
      and
      59 $$\begin{aligned} \varGamma ; \varDelta _1'' \vdash (\nu \, \widetilde{n_1})(P_1' \;|\;Q \widetilde{m_1}/\widetilde{x}) \ \approx \ \varDelta _2'' \vdash (\nu \, \widetilde{n_2})(P_2' \;|\;Q \widetilde{m_2}/\widetilde{x}) \end{aligned}$$
      for some $\varDelta _1''$ and $\varDelta _2''$, for all *Q* with $\{x\} = \texttt {fv}(Q)$. From transition (58) we infer that
      $$\begin{aligned} \varGamma ; \varDelta _2 \cdot \varDelta _3 \vdash (\nu \, \widetilde{n_2}')(P_2 \;|\;R) \mathop {\Longrightarrow }\limits ^{(\nu \, \widetilde{m}\widetilde{m_2}) n !\langle \widetilde{m_2} \rangle } \varDelta _2' \cdot \varDelta _3 \vdash (\nu \, \widetilde{n_2}''')(P_2' \;|\;R) \end{aligned}$$
      Furthermore, from (59) we conclude that
      $$\begin{aligned} \varGamma ; \varDelta _1'' \cdot \varDelta _3 \vdash (\nu \, \widetilde{n_1}'')(P_1' \;|\;Q \widetilde{m_1}/\widetilde{x} \;|\;R) \ {\mathcal {S}}\ \varDelta _2'' \cdot \varDelta _3 \vdash (\nu \, \widetilde{n_2}'')(P_2' \;|\;Q \widetilde{m_2}/\widetilde{x} \;|\;R) \end{aligned}$$
      for all *Q* with $x \in \texttt {fv}(Q)$, as desired.
  2. Suppose that $\ell$ originates in *R*:
    $$\begin{aligned} \varGamma ; \varDelta _1 \cdot \varDelta _3 \vdash (\nu \, \widetilde{m_1})(P_1 \;|\;R) \xrightarrow {\ell } \varDelta _1 \cdot \varDelta _3' \vdash (\nu \, \widetilde{m_1}')(P_1 \;|\;R') \end{aligned}$$
    This case is also divided into three sub-cases:
    i. Sub-case $\ell \notin \{(\nu \, \widetilde{m}) n !\langle \lambda \widetilde{x}.\,Q \rangle , (\nu \, \widetilde{m}\widetilde{m_1}) n !\langle \widetilde{m_1} \rangle \}$: From the LTS we infer that
      $$\begin{aligned} \varGamma ; \varDelta _3 \vdash R \xrightarrow {\ell } \varDelta _3' \vdash R' \end{aligned}$$
      for some $\varDelta _3'$, which in turn implies
      $$\begin{aligned} \varGamma ; \varDelta _2 \cdot \varDelta _3 \vdash (\nu \, \widetilde{m_2})(P_2 \;|\;R) \xrightarrow {\ell } \varDelta _2 \cdot \varDelta _3' \vdash (\nu \, \widetilde{m_2}')(P_2 \;|\;R') \end{aligned}$$
      Now, from the definition of ${\mathcal {S}}$ we may obtain the desired conclusion:
      $$\begin{aligned} \varGamma ; \varDelta _1 \cdot \varDelta _3' \vdash (\nu \, \widetilde{m_1}')(P_1 \;|\;R') \ {\mathcal {S}}\ \varDelta _2 \cdot \varDelta _3' \vdash (\nu \, \widetilde{m_2}')(P_2 \;|\;R') \end{aligned}$$
    ii. Sub-case $\ell = (\nu \, \widetilde{m_1}) n !\langle \lambda \widetilde{x}.\,Q \rangle$: From the LTS we infer that:
      60 $$\begin{aligned} \varGamma ; \varDelta _3 \vdash R \xrightarrow {\ell } \varDelta _3' \vdash R' \end{aligned}$$
      for some $\varDelta _3'$. We then have that
      61 $$\begin{aligned} \varGamma ; \emptyset ; \varDelta _3'' \vdash (\nu \, \widetilde{m}')(R' \;|\;R_1 \lambda \widetilde{x}.\,Q/x) \triangleright \diamond \end{aligned}$$
      for some $\varDelta _3''$ and for all $R_1$ with $\{x\} = \texttt {fv}(R_1)$. Now, from (60) we obtain that
      $$\begin{aligned} \varGamma ; \varDelta _2 \cdot \varDelta _3 \vdash (\nu \, \widetilde{m_2}')(P_2 \;|\;R) \xrightarrow {\ell } \varDelta _2 \cdot \varDelta _3' \vdash (\nu \, \widetilde{m_2})(P_2 \;|\;R') \end{aligned}$$
      Then, from (61) and the definition of ${\mathcal {S}}$ we obtain that
      $$\begin{aligned} \begin{array}{rcll} \varGamma ; \emptyset ; &{}\varDelta _1 \cdot \varDelta _3''&{} \vdash &{} (\nu \, \widetilde{m_1})(P_1 \;|\;(\nu \, \widetilde{m}')(R' \;|\;R_1 \lambda \widetilde{x}.\,Q/x))\\ \ {\mathcal {S}}\ &{}\varDelta _2 \cdot \varDelta _3''&{} \vdash &{} (\nu \, \widetilde{m_2})(P_2 \;|\;(\nu \, \widetilde{m}')(R' \;|\;R_1 \lambda \widetilde{x}.\,Q/x)) \end{array} \end{aligned}$$
      for all $R_1$ with $x \in \texttt {fv}(R_1)$, as desired.
    iii. Sub-case $\ell = (\nu \, \widetilde{m}\widetilde{m_1}) n !\langle \widetilde{m} \rangle$: Similarly as above, from the typed LTS we infer that:
      62 $$\begin{aligned} \varGamma ; \varDelta _3 \vdash R \xrightarrow {\ell } \varDelta _3' \vdash R' \end{aligned}$$
      for some $\varDelta _3'$. We then have that
      63 $$\begin{aligned} \varGamma ; \emptyset ; \varDelta _3'' \vdash (\nu \, \widetilde{m}')(R' \;|\;R_1 \widetilde{m}/\widetilde{x}) \triangleright \diamond \end{aligned}$$
      for all $R_1$ with $\{\widetilde{x}\} = \texttt {fv}(R_1)$, for some $\varDelta _3''$. Now, from (62), we obtain that
      $$\begin{aligned} \varGamma ; \varDelta _2 \cdot \varDelta _3 \vdash (\nu \, \widetilde{m_2})(P_2 \;|\;R) \xrightarrow {\ell } \varDelta _2 \cdot \varDelta _3' \vdash (\nu \, \widetilde{m_2})(P_2 \;|\;R') \end{aligned}$$
      Then, from (63) and the definition of ${\mathcal {S}}$ we obtain the desired conclusion:
      $$\begin{aligned} \begin{array}{rcll} \varGamma ; \emptyset ; &{}\varDelta _1 \cdot \varDelta _3''&{} \vdash &{} (\nu \, \widetilde{m_1})(P_1 \;|\;(\nu \, \widetilde{m}')(R' \;|\;R_1 \widetilde{m}/\widetilde{x}))\\ \ {\mathcal {S}}\ &{}\varDelta _2 \cdot \varDelta _3''&{} \vdash &{} (\nu \, \widetilde{m_2})(P_2 \;|\;(\nu \, \widetilde{m}')(R' \;|\;R_1 \widetilde{m}/\widetilde{x})) \end{array} \end{aligned}$$
  3. We finally suppose that $\ell$ originates from the interaction between $P_1$ and *R*:
    $$\begin{aligned} \varGamma ; \varDelta _1 \cdot \varDelta _3 \vdash (\nu \, \widetilde{m_1})(P_1 \;|\;R) \xrightarrow {\tau } \varDelta _1' \cdot \varDelta _3' \vdash (\nu \, \widetilde{m_1}')(P_1' \;|\;R') \end{aligned}$$
    for some $\varDelta _1', \varDelta _3'$. We then have that
    $$\begin{aligned}\varGamma ; \varDelta _1 \vdash P_1 \xrightarrow {\ell _1} \varDelta _1' \vdash P_1'\end{aligned}$$
    and
    64 $$\begin{aligned} \varGamma ; \varDelta _3 \vdash R \xrightarrow {\ell _2} \varDelta _3 \vdash R' \end{aligned}$$
    with $\ell _1 \asymp \ell _2$ (cf. Definition 3). This case is divided into two sub-cases:
    i. $\ell _1 \notin \{(\nu \, \widetilde{m}) n !\langle \lambda \widetilde{x}.\,Q \rangle , (\nu \, \widetilde{m}\widetilde{m_1}) n !\langle \widetilde{m_1} \rangle \}$: Then the transition from $P_1$ implies
      65 $$\begin{aligned}&\varGamma ; \varDelta _2 \vdash P_2 \mathop {\Longrightarrow }\limits ^{\hat{\ell _1}} \varDelta _2' \vdash P_2' \end{aligned}$$
      66 $$\begin{aligned}&\varGamma ; \varDelta _1' \vdash P_1' \approx \varDelta _2' \vdash P_2' \end{aligned}$$
      for some $\varDelta _2'$. From (64) and (65) we obtain
      $$\begin{aligned} \varGamma ; \varDelta _2 \cdot \varDelta _3 \vdash (\nu \, \widetilde{m_2})(P_2 \;|\;R) \mathop {\Longrightarrow }\limits ^{} \varDelta _2' \cdot \varDelta _3' \vdash (\nu \, \widetilde{m_2}')(P_2' \;|\;R') \end{aligned}$$
      Then, from (66) and the definition of ${\mathcal {S}}$ we obtain the desired conclusion:
      $$\begin{aligned} \varGamma ; \varDelta _1' \cdot \varDelta _3' \vdash (\nu \, \widetilde{m_1}')(P_1' \;|\;R') \ {\mathcal {S}}\ \varDelta _2' \cdot \varDelta '_3 \vdash (\nu \, \widetilde{m_2}')(P_2' \;|\;R') \end{aligned}$$
    ii. $\ell _1 = {(\nu \, \widetilde{m_1}) n !\langle V_1 \rangle }$: Then we have the transition
      $$\begin{aligned} \varGamma ; \varDelta _1 \vdash P_1 \xrightarrow {(\nu \, \widetilde{m_1}) n !\langle V_1 \rangle } \varDelta _1' \vdash P_1' \end{aligned}$$
      for some $\varDelta '_1$, which implies
      67 $$\begin{aligned}&\varGamma ; \varDelta _3 \vdash R \xrightarrow {n ?\langle V_1 \rangle } \varDelta _3' \vdash R' V_1/x \end{aligned}$$
      68 $$\begin{aligned}&\varGamma ; \varDelta _1 \cdot \varDelta _3 \vdash (\nu \, \widetilde{m_1})(P_1 \;|\;R) \xrightarrow {\tau } \varDelta _1' \cdot \varDelta _3' \vdash (\nu \, \widetilde{m_1}'')(P_1' \;|\;R' V_1/x) \end{aligned}$$
      for some $\varDelta _1'$ and $\varDelta '_3$. In turn, the output transition from $P_1$ implies the existence of $\varDelta _2'$, $Q_2$, $P'_2$ such that
      69 $$\begin{aligned}&\varGamma ; \varDelta _2 \vdash P_2 \mathop {\Longrightarrow }\limits ^{(\nu \, \widetilde{m_2}) n !\langle V_2 \rangle } \varDelta _2' \vdash P_2' \end{aligned}$$
      70 $$\begin{aligned}&2 \varGamma ; \varDelta _1'' \vdash (\nu \, \widetilde{m_1}')(P_1' \;|\;R' V_1/x) \ \approx \ \varDelta _2'' \vdash (\nu \, \widetilde{m_2}')(P_2' \;|\;R' V_2/x) \end{aligned}$$
      for all $R'$ with $\{x\} = \texttt {fv}(Q)$, and for some $\varDelta _1''$ and $\varDelta _2''$. From (67) we obtain
      $$\begin{aligned} \varGamma ; \varDelta _3 \vdash R \xrightarrow {n ?\langle V_2 \rangle } \varDelta _3'' \vdash R' V_2/x \end{aligned}$$
      for some $\varDelta _3''$, which may be combined with (69) to obtain
      $$\begin{aligned} \varGamma ; \varDelta _2 \cdot \varDelta _3 \vdash (\nu \, \widetilde{m_2})(P_2 \;|\;R) \mathop {\Longrightarrow }\limits ^{} \varDelta _2' \cdot \varDelta _3'' \vdash (\nu \, \widetilde{m_2}'')(P_2' \;|\;R' V_2/x) \end{aligned}$$
      From (70) and the definition of ${\mathcal {S}}$ we can then get:
      $$\begin{aligned} \varGamma ; \varDelta _1'' \vdash (\nu \, \widetilde{m_1}')(P_1' \;|\;R' V_1/x) \ {\mathcal {S}}\ \varDelta _2'' \vdash (\nu \, \widetilde{m_2}')(P_2' \;|\;R' V_2/x). \end{aligned}$$
      as required.$$\square $$

In order to prove Lemma 7 (i.e., $\cong \ \subseteq \ \approx ^\mathtt{H}$), below we follow the technique developed in [8] and refined for session types in [18, 19].

#### Definition 25

(*Definability*) Let $\varGamma ; \emptyset ; \varDelta _1 \vdash P \triangleright \diamond$. A visible action $\ell$ is *definable* whenever, given a fresh name $succ$, there exists a (testing) process $\varGamma ; \emptyset ; \varDelta _2 \vdash T\langle \ell , succ \rangle \triangleright \diamond$ such that:

1. If  then, for some $\varDelta _2'$, either
  - $$\ell \ne (\nu \, \widetilde{m})n !\langle V \rangle $$ and $$P \;|\;T\langle \ell , succ \rangle \longrightarrow P' \;|\;succ !\langle \overline{n} \rangle . \mathbf {0}$$ and
    $$\varGamma ; \emptyset ; \varDelta _1' \cdot \varDelta _2' \vdash P' \;|\;succ !\langle \overline{n} \rangle . \mathbf {0}\triangleright \diamond $$
  - $$\ell = (\nu \, \widetilde{m})n !\langle V \rangle $$ and $$P \;|\;T\langle \ell , succ \rangle \longrightarrow (\nu \, \widetilde{m})(P' \;|\;t \hookleftarrow _{\texttt {H}} V \;|\;succ !\langle \overline{n}, V \rangle . \mathbf {0})$$ and
    $\varGamma ; \emptyset ; \varDelta _1' \cdot \varDelta _2' \vdash (\nu \, \widetilde{m})(P' \;|\;t \hookleftarrow _{\texttt {H}} V \;|\;succ !\langle \overline{n}, V \rangle . \mathbf {0}) \triangleright \diamond$, for some fresh *t*.
2. If $P \;|\;T\langle \ell , succ \rangle \longrightarrow Q$ with $\varGamma ; \emptyset ; \varDelta \vdash Q \downarrow _{succ}$ then there exists a $P'$ such that  and one of the following holds:
  - $$\ell \ne (\nu \, \widetilde{m})n !\langle V \rangle $$ and $$Q \equiv P' \;|\;succ !\langle \overline{n} \rangle . \mathbf {0}$$.
  - $\ell = (\nu \, \widetilde{m})n !\langle V \rangle$ and $Q \equiv (\nu \, \widetilde{m})(P' \;|\;t \hookleftarrow _{\texttt {H}} V \;|\;succ !\langle \overline{n}, V \rangle . \mathbf {0})$, for some fresh *t*.

We first show that every visible action $\ell$ is definable.

#### Lemma 20

(Definability) Every visible action $\ell$ is definable.

#### Proof

Let $succ$ be a fresh name. We define:

$$ \begin{aligned} T\langle \ell , succ \rangle = {\left\{ \begin{array}{ll} \overline{n} !\langle V \rangle . succ !\langle \overline{n} \rangle . \mathbf {0}&{} \hbox { if}\ \ell = n ?\langle V \rangle \\ \overline{n} \triangleleft l . succ !\langle \overline{n} \rangle . \mathbf {0}&{} \hbox { if}\ \ell = n \, \& \, l \\ \overline{n} ?(y) . (t \hookleftarrow _{\texttt {H}} y \;|\;succ !\langle \overline{n}, y \rangle . \mathbf {0}) &{} \hbox { if}\ \ell = (\nu \, \widetilde{m}) n !\langle V \rangle \\ \overline{n} \triangleright \{l: succ !\langle \overline{n} \rangle . \mathbf {0}), l_i: (\nu \, a)(a ?(y) . succ !\langle \overline{n} \rangle . \mathbf {0})\}_{i \in I} &{} \hbox { if}\ \ell = n \oplus l \end{array}\right. } \end{aligned}$$

Consider the process

$$\begin{aligned} \varGamma ; \emptyset ; \varDelta \vdash P \triangleright \diamond \end{aligned}$$

It is straightforward to do a case analysis on all actions $\ell$ such that

to show that $\ell$ is definable. $\square$

We rely on the following auxiliary result:

#### Lemma 21

(Extrusion) Let *P* and *Q* be processes, and let $succ$ be a fresh name. If

$$\begin{aligned} \varGamma ; \varDelta _1' \vdash (\nu \, \widetilde{m_1})(P \;|\;succ !\langle \overline{n}, V_1 \rangle . \mathbf {0}) \cong \varDelta _2 \vdash (\nu \, \widetilde{m_2})(Q \;|\;succ !\langle \overline{n}, V_2 \rangle . \mathbf {0}) \end{aligned}$$

with $\{\widetilde{m_1}\}= \texttt {fn}(V_1)$ and $\{\widetilde{m_2}\} = \texttt {fn}(V_2)$ then there exist $\varDelta _1$ and $\varDelta _2$ such that

$$\begin{aligned} \varGamma ; \varDelta _1 \vdash P \cong \varDelta _2 \vdash Q. \end{aligned}$$

#### Proof

Let ${\mathcal {S}}$ be a relation defined as:

$$\begin{aligned} {\mathcal {S}}= & {} \{(\varGamma ; \emptyset ; \varDelta _1 \vdash P \triangleright \diamond \ ,\ \varGamma ; \emptyset ; \varDelta _2 \vdash Q \triangleright \diamond ) \ \ |\ \ \\&\qquad \varGamma ; \varDelta _1' \vdash (\nu \, \widetilde{m_1})(P \;|\;succ !\langle \overline{n}, V_1 \rangle . \mathbf {0}) \cong \varDelta '_2 \vdash (\nu \, \widetilde{m_2})(Q \;|\;succ !\langle \overline{n}, V_2 \rangle . \mathbf {0}),\\&\qquad \wedge ~ m_1 \in \texttt {fn}(V_1) \wedge m_2 \in \texttt {fn}(V_2) \} \end{aligned}$$

We show that ${\mathcal {S}}$ is a reduction-closed, barbed congruence.

I. *Reduction-closed* The reduction $P \longrightarrow P'$ implies
  $$\begin{aligned} (\nu \, \widetilde{m_1})(P \;|\;succ !\langle \overline{n}, V_1 \rangle . \mathbf {0}) \longrightarrow (\nu \, \widetilde{m_1})(P' \;|\;succ !\langle \overline{n}, V_1 \rangle . \mathbf {0}) \end{aligned}$$
  which, due to freshness of $succ$, in turn implies
  $$\begin{aligned} (\nu \, \widetilde{m_1})(Q \;|\;succ !\langle \overline{n}, V_2 \rangle . \mathbf {0}) \longrightarrow ^{*} (\nu \, \widetilde{m_1})(Q' \;|\;succ !\langle \overline{n}, V_2 \rangle . \mathbf {0}) \end{aligned}$$
  Therefore, $$Q \longrightarrow ^{*} Q'$$. Furthermore,
  $$\begin{aligned} (\nu \, \widetilde{m_1})(P' \;|\;succ !\langle \overline{n}, V_1 \rangle . \mathbf {0}) \cong (\nu \, \widetilde{m_1})(Q' \;|\;succ !\langle \overline{n}, V_2 \rangle . \mathbf {0}) \end{aligned}$$
  that implies
  $$\begin{aligned} \varGamma ; \varDelta _1'' \vdash P'\ {\mathcal {S}}\ \varDelta _2'' \vdash Q' \end{aligned}$$
  as required.
II. *Barb preserving* Suppose $\varGamma ; \emptyset ; \varDelta _1 \vdash P \downarrow _{m}$. We analyse three cases, depending on the nature of *m*:
  1. Case $m \not = s$ (*m* is not a session name): Then from $\varGamma ; \emptyset ; \varDelta _1 \vdash P \downarrow _{m}$ we infer
    $$\begin{aligned} \varGamma ; \emptyset ; \varDelta _1' \vdash (\nu \, \widetilde{m_1})(P \;|\;succ !\langle \overline{n}, V_1 \rangle . \mathbf {0}) \downarrow _{m} \end{aligned}$$
    for some $\varDelta _1'$, which implies
    $$\begin{aligned} \varGamma ; \emptyset ; \varDelta _2' \vdash (\nu \, \widetilde{m_2})(Q \;|\;succ !\langle \overline{n}, V_2 \rangle . \mathbf {0}) \Downarrow _{m}. \end{aligned}$$
    for some $\varDelta _2'$. Then, from the freshness of $succ$, we obtain $\varGamma ; \emptyset ; \varDelta _2 \vdash Q \Downarrow _{m}$, as required.
  2. Case: $m = s$ (*m* is a session name) and $m \not = n$. The proof follows a similar reasoning as in the previous case.
  3. Case: $m = s$ (*m* is a session name) and $m = n$ and $\varGamma ; \emptyset ; \varDelta _1 \vdash P \downarrow _{n}$. In this case, the fact that *n* is a session name implies that $n, \overline{n} \in \texttt {dom}(\varDelta _1')$. Therefore, from the definition of barbs (Definition 9) we can infer that
    because both endpoints of session *n* are present in $\varDelta _1'$.
    To observe the desired barb we exploit an additional test process, with an extra fresh name $succ'$. We compose $\varGamma ; \emptyset ; \varDelta _1 \vdash P \triangleright \diamond$ with $\overline{succ} ?(x, y) . T\langle \ell , succ' \rangle$ where $\texttt {subj}(\ell ) = x$. We then have
    $$\begin{aligned} \varGamma ; \emptyset ; \varDelta _1' \vdash (\nu \, \widetilde{m_1})(P \;|\;succ !\langle \overline{n}, V_1 \rangle . \mathbf {0}) \;|\;\overline{succ} ?(x, y) . T\langle \ell , succ' \rangle \triangleright \diamond \end{aligned}$$
    The definition of definability and the fact that $\varGamma ; \emptyset ; \varDelta _1 \vdash P \downarrow _{n}$ imply that
    $$\begin{aligned}&(\nu \, \widetilde{m_1})(P \;|\;succ !\langle \overline{n}, V_1 \rangle . \mathbf {0}) \;|\;\overline{succ} ?(x, {y}) . T\langle \ell , succ' \rangle \\&\quad \longrightarrow ^{*} (\nu \, \widetilde{m_1})(P' \;|\;succ' !\langle \overline{n}, V_1' \rangle . \mathbf {0}) \end{aligned}$$
    and furthermore
    $$\begin{aligned}&(\nu \, \widetilde{m_2})(Q \;|\;succ !\langle \overline{n}, V_2 \rangle . \mathbf {0}) \;|\;\overline{succ} ?(x, {y}) . T\langle \ell , succ' \rangle \\&\quad \longrightarrow ^{*} (\nu \, \widetilde{m_2})(Q' \;|\;succ' !\langle \overline{n}, V_2' \rangle . \mathbf {0}) \end{aligned}$$
    The last sequence of reductions implies that $\varGamma ; \emptyset ; \varDelta _2 \vdash Q \Downarrow _{n}$, as required.
III. *Congruence* The key case is congruence with respect to parallel composition. The other cases are easier due to the fact that we are working with closed process terms (i.e. input congruence is straightforward on closed process terms). Let us define relation ${\mathcal {C}}$ as
  $$\begin{aligned} {\mathcal {C}}= & {} \{ (\varGamma ; \emptyset ; \varDelta _1 \cdot \varDelta _3 \vdash P \;|\;R \triangleright \diamond , \varGamma ; \emptyset ; \varDelta _2 \cdot \varDelta _3 \vdash Q \;|\;R \triangleright \diamond ) \ \ |\ \ \\&\qquad \forall R \text { such that } \exists \varDelta _3, \varGamma ;\emptyset ; \varDelta _3 \vdash R \triangleright \diamond \wedge \\&\qquad \varGamma ; \varDelta _1' \vdash (\nu \, \widetilde{m_1})(P \;|\;succ !\langle \overline{n}, V_1 \rangle . \mathbf {0}) \cong \varDelta _2' \vdash (\nu \, \widetilde{m_2})(Q \;|\;succ !\langle \overline{n}, V_2 \rangle . \mathbf {0})\} \end{aligned}$$
  We want to show that ${\mathcal {C}} \subseteq {\mathcal {S}}$. To this end, we show that ${\mathcal {C}}$ is a congruence with respect to parallel composition. We distinguish two cases:
  (i) Case $(\overline{n} \cup \texttt {fn}(V_1) \cup \texttt {fn}(V_2)) \cap \texttt {fn}(R) = \emptyset$: Then from the contextual definition of $\cong$ we can deduce that for all $\varGamma ; \emptyset ; \varDelta _3 \vdash R \triangleright \diamond$:
    $$\begin{aligned}&\varGamma ; \varDelta _1' \cdot \varDelta _3 \vdash (\nu \, \widetilde{m_1})(P \;|\;succ !\langle \overline{n}, V_1 \rangle . \mathbf {0}) \;|\;R \cong \\ \vdash&\quad {\varDelta _2' \cdot \varDelta _3}{(\nu \, \widetilde{m_2})(Q \;|\;succ !\langle \overline{n}, V_2 \rangle . \mathbf {0}) \;|\;R} \end{aligned}$$
    Because of the requirement $(\overline{n} \cup \texttt {fn}(V_1) \cup \texttt {fn}(V_2)) \cap \texttt {fn}(R) = \emptyset$ the above is structurally congruent to
    $$\begin{aligned}&\varGamma ; \varDelta _1' \cdot \varDelta _3 \vdash (\nu \, \widetilde{m_1})(P \;|\;succ !\langle \overline{n}, V_1 \rangle . \mathbf {0}\;|\;R)\\&\quad \cong \varDelta _2' \cdot \varDelta _3 \vdash (\nu \, \widetilde{m_2})(Q \;|\;succ !\langle \overline{n}, V_2 \rangle . \mathbf {0}\;|\;R) \end{aligned}$$
    The desired conclusion is then immediate from the definition of ${\mathcal {C}}$.
  (ii) Case $\widetilde{s} = \{\overline{n}, \widetilde{m_1}\} \cup \{\overline{n}, \widetilde{m_2}\} \cap \texttt {fn}(R)$: Let $R^{\widetilde{y}}$ be such that $R = R^{\widetilde{y}}\widetilde{s}/\widetilde{y}$.
    From the contextual definition of $\cong$, given a fresh name $succ'$, we can deduce that for all $\varGamma ; \emptyset ; \varDelta _3' \vdash \overline{succ} ?(\widetilde{y}) . (R^{\widetilde{y}} \;|\;succ' !\langle \widetilde{y} \rangle . \mathbf {0}) \triangleright \diamond$:
    $$\begin{aligned} \begin{array}{rcll} \varGamma ; \emptyset ; &{}\varDelta _1''&{} \vdash &{} (\nu \, \widetilde{m_1})(P \;|\;succ !\langle \overline{n}, V_1 \rangle . \mathbf {0}) \;|\;\overline{succ} ?(\widetilde{y}) . (R^{\widetilde{y}} \;|\;succ' !\langle \widetilde{y} \rangle . \mathbf {0})\\ \cong &{}\varDelta _2''&{} \vdash &{} (\nu \, \widetilde{m_2})(Q \;|\;succ !\langle \overline{n}, V_2 \rangle . \mathbf {0}) \;|\;\overline{succ} ?(\widetilde{y}) . (R^{\widetilde{y}} \;|\;succ' !\langle \widetilde{y} \rangle . \mathbf {0}) \end{array} \end{aligned}$$
    for some $\varDelta _1'', \varDelta _2''$. Applying reduction closeness to the above pair we infer:
    $$\begin{aligned} \varGamma ; \varDelta _1'' \vdash (\nu \, \widetilde{m_1})(P \;|\;R \;|\;succ' !\langle \overline{n}, V_1 \rangle . \mathbf {0}) \cong \varDelta _2'' \vdash (\nu \, \widetilde{m_2})(Q \;|\;R \;|\;succ' !\langle \overline{n}, V_2 \rangle . \mathbf {0}) \end{aligned}$$
    The conclusion then follows from the definition of ${\mathcal {C}}$.$\square$

We can finally prove Lemma 7:

#### Lemma 22

$$\cong \ \subseteq \ \approx ^\mathtt{H}$$.

#### Proof

Let $\mathfrak {R}$ be the typed relation (we omit the typing information in the definition):

$$\begin{aligned} \mathfrak {R}= \{(P_1, P_2) \ \ |\ \ \varGamma ; \varDelta _1 \vdash P_1 \cong \varDelta _2 \vdash P_2\} \end{aligned}$$

We prove that $\mathfrak {R}$ is a higher-order bisimulation. Suppose that $\varGamma ; \varDelta _1 \vdash P_1 \xrightarrow {\ell } \varDelta _1' \vdash P_1'$; we must find a matching action from $P_2$. We distinguish two cases:

1. Suppose $\ell = \tau$. Then we have
  $$\begin{aligned} \varGamma ; \varDelta _1 \vdash P_1 \xrightarrow {\tau } \varDelta _1' \vdash P_1' \end{aligned}$$
  The result follows the reduction closeness property of $\cong$ since
  $$\begin{aligned} \varGamma ; \varDelta _2 \vdash P_2 \mathop {\Longrightarrow }\limits ^{\tau } \varDelta _2' \vdash P_2' \end{aligned}$$
  for some $\varDelta _2'$, and
  $$\begin{aligned} \varGamma ; \varDelta _1' \vdash P_1' \cong \varDelta _2' \vdash P_2' \text { implies } \varGamma ; \varDelta _1' \vdash P_1' \ \mathfrak {R}\ \varDelta _2' \vdash P_2'. \end{aligned}$$
2. Suppose $\ell \ne \tau$. Then we choose test $T\langle \ell , succ \rangle$ to obtain
  71 $$\begin{aligned} \varGamma ; \varDelta _1 \cdot \varDelta _3 \vdash P_1 \;|\;T\langle \ell , succ \rangle \cong \varDelta _2 \cdot \varDelta _3 \vdash P_2 \;|\;T\langle \ell , succ \rangle \end{aligned}$$
  for some $\varDelta _3$. From this point on we distinguish two sub-cases:
  i. Sub-case $\ell \in \{n ?\langle V_1 \rangle , n \oplus l, n \, \& \, l\}$: We then obtain
    $$\begin{aligned}&P_1 \;|\;T\langle \ell , succ \rangle \longrightarrow P_1' \;|\;succ !\langle \overline{n} \rangle . \mathbf {0}\\&\varGamma ; \emptyset ; \varDelta _1' \cdot \varDelta _3' \vdash P_1' \;|\;succ !\langle \overline{n} \rangle . \mathbf {0}\downarrow _{succ} \end{aligned}$$
    for some $\varDelta '_3$. From (71) we may now infer:
    $$\begin{aligned}&\varGamma ; \emptyset ; \varDelta _2 \cdot \varDelta _3 \vdash P_2 \;|\;T\langle \ell , succ \rangle \Downarrow _{succ} \end{aligned}$$
    which, using Lemma 20, implies
    $$\begin{aligned}&{\varGamma }{\varDelta _2}{P_2}\,\,{\mathop {\Longrightarrow }\limits ^{\ell }}\,\,{\varDelta _2'}{P_2'}\\&P_2 \;|\;T \langle \ell , succ \rangle \longrightarrow ^{*} P_2' \;|\;succ !\langle \overline{n} \rangle . \mathbf {0}\end{aligned}$$
    and
    $$\begin{aligned} \varGamma ; \varDelta _1' \cdot \varDelta _3' \vdash P_1' \;|\;succ !\langle \overline{n} \rangle .\mathbf {0} \cong \varDelta _2' \cdot \varDelta _3' \vdash P_2' \;|\;succ !\langle \overline{n} \rangle . \mathbf {0} \end{aligned}$$
    We then apply Lemma 21 to obtain the required result:
    $$\begin{aligned} \varGamma ; \varDelta _1' \vdash P_1' \cong \varDelta _2' \vdash P_2' \text { implies } \varGamma ; \varDelta _1' \vdash P_1' \ \mathfrak {R}\ \varDelta _2' \vdash P_2'. \end{aligned}$$
  ii. Sub-case $\ell = (\nu \, \widetilde{m_1}) n !\langle V_1 \rangle$: Note that $T\langle (\nu \, \widetilde{m_1}) n !\langle V_1 \rangle , succ \rangle = T\langle (\nu \, \widetilde{m_2}) n !\langle V_2 \rangle , succ \rangle$. The transition from $P_1$ can be then written as
    72 $$\begin{aligned} \varGamma ; \varDelta _1 \vdash P_1 \xrightarrow {(\nu \, \widetilde{m_1}) n !\langle V_1 \rangle } \varDelta _1' \vdash P_1' \end{aligned}$$
    for some $\varDelta _1'$. If we use the test process $T\langle (\nu \, \widetilde{m_1}) n !\langle V_1 \rangle , succ \rangle$, then we may obtain:
    $$\begin{aligned}&P_1 \;|\;T\langle (\nu \, \widetilde{m_1}) n !\langle V_1 \rangle , succ \rangle \longrightarrow (\nu \, \widetilde{m_1})(P_1' \;|\;t \hookleftarrow _{\texttt {H}} V_1) \;|\;succ !\langle \overline{n}, V_1 \rangle . \mathbf {0}\\&\varGamma ; \emptyset ; \varDelta _1' \cdot \varDelta _3' \vdash (\nu \, \widetilde{m_1})(P_1' \;|\;t \hookleftarrow _{\texttt {H}} V_1) \;|\;succ !\langle \overline{n}, V_1 \rangle . \mathbf {0}\downarrow _{succ} \end{aligned}$$
    for some $\varDelta '_3$. Using (71) we may then infer
    $$\begin{aligned} \varGamma ; \emptyset ; \varDelta _2 \cdot \varDelta _3 \vdash P_2 \;|\;T\langle (\nu \, \widetilde{m_2}) n !\langle V_2 \rangle , succ \rangle \Downarrow _{succ} \end{aligned}$$
    which, using Lemma 20, implies
    73 $$\begin{aligned}&\varGamma ; \varDelta _2 \vdash P_2 \mathop {\Longrightarrow }\limits ^{(\nu \, \widetilde{m_2}) n !\langle V_2 \rangle } \varDelta _2' \vdash P_2' \nonumber \\&P_2 \;|\;T \langle \ell , succ \rangle \longrightarrow ^{*} (\nu \, \widetilde{m_2})(P_2' \;|\;t \hookleftarrow _{\texttt {H}} V_2) \;|\;succ !\langle \overline{n}, V_2 \rangle . \mathbf {0}\end{aligned}$$
    for some $\varDelta _2'$, and
    $$\begin{aligned} \begin{array}{rcll} \varGamma ; \emptyset ; &{}\varDelta _1' \cdot \varDelta _3'&{} \vdash &{} (\nu \, \widetilde{m_1})(P_1' \;|\;t \hookleftarrow _{\texttt {H}} \lambda \widetilde{x}.\,Q_1) \;|\;succ !\langle \overline{n}, V_1 \rangle . \mathbf {0}\\ \cong &{}\varDelta _2' \cdot \varDelta _3'&{} \vdash &{} (\nu \, \widetilde{m_2})(P_2' \;|\;t \hookleftarrow _{\texttt {H}} \lambda \widetilde{x}.\,Q_2) \;|\;succ !\langle \overline{n}, V_2 \rangle . \mathbf {0} \end{array} \end{aligned}$$
    We then apply Lemma 21 to obtain:
    $$\begin{aligned} \varGamma ; \varDelta _1' \vdash (\nu \, \widetilde{m_1})(P_1' \;|\;t \hookleftarrow _{\texttt {H}} V_1) \cong \varDelta _2' \vdash {(\nu \, \widetilde{m_2})(P_2' \;|\;t \hookleftarrow _{\texttt {H}} V_2)} \end{aligned}$$
    From the above result and the definition of $\mathfrak {R}$ we finally obtain:
    $$\begin{aligned} \begin{array}{rcll} \varGamma ; \emptyset ; &{}\varDelta _1'&{} \vdash &{} (\nu \, \widetilde{m_1})(P_1' \;|\;t \hookleftarrow _{\texttt {H}} V_1)\\ \ \mathfrak {R}\ &{}\varDelta _2'&{} \vdash &{} (\nu \, \widetilde{m_2})(P_2' \;|\;t \hookleftarrow _{\texttt {H}} V_2) \end{array} \end{aligned}$$
    as required.$$\square $$
